# Pharmacodynamic Consequences of Administration of VLA-4 Antagonist CDP323 to Multiple Sclerosis Subjects: A Randomized, Double-Blind Phase 1/2 Study

**DOI:** 10.1371/journal.pone.0058438

**Published:** 2013-03-05

**Authors:** Christian Wolf, Jagdev Sidhu, Christian Otoul, Dexter L. Morris, Jennifer Cnops, Jorg Taubel, Barbara Bennett

**Affiliations:** 1 Clinical Development, Lycalis s.p.r.l., Brussels, Belgium; 2 Clinical Pharmacology/Early Development, CSL Ltd., Melbourne, Australia; 3 Exploratory Statistics, UCB Pharma, Brabant, Belgium; 4 Drug Development, UCB Biosciences Inc., Raleigh, North Carolina, United States of America; 5 Department of Cellular and Molecular Immunology, Vrije Universiteit Brussel, Brussels, Belgium; 6 Richmond Pharmacology, London, United Kingdom; 7 BABennett Consulting, Marietta, Georgia, United States of America; Washington University, United States of America

## Abstract

**Background:**

Lymphocyte inhibition by antagonism of α4 integrins is a validated therapeutic approach for relapsing multiple sclerosis (RMS).

**Objective:**

Investigate the effect of CDP323, an oral α_4_-integrin inhibitor, on lymphocyte biomarkers in RMS.

**Methods:**

Seventy-one RMS subjects aged 18–65 years with Expanded Disability Status Scale scores ≤6.5 were randomized to 28-day treatment with CDP323 100 mg twice daily (bid), 500 mg bid, 1000 mg once daily (qd), 1000 mg bid, or placebo.

**Results:**

Relative to placebo, all dosages of CDP323 significantly decreased the capacity of lymphocytes to bind vascular adhesion molecule-1 (VCAM-1) and the expression of α_4_-integrin on VCAM-1–binding cells. All but the 100-mg bid dosage significantly increased total lymphocytes and naive B cells, memory B cells, and T cells in peripheral blood compared with placebo, and the dose-response relationship was shown to be linear. Marked increases were also observed in natural killer cells and hematopoietic progenitor cells, but only with the 500-mg bid and 1000-mg bid dosages. There were no significant changes in monocytes. The number of samples for regulator and inflammatory T cells was too small to draw any definitive conclusions.

**Conclusions:**

CDP323 at daily doses of 1000 or 2000 mg induced significant increases in total lymphocyte count and suppressed VCAM-1 binding by reducing unbound very late antigen-4 expression on lymphocytes.

**Trial Registration:**

ClinicalTrials.gov NCT00726648.

## Introduction

Multiple sclerosis (MS) is a chronic, disabling autoimmune disease of the central nervous system (CNS) characterized by inflammation, demyelination, and axonal destruction [1]. Relapsing MS (RMS) has a characteristic pathology of intermittent development of multifocal inflammatory lesions in the brain and spinal cord thought to result from activated lymphocytes breaching the blood-brain barrier [2]–[4]. Inflammatory mediators such as tumor necrosis factor-alpha, which is produced during inflammatory responses, induce vascular endothelial cells to express vascular adhesion molecule-1 (VCAM-1) [5]. The diapedesis of lymphocytes into the CNS is dependent on lymphocyte adhesion to vascular endothelial cells. This adhesion is mediated by the binding of integrins such as very late antigen-4 (VLA-4 or α_4_β_1_ integrin), expressed on activated lymphocytes, to VCAM-1 [5]–[8].

Inhibition of lymphocyte trafficking by antagonism of α_4_ integrins is a validated therapeutic approach for inflammatory diseases such as MS [9]. Clinical studies with natalizumab, a recombinant humanized monoclonal antibody that targets α_4_β_1_ integrin, have confirmed that this integrin is an effective therapeutic target for relapsing forms of MS [10]. Through selective inhibition of α_4_β_1_ integrin, natalizumab effectively disrupts migration of immune cells and reduces subsequent CNS inflammation [6], [11], [12]. Therapeutically, this results in reduced numbers of inflammatory brain lesions, lower exacerbation rates, and delayed progression of MS disability [10], [12], [13].

Small-molecule α_4_ antagonists such as CDP323 (UCB Pharma, Brussels, Belgium, and Biogen Idec, Weston, MA, USA), a phenylalanine enamide mixed α_4_ antagonist [9], have been considered potential alternatives to therapeutic antibodies because of their increased selectivity for specific α_4_-containing integrin complexes, their inhibition of different affinity states, and the increased patient convenience of oral dosing [14]–[16]. In addition, their short half-lives compared with those of antibodies permits relatively fast elimination from the body in the event of serious side effects that necessitate termination of therapy.

Preclinical investigations have shown that CDP323 possesses anti-inflammatory properties [6], [11], and phase 1 data in human volunteers have suggested that CDP323 is a potent and effective α_4_ inhibitor that is well tolerated at oral doses up to 1000 mg twice daily (bid) [17]. The 1000-mg bid CDP323 dosage appears to have a similar effect on peripheral lymphocyte counts in healthy subjects as natalizumab does at doses of 3 and 6 mg/kg in subjects with MS [18]. However, no data have been gathered on the effects of CDP323 on peripheral lymphocyte counts. The magnitude of increased total lymphocyte counts induced by an α_4_ antagonist may be indicative of its potential to inhibit lymphocyte trafficking and, therefore, predictive of its therapeutic potential, though this has not been formally validated.

When the current study was designed, a 24-week serial MRI phase 2 study investigating two dosages of CDP323 in RMS subjects was ongoing [19]. The study reported herein was intended to complement data on the exposure-response relationship (ie, the 24-week study) by using CDP323 in a wider dosage range and examining different lymphocyte subsets over a 4-week period in subjects with RMS. The objectives of the present study were to identify and support a minimally effective dosage of CDP323 in RMS therapy (as assessed by biomarker changes) and to collect additional information on the biomarker effects and safety of a previously unused higher dosage (1000 mg bid).

In addition, this study investigated CDP323 1000 mg given once daily (qd) in order to characterize how a partial recovery of VCAM-1 binding during a 24-hour dosing interval would affect the trafficking of lymphocytes. A working hypothesis was that a partial recovery might offer safety advantages.

## Methods

This was a hybrid phase 1/phase 2, double-blind, randomized, parallel-group, placebo-controlled study conducted at a single center in London (UK). Male and female subjects aged 18 to 65 years with a diagnosis of RMS, an Expanded Disability Status Scale (EDSS) score of ≤6.5, one relapse within the last 24 months, and no other conditions that could potentially compromise the immune response were recruited to the study. Subjects were excluded if, prior to study screening, they had received immunomodulating or immunosuppressive drugs within 30 days (for interferons, glatiramer acetate, corticosteroids, adrenocorticotropic hormone, or recombinant cytokines), 6 months (for azathioprine, cyclosporine A, or human antibodies), or 12 months (for natalizumab, rituximab, or mitoxantrone); if they had ever been treated with cyclophosphamide; or if they had received total lymphoid irradiation, any antilymphocyte monoclonal antibody therapy, or any inoculation with attenuated live vaccines in the 30 days prior to screening. Subjects were not permitted to take any form of concomitant immunosuppressive or immunomodulatory therapy during the study or for a period of 30 days following completion or withdrawal from the study.

Subjects were randomized to receive placebo or CDP323 at dosages of 100, 500, or 1000 mg qd or 1000 mg bid for a period of 28 consecutive days (day 1 to day 28; last dose given as a morning dose). Treatment was supplied as hard-gelatin capsules containing 50 mg or 250 mg of CDP323 or matched placebo.

Subjects were hospitalized for the first 5 days following initiation of treatment and for the last 2 nights of the treatment period. Pharmacodynamic (PD) assessments were conducted during the predose phase (screening; day −1 at 08∶00, 09∶00, 10∶00, 12∶00, 16∶00, and 20∶00 hours), during the first day of study medication administration (day 1: predose and 1, 2, 4, 8, 12, and 24 hours post-dose), on day 15 (predose), and after the final dose on day 28 (predose and 1, 2, 4, 8, 12, 24, 48, 72, 96, and 168 hours post-dose). Assessments consisted of measurement of absolute counts of total lymphocytes, B cells, T cells, monocytes, hematopoietic progenitor cells, natural killer (NK) cells, NK T cells, regulator T cells (T_reg_), and inflammatory T cells. Expression of α_4_ was also recorded for each subset of lymphocytes except for inflammatory T cells. In addition, the effect of CDP323 on VCAM-1 inhibition was investigated as well.

The protocol for this trial and supporting CONSORT checklist are available as supporting information; see Checklist S1 and Protocol S1.

### Flow Cytometry Methodology

All samples were analyzed by Esoterix Clinical Trial Services (Mechelen, Belgium). White blood cell and differential white blood cell counts were obtained from each whole blood sample using a hematological analyzer before staining and analysis. A 100-µL sample of sodium heparin anticoagulated whole blood from each patient was mixed with appropriate amounts of fluorescent monoclonal antibody cocktails, CD3 peridinin chlorophyll protein (perCP), or CD19 perCP antibody complex and incubated in the dark for 20 minutes. To each tube, 4 mL of a warm lysing solution was added. Samples were mixed and allowed to stand in the dark for a further 5 minutes. After washing, pellets were fixated with 500 µL of 1% paraformaldehyde. Data were acquired with a FACSCalibur™ (BD Biosciences, Erembodegem, Belgium) and analyzed with CellQuest™ software (BD Biosciences). Fluorescence markers for each subject were optimized to assess nonspecific staining.

### Statistical Analysis

Because of the exploratory nature of this study, no formal sample size computation was performed. A sample of 15 subjects per group was considered adequate to describe the effects of CDP323 upon biomarkers across the three tested dosage levels.

Statistical analysis was performed using SAS® software version 9.1 (SAS Institute, Cary, NC, USA). The analysis was performed on the per protocol (PP) population, a subset of subjects without any major protocol deviations affecting the PD evaluation defined prior to database lock and unblinding. Missing values were not imputed. Each leukocyte subset (raw absolute count and percentage change from baseline [defined as measurements collected on day −1]) was analyzed separately and summarized by treatment group and time-point measurements or by day (mean over 24 hours duration) using graphical displays and summary statistics (mean and standard deviation [SD]).

Analyses were conducted for the percentage change from baseline (time-matched day −1) observed at the end of treatment (day 28) in absolute lymphocyte and lymphocyte subset cell counts, VCAM-1 inhibition, and α_4_ expression. These data were analyzed using a mixed-effect model repeated-measures analysis of variance (ANOVA; PROC MIXED in SAS) with the subject as the random effect, treatment and treatment by time-point interaction as fixed effects, and baseline values as a covariate. The treatment by time-point interaction was inspected. The dose-response relationship (using the dosages of 0, 200 mg/day, 1000 mg/day [as 1000 mg qd or 500 mg bid], or 2000 mg/day) was investigated by means of a linear trend test contrast [20] for unequally spaced doses in the SAS PROC MIXED model with a type-I error of 0.05. (Negative quadratic contrast was added to check for any plateau effect.).

In addition, five pairwise comparisons to the placebo group were evaluated (with the higher dosages emphasized: 1000 mg bid, 1000 mg qd, and 500 mg bid), and the 500-mg bid and 1000-mg qd regimens were also compared (to assess any potential regimen effect). These comparisons were applied using SAS contrasts of the PROC MIXED procedure. To take into account multiple comparisons (five pairwise comparisons), the type I error of 0.05 was adjusted by Bonferroni correction for these pairwise comparisons. Thus, differences were considered statistically significant when *P*<0.01.

### Ethics Statement

The study was approved by the London–Surrey Borders Research Ethics Committee and overseen by an independent safety advisory board (see Acknowledgments for board members). It was conducted in accordance with the ethical principles of the Declaration of Helsinki, the International Conference on Harmonisation Good Clinical Practice guidelines, and UK laws. Written consent was obtained from all subjects. The study was registered at the National Institutes of Health ClinicalTrials.gov website (identifier: NCT00726648).

## Results

### Subject Baseline Characteristics

**Figure 1:**
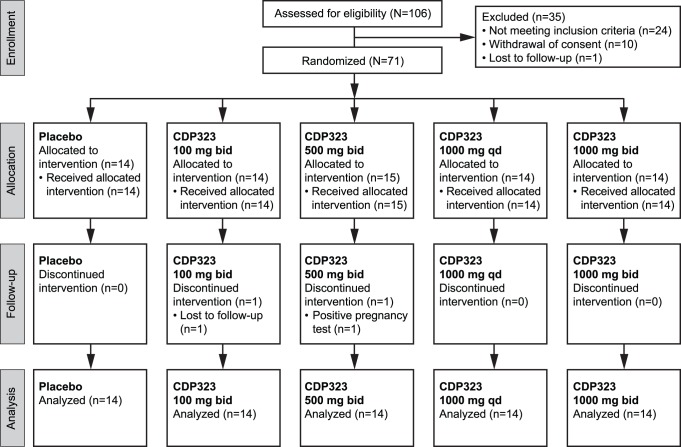
Subject disposition.

A total of 106 subjects were screened, of whom 71 were randomized to treatment. The majority of subjects (97.2%; n = 69) completed the study; one subject in the CDP323 100-mg bid group was lost to follow-up and one subject in the CDP323 500-mg bid group discontinued because of a positive pregnancy test at day 27. The latter subject, who did not receive study drug on day 28, was the only exclusion from the PP population (n = 70). Subject disposition in each of the treatment groups is given in Figure 1.

Baseline demographics and disease characteristics are presented in Table 1. There were no clinically relevant differences between the treatment groups with respect to age, race, body mass index, or EDSS score. Overall, 62% of the subjects were female, although the percentage of female subjects in each group varied from 43% to 80%.

**Table 1:** Subject baseline characteristics.

Characteristic	Placebo (n = 14)	CDP323 100 mg bid (n = 14)	CDP323 500 mg bid (n = 15)	CDP323 1000 mg qd (n = 14)	CDP323 1000 mg bid (n = 14)	Overall (N = 71)
Age, mean, y (range)	45.5 (25.5–60.1)	46.4 (36.3–62.3)	46.0 (27.4–59.0)	45.4 (24.8–59.7)	47.2 (41.3–53.9)	46.1 (24.8–62.3)
Female, n (%)	6 (42.9)	7 (50.0)	12 (80.0)	8 (57.1)	11 (78.6)	44 (62.0)
Caucasian, n (%)	13 (92.9)	14 (100)	13 (86.7)	12 (85.7)	14 (100)	66 (93.0)
BMI, mean (range)	27.7 (18.1–38.0)	28.4 (21.2–43.3)	28.1 (21.6–39.9)	27.9 (20.4–45.2)	28.5 (18.3–45.0)	28.1 (18.1–45.2)
Creatinine clearance, mean,mL/min (range)^a^	134.1 (79–212)	114.5 (79–227)	113.9 (86–192)	126.6 (78–222)	121.9 (75–231)	122.2 (75–231)
Type of MS, n (%)						
Relapsing remitting	11 (78.6)	10 (71.4)	11 (73.3)	8 (57.1)	10 (71.4)	50 (70.4)
Secondary progressive	3 (21.4)	4 (28.6)	4 (26.7)	6 (42.9)	4 (28.6)	21 (29.6)
EDSS						
Mean (SD)	5.5 (1.5)	4.3 (1.7)	4.9 (1.6)	4.8 (1.8)	5.2 (1.7)	4.9 (1.7)
Median (range)	6.0 (1.5–6.5)	4.8 (2.0–6.5)	6.0 (2.0–6.5)	5.8 (2.0–6.5)	6.0 (1.0–6.5)	6.0 (1.0–6.5)
Relapses in last 12 months						
Mean (SD)	1.6 (1.9)	1.1 (0.9)	1.1 (0.8)	1.6 (1.0)	1.1 (0.9)	1.3 (1.2)
Median (range)^b^	1.0 (0–8)	1.0 (0–3)	1.0 (0–3)	1.0 (0–3)	1.0 (0–3)	1.0 (0–8)

a Determined by Cockroft formula: males = [(140 − age)×body weight]/[72×serum creatinine (mg/dL)]; females = [(140 − age)×body weight]/[72×serum creatinine (mg/dL)]×0.85.

b Although 12 subjects had not experienced a relapse in the previous 12 months (4 in the 1000-mg bid group, 3 in each of the 100-mg bid and 500-mg bid groups, and 1 in each of the 1000-mg qd and placebo groups), they all fulfilled the inclusion criteria of having had at least one relapse in the previous 24 months.

bid = twice daily; BMI = body mass index; EDSS = Expanded Disability Status Scale; qd = once daily; SD = standard deviation.

The majority of subjects had a diagnosis of relapsing-remitting MS; a higher percentage of subjects had a diagnosis of relapsing secondary progressive MS in the CDP323 1000-mg qd group (43%) than in the other groups (21% to 29%). The mean annualized relapse rate determined over the 12 months prior to the study was 1.3.

### PD Effects of CDP323

Summary data for absolute counts of lymphocytes and lymphocyte subsets at baseline, day 1, and day 28 are presented in Table 2. Table 3 presents the percentage change from baseline, based on absolute counts, in total peripheral blood lymphocytes and lymphocyte subsets following treatment with CDP323 for 28 days.

**Table 2:** Summary statistics on absolute counts of lymphocytes subsets at baseline, day 1, and day 28 after treatment.

Lymphocyte markers, mean (SD),10^3^ cells/µL^a^	Day	Placebo (n = 14)	CDP323 100 mg bid (n = 14)	CDP323 500 mg bid (n = 14)	CDP323 1000mg qd (n = 14)	CDP323 1000 mg bid (n = 14)`
Total lymphocytes (CD45+)	Day −1 (baseline)	2.756 (1.246)	2.353 (0.587)	2.421 (0.619)	2.758 (0.779)	2.344 (0.449)
	Day 1	2.564 (0.841)	2.635 (0.597)	3.109 (0.754)	3.510 (0.985)	3.316 (0.897)
	Day 28	2.629 (0.812)	2.570 (0.656)	3.409 (0.981)	3.512 (1.155)	3.455 (0.802)
Naive B cells (CD19+/CD20+)	Day −1 (baseline)	0.444 (0.324)	0.291 (0.146)	0.319 (0.133)	0.313 (0.125)	0.285 (0.087)
	Day 1	0.422 (0.234)	0.354 (0.138)	0.448 (0.167)	0.478 (0.177)	0.496 (0.162)
	Day 28	0.415 (0.286)	0.347 (0.198)	0.551 (0.286)	0.522 (0.245)	0.557 (0.245)
Memory B cells	Day −1 (baseline)	0.097 (0.064)	0.067 (0.026)	0.086 (0.051)	0.092 (0.065)	0.083 (0.059)
(CD19+/CD20+/CD27+)	Day 1	0.088 (0.046)	0.089 (0.035)	0.149 (0.096)	0.147 (0.095)	0.160 (0.100)
	Day 28	0.090 (0.046)	0.083 (0.039)	0.190 (0.132)	0.182 (0.150)	0.194 (0.114)
T cells (CD3+)	Day −1 (baseline)	1.934 (0.872)	1.732 (0.411)	1.692 (0.524)	2.088 (0.618)	1.689 (0.332)
	Day 1	1.769 (0.621)	1.841 (0.434)	2.137 (0.629)	2.527 (0.798)	2.282 (0.654)
	Day 28	1.874 (0.553)	1.860 (0.407)	2.310 (0.665)	2.537 (0.841)	2.363 (0.494)
NK T cells (CD3+/CD56+)	Day −1 (baseline)	0.075 (0.068)	0.069 (0.053)	0.068 (0.074)	0.078 (0.063)	0.065 (0.039)
	Day 1	0.077 (0.077)	0.089 (0.095)	0.097 (0.103)	0.104 (0.092)	0.096 (0.069)
	Day 28	0.089 (0.076)	0.073 (0.062)	0.084 (0.081)	0.107 (0.108)	0.080 (0.051)
NK cells (CD3−/CD56+)	Day −1 (baseline)	0.208 (0.101)	0.197 (0.099)	0.248 (0.197)	0.215 (0.137)	0.200 (0.082)
	Day 1	0.222 (0.111)	0.290 (0.136)	0.327 (0.262)	0.286 (0.157)	0.314 (0.149)
	Day 28	0.195 (0.065)	0.229 (0.109)	0.333 (0.303)	0.272 (0.169)	0.300 (0.103)
Monocytes (CD14+)	Day −1 (baseline)	0.390 (0.128)	0.295 (0.092)	0.329 (0.147)	0.321 (0.071)	0.391 (0.137)
	Day 1	0.348 (0.157)	0.323 (0.100)	0.391 (0.176)	0.375 (0.114)	0.437 (0.166)
	Day 28	0.369 (0.060)	0.322 (0.103)	0.404 (0.203)	0.412 (0.237)	0.432 (0.131)
Hematopoietic progenitor cells	Day −1 (baseline)	0.003 (0.001)	0.003 (0.002)	0.003 (0.002)	0.003 (0.001)	0.003 (0.001)
(CD34+)	Day 1	0.003 (0.001)	0.004 (0.002)	0.006 (0.003)	0.006 (0.003)	0.006 (0.002)
	Day 28	0.003 (0.001)	0.004 (0.003)	0.007 (0.003)	0.005 (0.001)	0.006 (0.002)

a Mean value over 24 hours.

bid = twice a day; NK = natural killer; qd = once daily; SD = standard deviation.

**Table 3:** Percentage change from baseline in absolute lymphocyte counts at day 28 following treatment with CDP323 for 28 days (per protocol population).

Lymphocyte type	Treatment group^a^	Mean change from baseline, %^b^	Mean change vs placebo,% (99% CI)^c^	*P* value
Total lymphocytes (CD45+)	Linear trend test			**<0.0001**
	Quadratic trend test			0.0669
	Placebo	12.1	–	–
	CDP323 100 mg bid	8.1	−4.0 (−28.7, 20.6)	0.6678
	CDP323 500 mg bid	43.7	31.6 (6.9, 56.3)	**0.0012**
	CDP323 1000 mg qd	39.7	27.6 (2.9, 52.3)	**0.0042**
	CDP323 1000 mg bid	46.2	34.2 (9.5, 58.8)	**0.0005**
Naive B cells (CD19+/CD20+)	Linear trend test			**<0.0001**
	Quadratic trend test			0.7857
	Placebo	33.2	*–*	*–*
	CDP323 100 mg bid	13.0	−20.3 (−78.9, 38.4)	0.3637
	CDP323 500 mg bid	74.8	41.6 (−17.1, 100.3)	0.0650
	CDP323 1000 mg qd	69.4	36.1 (−22.8, 95.0)	0.1091
	CDP323 1000 mg bid	101.8	68.6 (9.9, 127.2)	**0.0028**
Memory B cells	Linear trend test			**<0.0001**
(CD19+/CD20+/CD27+)	Quadratic trend test			0.1080
	Placebo	31.6	*–*	*–*
	CDP323 100 mg bid	18.2	−13.4 (−87.1, 60.3)	0.6343
	CDP323 500 mg bid	138.0	106.4 (33.2, 179.7)	**0.0002**
	CDP323 1000 mg qd	126.3	94.7 (21.3, 168.2)	**0.0010**
	CDP323 1000 mg bid	166.8	135.2 (62.3, 208.1)	**<0.0001**
T cells (CD3+)	Linear trend test			**0.0019**
	Quadratic trend test			0.0542
	Placebo	15.5	–	–
	CDP323 100 mg bid	9.1	−6.4 (−33.1, 20.3)	0.5277
	CDP323 500 mg bid	41.4	25.9 (−0.9, 52.7)	0.0128
	CDP323 1000 mg qd	41.1	25.6 (−1.3, 52.4)	0.0140
	CDP323 1000 mg bid	38.4	22.9 (−3.8, 49.5)	0.0261
NK T cells (CD3+/CD56+)	Linear trend test			0.0900
	Quadratic trend test			**0.0146**
	Placebo	13.3	–	–
	CDP323 100 mg bid	14.8	1.5 (−31.3, 34.3)	0.9043
	CDP323 500 mg bid	50.1	36.8 (4.1, 69.5)	**0.0039**
	CDP323 1000 mg qd	36.6	23.2 (−10.0, 56.5)	0.0693
	CDP323 1000 mg bid	28.8	15.5 (−17.3, 48.3)	0.2171
NK cells (CD3+/CD56+)	Linear trend test			**<0.0001**
	Quadratic trend test			0.0836
	Placebo	5.5	–	–
	CDP323 100 mg bid	23.5	18.0 (−9.0, 45.1)	0.0836
	CDP323 500 mg bid	47.6	42.1 (14.9, 69.2)	**<0.0001**
	CDP323 1000 mg qd	40.5	35.0 (7.8, 62.2)	**0.0010**
	CDP323 1000 mg bid	57.7	52.2 (25.2, 79.2)	**<0.0001**
Monocytes (CD14+)	Linear trend test			0.0991
	Quadratic trend test			0.8544
	Placebo	21.5	–	–
	CDP323 100 mg bid	8.0	−13.5 (−54.3, 27.4)	NR
	CDP323 500 mg bid	27.6	6.1 (−34.6, 46.8)	NR
	CDP323 1000 mg qd	27.9	6.4 (−34.6, 47.3)	NR
	CDP323 1000 mg bid	37.2	15.7 (−24.7, 56.1)	NR
Hematopoietic	Linear trend test			0.0592
progenitor cells (CD34+)	Quadratic trend test			0.2727
	Placebo	18.6	–	–
	CDP323 100 mg bid	65.1	46.5 (−126.7, 219.6)	NR
	CDP323 500 mg bid	185.7	167.1 (−11.5, 345.6)	NR
	CDP323 1000 mg qd	75.4	56.8 (−110.6, 224.2)	NR
	CDP323 1000 mg bid	140.4	121.8 (−39.7, 283.2)	NR

a n* = *14 per group except for CD34+ cells, where n = 5 for placebo and CDP323 1000 mg bid and n = 4 for CDP323 100 mg bid, CDP323 500 mg bid, and CDP323 1000 mg qd.

b Least squares mean from analysis of variance (ANOVA).

c Statistical comparisons were made using univariate ANOVA.

bid = twice daily; CI = confidence interval; NK = natural killer; NR = not reported (linear trend test for dose-response relationship was not statistically significant); qd = once daily.

Note: For all lymphocyte types, differences between the 500-mg bid and 1000-mg qd dosages were not statistically significant and are not displayed in this table.

Dose-response analysis showed a statistically significant linear trend increase for total lymphocytes, naive B cells, memory B cells, T cells, and NK cells. For NK T cells, the significant negative quadratic trend highlighted a rise of the NK T cell count from 0 mg (placebo) to 1000 mg/day (500 mg bid and 1000 mg qd), corresponding to the daily dose at which maximum increase occurred (mean of 43.4%), followed by a decline (28.8%) at 2000 mg/day.

Mean increases in the total lymphocyte count of 43.7% and 46.2% were found with CDP323 dosages of 500 mg bid and 1000 mg bid, respectively. Smaller mean increases were seen with the 1000-mg qd (39.7%) and 100-mg bid (8.1%) dosages as well as in the placebo group (12.1%). Increases in total lymphocytes were statistically significantly greater with all dosages of CDP323 except the lowest (100-mg bid) dosage than with placebo (Table 2). On day 28, the peak percentage of total lymphocytes was generally observed 8 to 12 hours following dosing of CDP323, and it returned to pretreatment baseline values approximately 24 hours after dosing.

Compared with placebo, the 500-mg bid, 1000-mg qd, and 1000-mg bid dosages of CDP323 generally resulted in statistically significant increases in naive B, memory B, and NK cells (Table 3). Increases in NK T cells observed with CDP323 compared with placebo reached statistical significance only with the 500-mg bid dosage. Although the mean percentage increase from baseline in hematopoietic progenitor cells was substantially greater following administration of CDP323 (65.1%–185.7%) compared with placebo (18.6%), the considerable intersubject variability occluded any statistically significant findings for the 100-mg bid and 1000-mg bid dosages. With the exception of the 100-mg bid dosage, CDP323 resulted in increases in monocytes compared with placebo, although none was statistically significant. Similarly, no statistically significant differences were observed for any of the lymphocyte subsets with CDP323 100 mg bid compared with placebo. Results for changes in T_reg_ cells and inflammatory T cells were inconclusive because the number of samples was insufficient for meaningful analysis.

**Figure 2:**
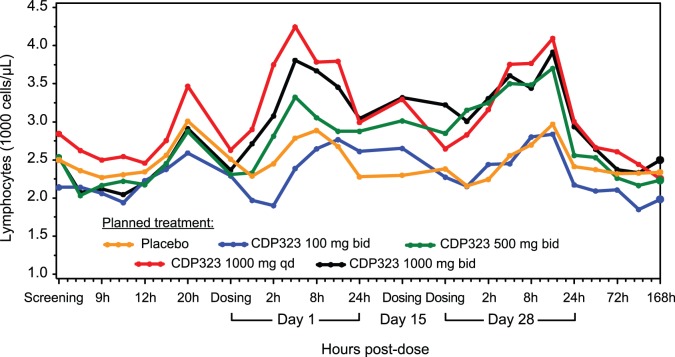
Geometric mean time profile for total lymphocyte count by administered treatment (per protocol population). bid = twice daily; qd = once daily.

In general, each of the PD parameters assessed returned to predose values within 24 to 96 hours after the last dose of CDP323 on day 28. Figure 2 shows the time profile of geometric mean values for total lymphocytes from all dosage groups.

**Figure 3:**
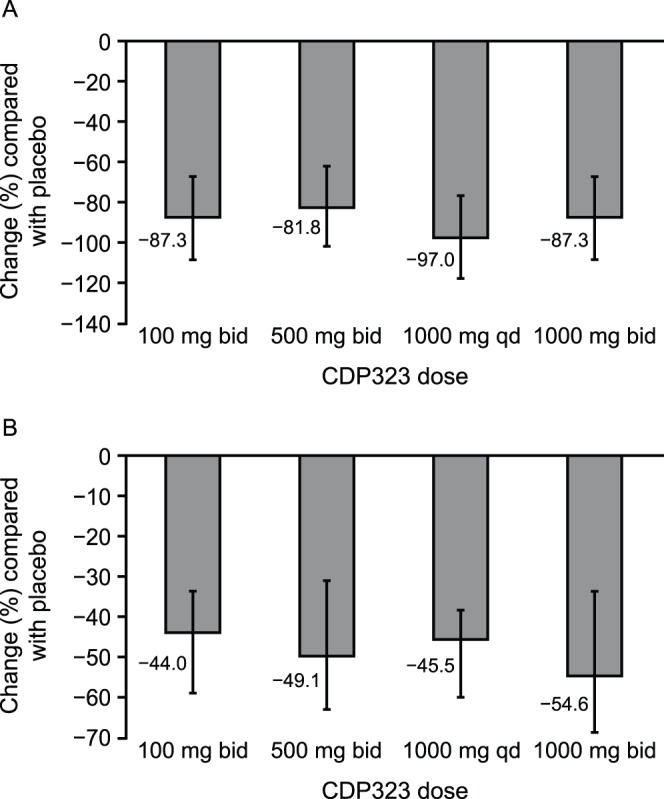
Mean change (95% CI) in (A) lymphocyte VCAM-1 binding and (B) VCAM-1–binding cell α_4_ integrin expression following CDP323 administration. bid = twice daily; CI = confidence interval; qd = once daily; VCAM-1 = vascular adhesion molecule-1.

Patients in the placebo group showed mean increases in lymphocyte binding to VCAM-1 of 41.7% and expression of α_4_-integrin on VCAM-1–binding cells of 33.6%, which may be the result of normal variations observed in lymphocyte counts [21]. In contrast, administration of CDP323 (at all dosages) caused statistically significant reductions compared with placebo in lymphocyte binding to VCAM-1 (mean reduction of 81.8%–97.0%; *P*≤0.003; Figure 3A) and in the expression of α_4_-integrin on VCAM-1–binding cells (mean reduction of 44.0%–54.6%; *P*≤0.0001; Figure 3B).

CDP323 was well tolerated in this study, with headache being the most frequently reported adverse event (AE) across the treatment groups (Table 4). The majority of AEs were mild or moderate in severity, and there were no deaths during the study. Four subjects reported a total of five serious AEs: hypoglycemia (placebo), unintended pregnancy and abortion induced (CDP323 500 mg bid), gamma-glutamyltransferase increase (CDP323 1000 mg bid), and hallucinations mixed (CDP323 1000 mg qd). Only the pregnancy event resulted in discontinuation from the study. Four subjects treated with CDP323 had at least one elevated liver function test of grade 3 or 4 according to the Common Terminology Criteria for Adverse Events (version 3; 9 August 2006). There were no consistent changes in any safety laboratory or vital signs parameters. No cases of opportunistic infection were identified during the study.

**Table 4:** Summary of adverse events reported by ≥2 subjects in any treatment group (safety population).

Adverse event, n (%)	Placebo(n = 14)	CDP323 100 mg bid (n = 14)	CDP323 500 mg bid (n = 15)	CDP323 1000 mg qd (n = 14)	CDP323 1000 mg bid (n = 14)
Subjects with at least one AE	13 (92.9)	7 (50.0)	13 (86.7)	10 (71.4)	13 (92.9)
Headache	3 (21.4)	2 (14.3)	0	3 (21.4)	5 (35.7)
Fatigue	3 (21.4)	0	3 (20.0)	1 (7.1)	4 (28.6)
Diarrhea	3 (21.4)	0	2 (13.3)	1 (7.1)	0
Hypoasthesia	1 (7.1)	0	1 (6.7)	1 (7.1)	2 (14.3)
Muscle spasms	1 (7.1)	1 (7.1)	1 (6.7)	0	2 (14.3)
Vomiting	2 (14.3)	0	1 (6.7)	0	2 (14.3)
Back pain	0	0	1 (6.7)	2 (14.3)	1 (7.1)
Palpitations	0	3 (21.4)	1 (6.7)	0	0
Paresthesia	2 (14.3)	0	2 (13.3)	0	0
Muscular weakness	0	0	0	1 (7.1)	2 (14.3)
Dysphonia	0	0	0	2 (14.3)	0
Fall	0	0	0	0	2 (14.3)
Herpes simplex	0	0	2 (13.3)	0	0
Pain in extremity	0	0	2 (13.3)	0	0
Pharyngolarungeal pain	0	0	0	0	2 (14.3)

AE = adverse event; bid = twice daily; qd = once daily.

## Discussion

This hybrid phase 1/2 study assessed the effect of different dosages of CDP323, a small-molecule α_4_-integrin antagonist, on changes in total lymphocytes and lymphocyte subsets in subjects with RMS. In general, the numbers of circulating T, B, NK, NK T, and hematopoietic progenitor cells were significantly greater in subjects who had received CDP323 dosages of 1000 mg qd (smallest increases), 500 mg bid, or 1000 mg bid (largest increases) than in those who had received placebo. In total lymphocytes and most subtypes, the dose-response relationship was shown to be linear.

The observed effect of CDP323 on the total peripheral blood lymphocyte count and the number of circulating mature and immature B cells was similar to the effects previously reported with the humanized monoclonal α_4_β_1_-integrin antibody natalizumab [22]–[24]. Unlike natalizumab, CDP323 had no significant effect on the number of circulating monocytes [23], nor on T_reg_ and inflammatory T-cell counts. However, the sample size for the T_reg_ and inflammatory T-cell results was too small to permit any definitive conclusions to be drawn.

The PD effects of CDP323 on lymphocytes and lymphocyte subsets are readily reversible, with cell numbers generally returning to predose levels within 96 hours after the last administered dose. However, given the short duration of this study, it is not possible to predict treatment reversibility after long-term CDP323 administration.

Functional inactivation of the α_4_ integrin has been observed following mobilization of CD34+ hematopoietic stem cells [25], and it is known that these cells are mobilized by natalizumab when it is used in the treatment of patients with MS [26]–[29]. CDP323 appears to have similar effects on these cells and was observed to significantly decrease the capacity of lymphocytes to bind VCAM-1 and α_4_ integrin on VCAM-1–binding cells compared with placebo.

The differences in the effects of CDP323 and natalizumab on circulating lymphocytes demonstrate that despite targeting the same molecule, albeit via different delivery pathways, these compounds may induce distinct changes in leukocyte production and, possibly, functionality. Though the significance of these findings in relation to α_4_ antagonism and changes in circulating lymphocyte levels remain unknown, the limited efficacy of CDP323 on brain lesions in a serial MRI phase 2 study [19] as compared with the substantial efficacy of natalizumab may indicate that the stable antagonism achieved by a long-acting antibody has clinical significance.

## Supporting Information

Checklist S1 **CONSORT Checklist.**
(DOC)

Protocol S1 **Trial Protocol.**
(PDF)
