# Supplementary material for: Pharmacodynamic Consequences of Administration of VLA-4 Antagonist CDP323 to Multiple Sclerosis Subjects: A Randomized, Double-Blind Phase 1/2 Study
Source: PLoS One. 2013 Mar 5;8(3):e58438. doi: 10.1371/journal.pone.0058438 (PMC3589412; doi:10.1371/journal.pone.0058438)
Supplement: Protocol S1 — Trial Protocol. (PDF) [file pone.0058438.s002.pdf]

Protocol S1

1. TITLE

Protocol of Study C32325

Double-blind, placebo-controlled, randomized, parallel-group study in subjects with relapsing forms of multiple sclerosis to evaluate the effects of different CDP323 doses on biomarker patterns as well as on safety and tolerability.

.

UCB Pharma S.A.
R&D
Chemin du Foriest
B-1420 Braine-l'Alleud
BELGIUM
with Registered Office at
Allée de la Recherche 60
B-1070 Brussels
BELGIUM

Hereafter "UCB"

CDP323
EudraCT Number 2008-000147-34
+ IND Number 74863

Confidential Material
The information contained in this document is strictly confidential. It is disclosed to you as a (potential) Investigator or (potential) consultant. Acceptance of this document constitutes your agreement that the information contained herein will not be disclosed or in any way communicated to any third party without prior written approval of UCB (or it's representative(s)) of the clinical study described therein except – to the extent necessary – to your staff, to members of appropriate review committee(s) and/or to persons to whom the investigational product may be administered or to their legal representative(s) to obtain their informed consent.

Authorized signature on behalf of UCB:

UCB Clinical Program Director

_________________________ ________
Signature Date
Name: Dr Christian Wolf MD MPPM

Principal Investigator
By my signature below, I acknowledge that I have read the protocol (RPCE07L2605) and agree that it contains all necessary details for carrying out the clinical study described therein. Furthermore, I agree to conduct this clinical study in compliance with said Protocol, the ICH Good Clinical Practice guideline, as well as with any and all applicable federal, state and/or local laws and regulations and with my contractual obligations towards UCB or its representatives(s).

Signature: ________________________________ Date: ____________

Printed Name: ____

Address: ____

____

Phone: ______________________ Site Number: ____________

2. CONTACT INFORMATION

Sponsor
UCB Pharma S.A.
Chemin du Foriest
B-1420 Braine-l'Alleud (Belgium)

UCB Contributors:

Study Physician (Study related questions)

Name:	Dr Bella Ertik MD	Phone:	+32 2 386 35 64
		Mobile:	+32 494 578 287
		Fax:	+32 2 386 27 28

Clinical trial Manager (CTM)/Clinical Project Manager (CPM)

Name:	Ans Valgaeren	Phone:	+32 2 386 35 03
		Fax:	+32 2 386 35 37

Pharmacokineticist

Name:	Dirk Garmann	Phone:	+49 2173 48 2591
		Fax:	+49 2173 48 1879

Trial Statistician

Name:	Christian Otoul	Phone:	+32 2 386 2504
		Fax:	+32 2 386 3558

SAEs Reporting (24h/24), Safety Related Issues and Emergency Unblinding

- **Fax**: +32 2 386 24 21
- **Phone**: +32 2 386 24 68

Investigators
The complete and updated list of investigators is maintained in the Trial Master File (TMF) at UCB.

Signatory Coordinating Investigator
Dr. Jörg Taubel MD MFPM
Richmond Pharmacology Ltd
St greaorge's University of London
Cranmer Terrace, Tooting
London SW17ORE
Phone: +44 (0)20 8664 5200/Fax: +44 (0)20 8664 5201

3. TABLE OF CONTENTS

1. TITLE **1**

2. CONTACT INFORMATION **3**

3. TABLE OF CONTENTS **4**

4. LIST OF ABBREVIATIONS **8**

5. PROTOCOL SUMMARY **11**
5.1 Study Schedule of Assessments 19

6. BACKGROUND INFORMATION **22**
6.1 Indication to be Studied and Medical Need 22
6.2 The Investigational Product CDP323 24
6.2.1 Pharmacology 24
6.2.2 Metabolism and Pharmacokinetics 24
6.2.3 Toxicology 25
6.2.4 Safety Pharmacology 26
6.2.5 Pharmacodynamics 27
6.2.6 Clinical Profile 27
6.2.7 Known and Potential Risks and Benefits to Human Subjects 28
6.3 Justification of Study Design, Doses Selected, and Population 30
6.3.1 Rationale for Study Design 30
6.3.2 Rationale for Doses Selected 30
6.3.3 Rationale for the Population Selected 32

7. STUDY OBJECTIVES **32**
7.1 Primary Objective 32
7.2 Secondary Objective 32
7.3 Exploratory Objective 32

8. STUDY DESIGN **32**
8.1 Type/Design 32
8.2 General Design Considerations 34
8.3 Subjects/Sites Numbers 35
8.4 Measures to Minimize/Avoid Bias 35
8.4.1 Blinding 35
8.4.2 Randomization 36
8.4.3 Subject Identifier 36
8.5 Study Duration 37
8.6 End of Study 37

9. SELECTION AND WITHDRAWAL OF SUBJECTS **37**
9.1 Subject Inclusion Criteria 37

9.2 Subject Exclusion Criteria 38
9.3 Subject Withdrawal 40

10. TREATMENT OF SUBJECTS (INVESTIGATIONAL PRODUCT AND CONCOMITANT MEDICATIONS) 41
10.1 Study Investigational Products 41
10.1.1 Description of all Investigational Products 41
10.1.2 Packaging 41
10.1.3 Labeling 42
10.1.4 Storage Requirements 43
10.1.5 Monitoring of Subject Compliance 43
10.1.6 Investigational Products Accountability 43
10.2 Concomitant Treatments and Rescue Medications 44
10.2.1 Rescue Medication 44
10.2.2 Permitted Concomitant Treatments (Medications and Therapies) 44
10.2.3 Prohibited Concomitant Treatments (Medications and Therapies) 45

11. STUDY PROCEDURES 46
11.1.1 Informed Consent 46
11.1.2 Demographic Data 46
11.1.3 General Medical and Procedures History 46
11.1.4 MS History and Diagnosis 46
11.1.5 MS Medication History 46
11.1.6 Vital Signs 47
11.1.7 Physical Examination 47
11.1.8 Childbearing Potential and Birth Control 47
11.1.9 Laboratory 48
11.1.10 Electrocardiogram (ECG) 50
11.1.11 Magnetic Resonance Imaging (MRI) 51
11.1.12 Pharmacokinetic and Pharmacodynamic Sampling 52
11.1.13 Neuro Exam and EDSS 53
11.1.14 Assessment of New Neurological Events 54
11.1.15 Surveillance for PML and Diagnostic Work-Up 55
11.1.16 Concurrent Medical Procedures 56
11.1.17 Concomitant Medication 57
11.1.18 Adverse Events (AEs) 57
11.1.19 Drug Administration 57
11.1.20 Confinement 58
11.1.21 End of Treatment Phase and End of Drug-free Follow-up 58
11.2 Description Visit by Visit 58
11.2.1 Visit 1 (Day -21) Screening 58
11.2.2 Visit 2 (Day -14) Screening 59
11.2.3 Visit 3 60

11.2.4 Visit 4 (Day 8) Treatment Period 61
11.2.5 Visit 5 (Day 15) Treatment Period 62
11.2.6 Visit 6 62
11.2.7 Visit 7 (Day 30) 63
11.2.8 Visit 8 (Day 31) 63
11.2.9 Visit 9 (Day 32) 64
11.2.10 Visit 10 (Day 35) 64
11.2.11 Visit 11 (+3 months) Follow-up 64
11.2.12 Visit 12 (+12 months) Follow-up 64
11.2.13 Unscheduled Contact with the Study Site 65

12. ADVERSE EVENTS AND SERIOUS ADVERSE EVENTS 65
12.1 Adverse Events 65
12.1.1 Definition of Adverse Event (AE) 65
12.1.2 Procedures for Reporting and Recording Adverse Events 66
12.1.3 Description of AEs 66
12.1.4 Follow-up on Adverse Events 69
12.1.5 Rule for Repetition of an AE 69
12.1.6 Pregnancy 69
12.1.7 Overdose of Investigational Product 70
12.2 Serious Adverse Events 70
12.2.1 Definition of Serious Adverse Event (SAE) 70
12.2.2 Procedures for Reporting Serious Adverse Events (SAEs) 71
12.2.3 Follow-up of Serious Adverse Events 72

13. STATISTICS 72
13.1 Statistical and Analytical Plans 72
13.1.1 Study Population(s) 72
13.1.2 Safety and Other Variables 73
13.1.2.1 Pharmacokinetic Variables 73
13.1.2.2 Pharmacodynamic (Biomarkers) Variables 74
13.1.2.3 Safety Variables 75
13.1.3 Statistical Evaluation 75
13.1.4 Pharmacokinetic and Pharmacodynamic (Biomarkers) Variable Analysis 76
13.1.5 Safety Variable Analyses 77
13.2 Determination of the Sample Size 77
13.3 Changes in the Conduct of the Study or the Planned Analyses 77
13.4 Statistical and Analytical Issues 77
13.4.1 Adjustments for Covariates and Interactions 77
13.4.2 Handling of Dropouts or Missing Data 77
13.4.3 Interim Analysis 77
13.4.4 Examination of Subgroups 78
13.4.5 Use of Rescue Medication 78

13.5 Pre-Analysis Review of Data 78
13.6 Criteria for Unblinding the Results 78
13.7 Dictionaries 78

14. ETHICS AND REGULATORY REQUIREMENTS 79
14.1 Approval 79
14.2 Subject Information and Consent 80
14.3 Subject Confidentiality 81
14.4 Informing the General Practitioner 81

15. STUDY MANAGEMENT AND ADMINISTRATION 81
15.1 Monitoring 81
15.2 Direct Access to Source Data/Documents 82
15.3 Audit and Inspection 83
15.4 Case Report Forms (CRF) and Screen Failures 83
15.5 Adherence to Protocol 84
15.6 Investigator Site File 84
15.7 Data Handling 85
15.8 Termination of Study 85
15.9 Clinical Study Report 85
15.10 Insurance and Liability 86
15.11 Archiving and Data Retention 86
15.12 Allocation of Responsibilities 86
15.13 Curriculum Vitae (CV) 87
15.14 Financial Disclosure 87
15.15 Good Clinical Practice 87
15.16 Disclosure of Clinical Trial Information 87
15.17 Publication 87

16. REFERENCES 89

17. APPENDICES 91
17.1 Expanded Disability Status Scale (EDSS) 91
17.2 Mc Donald Diagnostic Criteria for MS 121
17.3 Common Terminology Criteria for Adverse Events (CTCAE) 123

List of Tables

Table 8:1 Dosing groups 33
Table 10:1 Dosing schedule 42
Table 11:1 Overview about the Blood Sampling Schedule Including Volumes Per Visit (in mL) 49
Table 11:2 PK/PD Sampling Schedule 53
Table 11:3 Differential Diagnosis between MS and PML: Clinical Considerations 55

4. LIST OF ABBREVIATIONS

ACTH	Adrenocorticotropic hormone
AE	Adverse Event
ALAT or SGPT	Alanine aminotransferase
AM1	Active Metabolite 1 (CT7758)
AM2	Active Metabolite 2 (CT533652-00)
AP	Alkaline phosphatase
ASAT OR SGOT	Aspartate aminotransferase
AUC	Area Under the concentration-time Curve from 0 to infinity
AUC (0-t)	Area Under the concentration-time Curve from 0 to the time of the last quantifiable concentration
AUC(0-12)	Area Under the concentration-time Curve from 0 to 12 h
AUC_{SS}	Area Under the concentration-time Curve at steady state
AUCτ	Area Under the concentration-time Curve over a dosing interval
$AUC_{\tau,ss}$	Area Under the concentration-time Curve during a dosing interval at steady state
BOD	Burden of disease
CDMS	Clinical Data Management System
CHMP	Committee for Medicinal Products for Human Use
CI	Confidence Interval
CIS	Clinically Isolated Syndrome
CL/F	Apparent total body clearance
C_{max}	Maximum concentration
C_{max}	Maximum concentration at steady state
CNS	Central Nervous System
CPMP	Committee for Proprietary Medicinal Product
Cr Cl	Creatinine clearance
CRF	Case Report Form
CRO	Contract Research Organization
CSF	Cerebrospinal Fluid
CTM	Clinical Trial Manager
CV	Curriculum Vitae
DMT	Disease Modifying Treatment
DSC	Drug Supply Coordinator
DTPA	Di-ethylene triamine pentaacetate
EAE	Experimental Allergic Encephalomyelitis
ECG	Electrocardiogram
EDSS	Expanded Disability Status Scale
EMEA	European Medicines Agencies
Emax	Maximal Effect
EU	European Union

EWP	Efficacy Working Party
FDA	Food and Drug Administration
GCP	Good Clinical Practice
GGT	Gamma-glutamyltranspeptidase
GMP	Good Manufacturing Practice
GP	General Practitioner
HBsAg	Hepatitis B Surface Antigen
HBV-DNA	Hepatitis B Virus - DNA
HCV-RNA	Hepatitis C Virus - RNA
HERG	Health Economics Research Group
HIPAA	Health Insurance Portability & Accountability Act
HIV1	Human Immunodeficiency Virus 1
HIV2	Human Immunodeficiency Virus 2
IC	Informed Consent
ICH	International Conference on Harmonization
ICMJE	International Committee of Medical Journal Editors
IDMC	Independent Data Monitoring Committee
IEC	Independent Ethics Committee
IFNBs	Beta-Interferons
IgG	Immunoglobulin G
ITT	Intention-to-Treat
IvIg	Intravenous Immunoglobulins
JCV	JC Polyoma Virus
λz	First order terminal elimination rate constant
MCH	Mean Corpuscular Hemoglobin
MCHC	Mean Corpuscular Hemoglobin Concentration
MCV	Mean Corpuscular Volume
MD	Medical Doctor
MedDRA	Medical dictionary for regulatory activities
MoA	Mechanism of Action
MPPM	Molar Parts Per Million
MRI	Magnetic Resonance Image
MS	Multiple Sclerosis
NA	Not Applicable
ND	Not Done
NOAELs	Non Observed Adverse Effect Level
PBMC	Peripheral Blood Mononuclear Cell
PD	Pharmacodynamic
PK	Pharmacokinetic
PML	Progressive Multifocal Leukoencephalopathy
PP	Per-Protocol
PPMS	Primary Progressive Multiple Sclerosis
QA	Quality Assurance

QT	QT interval
QTc	QT interval corrected
QTcF	QT interval corrected according to Fridericia's formula
R_{C12h}	Accumulation indices for C_{12h}
R_{cmax}	Accumulation indices for C_{max}
R_{AUC}	Accumulation indices for AUC(0-12)
RMS	Relapsing Multiple Sclerosis
RPMS	Relapsing-Progressive Multiple Sclerosis
RRMS	Relapsing-Remitting Multiple Sclerosis
SAE	Serious Adverse Events
SAP	Statistical Analysis Plan
SC	Subcutaneous
SD	Source Documents
SD	Standard Deviation
SDV	Source Documents Verification
SOC	System Organ Class
SOPs	Standard Operating Procedure
SPC	Summary of Product Characteristics
SPMS	Secondary Progressive Multiple Sclerosis
SS	Steady State
SSC	Study Steering Committee
SUSAR	Suspected Unexpected Serious Adverse Reaction
t½	Terminal elimination half-life
Tb	Tuberculosis
TEAE	Treatment-Emergent Adverse Event
t_{max}	Time of C_{max}
TMF	Trial Master File
TSH	Thryroid-Stimulating Hormone
UN	Unknown
US	United States of America
V	Visit
VCAM-1	Vascular Cellular Adhesion Molecule-1
Vz/F	Apparent Volume of Distribution
WHO	World Health Organization
WK	Week
βHCG	β-Human Chorionic Gonadotropin
γGT	Gamma-Glutamyltranspeptidase

5. PROTOCOL SUMMARY

TITLE OF THE STUDY
Double-blind, placebo-controlled, randomized, parallel-group study in subjects with relapsing forms of multiple sclerosis to evaluate the effects of different CDP323 doses on biomarker patterns as well as on safety and tolerability.

STUDY TYPE/PHASE
Human pharmacology and therapeutic exploratory study (Phase I/II).

STUDY OBJECTIVES

Primary Objective
The primary objective is to compare the effects of different CDP323 doses (100, 500, and 1000 mg *bid* as well as 1000 mg once daily) given over a period of four weeks on biomarkers (leukocyte-related parameters, degree of VCAM-1 inhibition, proportion of α4-receptor expression) in subjects with relapsing forms of multiple sclerosis (RMS).

Secondary Objective
Assess the safety and tolerability of 1000 mg CDP323 in once daily and twice daily administration over the period of four weeks in subjects with RMS.

Exploratory Objective
Assess the exposure-response relationship between CDP323 (and its metabolites) and changes in biomarkers relative to changes observed with placebo.

STUDY VARIABLES

Pharmacokinetic Variables
Standard non-compartmental methods to determine t_{max}, C_{12h}, C_{max}, $t_{½}$, AUC(0–t), AUC, AUC(0-12), AUC_{SS}, C_{maxSS}, C_{12hSS}, V_z/F, λ_z, CL/F, and accumulation indices for $C_{12h}=R_{C12h}$, $C_{max}=R_{cmax}$ and AUC(0-12)=R_{AUC} for CT7758, CT533652-00, and CDP323.

Pharmacodynamic (Biomarkers) Variables
Leukocytes and leukocyte subsets:

- Total and differential white blood cell count
- T-cells (CD3, CD4, CD8), B-cells (CD19, CD20, CD27 subsets), monocytes (CD14), hematopoietic progenitor cells (CD34), NK cells (CD56), NKT cells (CD3, CD56) and T_{reg} cells (CD4+CD25+FoxP3+), together with α4 expression for each subset.
- VCAM-1 inhibition

Safety Variables

Standard and disease-related safety variables:

- Adverse events (AEs) including MS relapses
- Vital sign parameters (heart rate, blood pressure, respiratory rate, body temperature, body weight)
- Blood chemistry (fasting): sodium, potassium, chloride, calcium, magnesium, urea, creatinine, SGOT, SGPT, γGT, AP, uric acid, bilirubin (total, conjugated and unconjugated), total serum protein, albumin, total cholesterol and glucose
- Hematology: erythrocytes, leukocytes, differential leukocyte count, thrombocytes, hemoglobin, hematocrit, mean corpuscular volume (MCV), mean corpuscular hemoglobin (MCH), mean corpuscular hemoglobin concentration (MCHC)
- Thyroid function tests: TSH
- Clotting tests: partial thromboplastin time and prothrombin time
- Urinalysis: pH, protein, glucose, erythrocytes, leukocytes
- 12-lead ECG: centrally measured or calculated variables: heart rate, PQ time, QRS time and QT time in lead II; QTcF (corrected QT time calculated according to Fridericia's formula [(13)]); central overall assessment of normal/abnormal findings with/without clinical relevance.
- cardiac telemetry for 12 hours before and 36 hours following the first intake of study drug.

Class-related

- Presence of potential clinical signs and/or symptoms of PML
- Presence of potential signs of progressive multifocal leukoencephalopathy (PML) in MRI
- Presence of JC virus DNA in serum and/or peripheral blood mononuclear cells (PBMCs)
- Pre-post status for markers for infectious diseases:
    - Tuberculosis
    - Toxoplasmosis

STUDY DESIGN

This is a hybrid Phase I/II study and will be conducted as a double-blind, randomized, placebo-controlled, dose-controlled study.

Male and female subjects with RMS between the ages of 18 and 65 years will be recruited to participate in this study. Subjects must have a score of 6.5 or less on the Expanded Disability Status Scale (EDSS) (see section 17.1) and must be free of other conditions potentially compromising the immune response. Furthermore, subjects must not take any concomitant immunosuppressive or -modulatory medication including registered or putative disease-modifying treatment (DMT) against MS during exposure to CDP323 and within a thirty-day

period after completing study treatment or after withdrawing from study treatment. Females of childbearing potential will be permitted to participate if they are using appropriate contraception (see inclusion criteria for details).

The subjects will be divided in five groups of 14 subjects each. All subjects will take study medication and/or placebo twice a day for a period of 28 consecutive days (Day 1 till Day 28). The last intake will be the morning dose of Day 28.

	**Treatment Period : 4 weeks Visit 3 - 6 Day 1 to 28**	
	**morning**	**evening**
**Group 1**	1000 mg	placebo
**Group 2**	100 mg	100 mg
**Group 3**	500 mg	500 mg
**Group 4**	1000 mg	1000 mg
**Group 5**	placebo	placebo

In order to prudently manage any unexpected untoward events, all subjects will be hospitalized for the first five days (5 nights) during the treatment period. Further hospitalization will also occur from day 27 evening until day 29 morning, during the more intensive PK/PD blood samplings and can be prolonged until day 35 if deemed necessary by the investigator.

PK sampling will occur over 0-24 hr after the first morning dose, at pre-dose on day 15 and over 0-168 hr after the last dose.

Sampling for PD assessments will occur during most PK sampling timepoints. As food has been shown to substantially increase systemic exposures of CDP323 and its metabolites, all dosing will be with or within 30 minutes following a standard meal. During the confinement period, subjects will be instructed to take the study drug 5 min after their meals (which have to be eaten within 30 min). In order to maintain the blind, PD data (including all leukocyte-related parameters from hematological tests) will not be made available to the study center personnel until after study unblinding, with the exception of leukocyte levels above 15000 x 10^9/l or below 500 x 10^9/l and CD4+ counts below 200 x 10^9/l. Subjects who drop out prior to randomization will be replaced; however, there will be no replacement of subjects dropping out later, including subjects suffering from a relapse.

Subjects will be asked to report back to the study center for a safety check 3 and 12 months after administration of the last dose. The 3-month follow-up visit may be carried out by telephone while subjects are expected to be on site for the 12-month follow-up visit.

PLANNED STUDY PERIOD

FPFV: Q2 2008 / LPLV: Q3 2008

STUDY DURATION PER SUBJECT

The experimental portion of the study will last approximately 8 weeks and will include:

- A screening period up to three weeks before the first dose of study medication;
- A treatment period lasting four weeks with PK/PD assessments until one week after last dosing.

Subjects will be asked to report back to the study center for a safety check 3 and 12 months after administration of the last dose.

PLANNED NUMBER OF SUBJECTS AND SITES

Overall, 70 subjects with RMS will participate in study C32325. Subjects will be randomized to five groups, each comprising 14 subjects. The study will be performed under the supervision of the coordinating CRO Richmond Pharmacology Ltd. (London, UK) in 4-5 units having the capability to hospitalize subjects and being staffed and equipped to handle emergency situations.

This study will require advanced recruitment efforts. As the study duration will not allow subjects to benefit from treatment with CDP323, subjects are considered 'symptomatic volunteers' and will be entitled to compensation appropriate for a Phase I study with a similar burden. It is anticipated that the study coordinating center will have to advertise the study extensively with local and/or national MS advocacy groups and local and/or regional MS clinics employing advanced recruitment strategies, including lay media advertisement.

REGIONS AND COUNTRIES

Great Britain.

MAIN INCLUSION AND EXCLUSION CRITERIA

Inclusion Criteria

Written informed consent signed and dated by the subject.

- Female and male subjects aged 18-65 years inclusive at time of informed consent;
- Being of legal capacity and able to understand the nature of the study and its potential risks;
- Diagnosis of MS according to the revised McDonald criteria [1,2] ;
- Relapsing form of MS, i.e., RRMS or SPMS (with superimposed relapses) according to Lublin and Reingold [3] with at least one clinical relapse in the 24 months before screening and documented in the subject's medical records;

- Screening EDSS score of 0-6.5, inclusive;
- Screening MRI must conform with the diagnosis of MS;
- Must be fully immunocompetent with a CD4+ count >500;
- No detectable JC viral DNA in blood (serum and PBMCs) at two pre-scheduled consecutive measurements during the screening period;
- Willing to comply with the study's safety precautions and with the follow-up at 3 and 12 months after the last drug intake;
- Female subjects of childbearing potential must agree to practice one of the following contraception methods:
  - double-barrier contraception (i.e., using a male or female condom or a diaphragm or cervical cap, all in conjunction with spermicide), or
  - any systemic hormonal contraceptives (i.e., 'the pill', all mini pills, implants, patches, depot injections) plus one of the following: diaphragm or cervical cap, male or female condom, all in conjunction with spermicide, or
  - intrauterine devices releasing levonorgestrel (Mirena), or
  - intrauterine devices without levonorgestrel plus one of the following: male or female condom, diaphragm or cervical cap, all in conjunction with spermicide, or
  - monogamous relationship with partner vasectomized since at least 3 months

  Note: Lack of childbearing potential will be considered under these circumstances:
    - post-menopausal for at least two years;
    - hysterectomy or bilateral oophorectomy, ovariectomy, salpingectomy, or tubal ligation;
    - true sexual abstinence;
    - congenital sterility.
- Sexually active males with partners of child bearing potential must use two approved methods of contraception. In addition, sexually active males must use a condom if their female partner is pregnant or breast feeding. Contraception precautions should continue for at least 3 months after the last dose of CPD323.

Exclusion Criteria

- Any disease other than MS that could better explain the subject's signs and symptoms;
- Any conditions that could interfere with the contrast-enhanced MRI, including an estimated glomerular filtration rate (eGFR) <40 ml/min, or with any other evaluation in the study;
- Any clinically significant disease state or findings other than MS, in particular neoplastic disease or organ transplantation;
- Any clinically significant deviation from the pre-defined ranges for laboratory tests;
- Signs of silent infections, including positive tests for HIV1, HIV2, or Hepatitis B or Hepatitis C, toxoplasmosis, or tuberculosis;
- Known hypersensitivity to gadolinium-containing contrast agents,
- History of clinically significant severe AEs to any drug;

- Participation in any clinical drug trial within 30 days prior to screening;
- Pregnancy or lactation;
- History of alcohol or drug abuse within the year before screening;
- Medical, psychiatric or other conditions that compromise the subject's ability to understand the subject information, to give informed consent, to comply with the trial protocol, or to complete the study;
- Any abnormal, clinically significant ECG findings, in particular any marked pre-study prolongation of the QTcF interval >470 ms
- Known hypersensitivity to ingredients of the CDP323 drug formulation
- Any condition possibly interfering with drug absorption;
- Concomitant treatment with:
    - the CYP1A substrates mexiletine, propafenone, theophylline, verapamil or warfarin, or
    - statins (e.g., atorvastatin), protease inhibitors (e.g., ritonavir), systemic triazole antifungals (e.g., ketoconazole) for their interference with transport protein systems or for their strong inhibition of the P450 cytochrome pathway, or
    - drugs known for their potential to substantially interfere with cardiac repolarization: amiodarone, arsenic trioxide, bepredil, budipine, cisapride, chinidine (quinidine), chloroquine, chlorpromazine, clarithromycin, disopyramide, dofetilide, domperidone, droperidol, erythromycin, halofantrine, haloperidol, ibutilide, levomethadyl, mesoridazine, methadone, pentamidine, pimozide, procainamide, sotalol, sparfloxacin, thioridazine;
- Treatment with the following substances prior to screening within the following time frames:
    - at any time: cyclophosphamide; total lymphoid irradiation; anti-lymphocyte monoclonal antibody treatment (e.g., anti-CD4, Campath-1H);
    - 12 months prior to screening: natalizumab, rituximab, or mitoxantrone.
      Note: prior treatment with natalizumab or rituximab must not have been terminated for safety reasons.
    - 6 month prior to screening: azathioprine, cyclosporine A, human antibodies not listed above, or other strong immunosuppressive drugs;
    - up to 30 days prior to screening: any interferons, glatiramer acetate, corticosteroids, ACTH, IvIg, any other immunomodulating or immunosuppressive drugs including recombinant cytokines, any other putative or experimental MS treatment; any inoculation with attenuated live vaccines;
- Any other reason why, in the investigator's opinion, the subject should not participate.

If a subject suffers a relapse during the screening phase, s/he may not be randomized on the planned visit (V3, Day 1). S/he may be randomized 30 days ± 3 days after the end of the last relapse and must go through a new entire screening procedure.

INVESTIGATIONAL PRODUCT(S)

CDP323 drug substance is a micronized powder with a particle size distribution of D95<15 µm. The drug products to be used are white/white opaque hard gelatin capsules with no markings containing a 1:1 powder blend of CDP323 & ProSolv SMCC 90. The strengths to be used are 50 and 250 mg. Placebo hard-gelatine capsules matching the 50 and 250 mg capsules will be provided for each dosage. The list of excipients is provided in the Investigator's Brochure [(7)].

STATISTICAL METHODS

Populations for Analysis

The intention-to-treat population (ITT) will consist of all randomized subjects who took at least one dose of study medication. The per-protocol population (PP) is a subset of the ITT population, consisting of those subjects who had no major protocol deviations affecting the pharmacodynamic or pharmacokinetic variables, as confirmed during a pre-analysis meeting prior to database lock. The PP population with partial or total exclusion of subjects may be different for PK and PD assessments depending on the type and timing of the deviations.

All PK and PD analyses will be performed on the PP population.

The safety population will consist of all subjects who have received at least one dose of study medication. All safety analyses will be performed on the safety population according to actually received treatment.

Pharmacokinetic and Pharmacodynamic (Biomarkers) Variable Analysis

Descriptive statistics will be presented for all PK and PD variables by treatment and visit where appropriate.

For continuous variables, number of observations, mean, SD, coefficient of variation, median, geometric mean, minimum and maximum will be presented. For categorical variables, the number and percent of subjects with that category will be presented.

Exploratory analysis of the PD variables (leukocytes, α4 expression, VCAM-1 binding) will be performed to compare the effects of the different treatments. Analysis of time-profiles of each PD variable and dose-response relationships will be evaluated. Based on data results, placebo-time effect if any, and dose effect will be modeled using different types of models (linear, asymptotic, E_{max}, etc.) to define maximal effect and dose (ED50) needed to reach 50% of the maximal effect.

The following leukocytes and leukocyte subsets will be explored:

- Total and differential white blood cell count;
- T-cells (CD3, CD4, CD8), B-cells (CD19, CD20, CD27 subsets), monocytes (CD14), hematopoietic progenitor cells (CD34), NK cells (CD56), NKT cells (CD3, CD56) and T_{reg} cells (CD4+CD25+FoxP3+), together with α4 expression for each subset.
- VCAM-1- inhibition

Exploratory PK/PD modeling will also be performed using non-linear mixed effects model (NONMEM software).

Safety Variable Analyses

Descriptive statistics will be presented for all safety variables by treatment and visit where appropriate. No statistical comparison between treatments will be performed, however. For continuous variables, number of observations, mean, SD, median, minimum and maximum will be presented. For categorical variables, the number and percent of subjects with that category will be presented.

Tolerability will be assessed by the incidence of adverse events (AEs). Treatment-emergent AEs will be summarized descriptively by system organ class and preferred term. Additional tables will summarize AEs by severity and relationship to study drug as well as separate tables for AEs leading to withdrawal from the study and serious AEs.

Vital signs, ECG (12-lead and cardiac telemetry), laboratory test values, coagulation and thyroid function and physical examination findings, as well as change from baseline values, will be presented descriptively by treatment. Additionally, a categorical analysis will also be performed. The JVC viral status, tuberculosis and toxoplasmosis status, presence of potential clinical signs of PML will be tabulated. Neurological examinations and EDSS scores (by visit and change from screening) will also be provided in tables.

Sample Size Calculation

No formal statistical testing will be performed and hence no formal sample size calculation was carried out for this exploratory study.

5.1 Study Schedule of Assessments

	V1	V2	V3				V4	V5	V6			V7	V8	V9	V10	V11 (a)	V12
	Day -21	Day -14	Day -1	Day 1	Day 2	Day 5	Day 8	Day 15	Day 27	Day 28	Day 29	Day 30	Day 31	Day 32	Day 35	+3 mon.	+12 mon.
Written Informed Consent	x																
Demographic Data	x																
In/Exclusion Criteria	x	x	x														
Withdrawal Criteria		x	x	x	x	x	x	x	x	x(f)							
Medical & Procedures History	x																
MS History & Diagnosis	x																
MS Medication History	x																
Vital Signs	x			x(h)		x	x	x		x(f)		x	x	x	x	x	x(g)
Physical Examination	x			x				x		x(f)						x	x(g)
Height & Weight	x									x(c) (f)							
Childbearing Potential & Birth Control	x		x	x		x	x	x	x								
Pregnancy Test (Females)	x		x						x								
Blood Chemistry, Hematology	x			x			x			x(f)							
Coagulation	x			x						x(f)							
Thyroid Function	x									x(f)							
Urinalysis	x									x(f)							
JC Virus Parameters	x	x								x(f)							

	V1	V2	V3				V4	V5	V6			V7	V8	V9	V10	V11 (a)	V12
	Day -21	Day -14	Day -1	Day 1	Day 2	Day 5	Day 8	Day 15	Day 27	Day 28	Day 29	Day 30	Day 31	Day 32	Day 35	+3 mon.	+12 mon.
Tuberculosis, Toxoplasmosis	x									x(f)							
HIV, Hepatitis	x									x(f)							
12-Lead ECG	x		x	x(d)		x				x(f)							
MRI scan	x									x(f)							
Randomization				x													
PK/PD blood Sampling (e)	x(i)		x	x	x			x		x(f)	x	x	x	x	x		
Neuro Exam & EDSS	x									x(f)						x	x
Assessment of New Neurological Events	x	x	x	x	x	x	x	x	x	x(f)	x	x	x	x	x	x	x(g)
Concurrent Medical Procedures	x	x	x	x	x	x	x	x	x	x(f)	x	x	x	x	x		
Concomitant Medication	x	x	x	x	x	x	x	x	x	x(f)	x	x	x	x	x		
Adverse Events	x	x	x	x	x	x	x	x	x	x(f)	x	x	x	x	x	x(j)	x(j)
Drug administration				x	x	x	x	x	x	x (b)							
Confinement			x	x	x	x			x	x(f)	x						
Dispensation of Subject Trial/Alert card				x													
Subject Study Status															x		x

(a) V11 can be a phone call, in this case, vital signs, physical examination will not be done and EDSS will be done over the phone
(b) the last dose should be given as a morning dose
(c) only weight
(d) a telemetry will be performed from 12 h pre-dose till 36 h post-dose
(e) details are given in Table 11:2
(f) Measure/investigation to be performed either during V6 or if the subject terminates treatment prematurely
(g) Measure/investigation to be performed either during V12 or 12 weeks after early termination visit
(h) 30 min pre-dose, 30 min post-dose, 2.5 h post-dose
(i) only PD parameters
(j) only SAEs of following types: PML, serious infections (opportunistic infections, infections requiring parenteral or intra-muscular antibiotherapy and Tuberculosis cases), malignancies

6. BACKGROUND INFORMATION

6.1 Indication to be Studied and Medical Need

Multiple sclerosis (MS) is an inflammatory autoimmune disorder of the central nervous system (CNS). It is the most common cause of neurological disability in young adults leading to a major burden for patients, their families, and society at large. MS affects about two million people worldwide. Prevalence varies between ethnic groups and geography: from >1 per 1000 in Europe and North America to >1 per 100000 in Japan.

Symptomatology ranges from benign to devastating. Disability evolves over two to four decades. Though unremittingly progressive, the hallmark of MS are the unpredictable periods of exacerbation, remission, and progression. Dysfunction of body systems may lead to secondary damage. MS is rarely a 'fatal' disease but shortens the lifespan by 5 to 10 years.

Though the exact etiology remains unknown, MS likely results from interplay between environmental factors and susceptibility genes and also between inflammation and neuronal degeneration. Autoimmune inflammatory activity declines with time whereas degenerative neuronal processes increase with time. MS is characterized by inflammatory demyelination of the axonal myelin sheats and relative preservation of neuronal axons. Axonal loss may result from lacking trophic support rather than directly from inflammation but may also be related to intrinsic degenerative processes. Autoreactive T-cells cross the blood-brain barrier and enter the CNS under the influence of cellular adhesion molecules and pro-inflammatory cytokines. Infiltrates are rich in activated T cells and microglia with some neutrophils.

The currently used diagnostic McDonald criteria group patients by the categories of 'MS', 'not MS', or 'possible MS' [(1,2)] based on the concept of 'dissemination in time and/or space'; i.e., patients must have a minimum of two attacks affecting more than one anatomical site, but, assuming an initial clinical presentation suggestive of MS, the second 'attack' need not necessarily be a clinical one. A widely accepted clinical classification [(3)] distinguishes relapsing-remitting MS (RRMS), secondary progressive MS (SPMS), relapsing-progressive MS (RPMS), and primary progressive MS (PPMS). It has been proposed to sum up CIS (Clinically Isolated Syndrome), RRMS, and SPMS with superimposed relapses as 'relapsing forms of MS' or 'Relapsing MS' (RMS) [(4)] .

Mainstays of current treatment are beta-interferons (IFNBs) and glatiramer acetate (GA). Though used as first-line treatments, all are considered suboptimal. Main disadvantages are:

- a modest efficacy on the reduction of relapse rate of 25 - 35%,
- a not yet proven prevention on long-term accumulation of disability in RMS,
- a lack of efficacy on progression in patients with progressive forms of MS,
- the parenteral route of administration,
- poor tolerability, especially during initiation of treatment (except GA),

- development of neutralizing antibodies leading to a loss of efficacy (except GA).

There is a need for more potent drugs having one or more of the following desirable attributes while preserving the safety record of the available main treatments:

- better efficacy in reducing the relapse rate
- (better) efficacy to delay, halt, or reverse progression in all forms of MS,
- a more convenient, e.g., oral route of administration,
- better tolerability
- lack of neutralizing antibodies

The biological α4-integrin antagonist natalizumab seemed to offer a number of those attributes. However, the occurrence of progressive multifocal leukoencephalopathy (PML) under treatment with natalizumab has led to its temporary withdrawal from the market in early 2005. It has been re-approved for use in MS in 2006 for the US and has received also a marketing authorization in the EU during the same year. Recently, in late 2007, natalizumab was also approved for use in Crohn's Disease for the US.

PML is a serious, often fatal demyelinating disease almost exclusively observed in patients with impaired cell-mediated immunity. It is caused by the JC polyoma virus (JCV) and is considered an opportunistic infection. A large proportion of adults carry the latent virus. Three cases of PML have been observed under treatment with natalizumab. It is currently assumed that one per 1000 patients treated for a mean duration of 18 months may be at risk to develop PML (95% confidence intervals: 1:360 to 1:5000)[(5)].

The cause of PML in natalizumab-treated patients may be related to the actual mechanism of action (MoA), i.e., the prevention of normal lymphocyte trafficking by α4-integrin blockade leading to uninhibited JCV replication and subsequent spread of the infection to the CNS. Another hypothesis suggests blockade of α4 integrin is associated with increased numbers of B cells and immature progenitor cells released from the bone marrow: both of these cell populations may be reservoirs of latent JC virus. It can not be excluded that the actual occurrence of PML may require the combination of decreased immune surveillance and delivery of latent virus to the nervous system.

CDP323 and natalizumab share a common MoA but differ regarding the duration of biological effect after one dose: while natalizumab inhibits leukocyte trafficking for six weeks or even longer[(6)], CDP323 can exert the same effect for a maximum of three days. Thus, CDP323 may have the potential to offer a similar degree of efficacy while being more suitable for a comprehensive risk-management strategy. Furthermore, CDP323 will offer an oral route of administration and will - as a small molecule - not lead to the occurrence of neutralizing antibodies or injection site reactions. In summary, the development of CDP323 for MS addresses unmet medical needs for a serious medical condition.

6.2 The Investigational Product CDP323

Detailed information about CDP323 can be found in the Investigator's Brochure [7] .

CDP323 is a small-molecule pro-drug ester that is rapidly metabolized in vivo to its active forms, the carboxylic acid CT7758 (AM1) and CT533652-00 (AM2). Both metabolites have been shown to be a potent and selective antagonist of α4β1 and α4β7 integrins in cell-based assays.

6.2.1 Pharmacology

The integrins, a family of transmembrane glycoproteins, mediate several cell functions, e.g., adhesion, migration, activation, and cell survival. All leukocytes except neutrophils express the integrins α4β1 and α4β7. The ligand for α4β1 and α4β7 is the vascular cellular adhesion molecule-1 (VCAM-1), expressed on the vascular endothelium. The interaction between the α4 integrins and VCAM-1 is essential in facilitating the migration of leukocytes into surrounding tissues to mediate the effects of inflammation. Therefore, CDP323, via integrin inhibition, may reduce the ability of leukocytes to participate in the inflammatory response and may be effective at reducing tissue inflammation seen in diseases such as MS.

The primary pharmacodynamic of CDP323 and AM1 was assessed in a series of *in vitro* and *in vivo* assays. All models demonstrated that CDP323 and AM1 showed potency-specific binding to leukocyte subsets or a reduction-abolition of the inflammatory response to antigen challenge (cf. Investigator's Brochure [7]). Specifically, prophylactic dosing in mice significantly reduced disease severity of chronic experimental allergic encephalomyelitis (EAE), measured as a reduction in both cumulative and maximal clinical score. A significant reduction in disease incidence was also obtained. Therapeutic dosing in mice of CDP323 also produced a significant reduction in cumulative clinical score. CDP323 was further investigated in secondary pharmacodynamic studies assessing recruitment of monocytes and lymphocytes from cell trafficking assay in mice and rats. In both assays, recruitment was reduced by administration of CDP323 and AM1.

6.2.2 Metabolism and Pharmacokinetics

After oral administration of CDP323, the mouse and the dog were mainly exposed to AM1. There was no significant gender difference in the absorption, distribution, metabolism, and excretion of CDP323 and/or its metabolites. Excretion is primarily fecal in both species and almost complete within 24 hours.

A further metabolite (AM2) was identified later. Plasma concentrations of this metabolite, expressed as a percentage of AM1 concentration, were much higher in human subjects than in any of the animals studied (approximately 0.5% in the mouse, 2% in the dog and up to 40% in humans).

When investigated in healthy human volunteers, a wide inter- and intraindividual variation of pharmacokinetic parameters has been observed in all three studies conducted, though intraindividual variation occurred to a lesser degree. The following findings have been considered for CDP323 and its two main active metabolites when planning for this study:

For CDP323, t_{max} was approximately the same after all doses (range 1.0-2.5 h); $t_{½}$ appears to be below one hour. For AM1, measurable concentrations appeared at 15 - 30 min after dosing; t_{max} varied between 0.5 4 h after dosing and $t_{½}$ between 9 and 25 hours. For AM2, T_{max} varied little among dose groups (range 2.6-3.4 h). $T_{½}$ ranged from 5.9 to 7.3 hours. Administration after a high fat breakfast led to greater exposure to CDP323 and its two active metabolites than administration in the fasted state. Exposure generally increased with dose, but plasma concentrations of CDP323 and its two metabolites after 1,000 mg were higher than proportional to dose. Therefore, strict dose proportionality can not be inferred. No significant gender differences in PK parameters were noted.

Minimal levels of the administered dose were eliminated via urinary excretion in form of AM1 or AM2. The highest individual values of the fraction of the dose of CDP323 excreted, as urinary AM1 or AM2, during any 12 h collection period in any dose group, were 1.21% and 0.06% respectively.

From the estimates of IC50 on hepatic microsomal CYP450 enzyme activities, it is anticipated that AM1 and AM2 will not inhibit cytochrome P450 activities. AM1 induces CYP1A in vitro in human hepatocytes but has no effect on the expression of CYP2B6 or CYP3A4. Plasma protein binding of AM1 is high (> 97%) in all species.

6.2.3 Toxicology

CDP323 and its metabolites are of low acute toxicity with no adverse reactions in rat and mouse at limit or maximum practical doses.

When CDP323 was given up to 2000 mg/kg/day in the mouse and up to 750 mg/kg/day in the dog for 26 weeks, systemic target organ toxicity was restricted to liver changes noted in both the mouse and dog after 26 weeks. In the mouse, evidence of mild, reversible hepatotoxicity manifested as microvesicular vacuolation, suggestive of lipid accumulation, was observed. The NOAEL from this study was 150 mg/kg/day. In the dog, hepatotoxicity manifested as a combination of bile duct hyperplasia, oval cell hyperplasia and pericholangitis. Although bile duct hyperplasia and pericholangitis can be spontaneous findings in older Beagle dogs, the co-finding of oval cell hyperplasia is suggestive of a treatment effect. These findings were largely reversible. The NOAEL from this study was 200 mg/kg/day.

Additional toxicology studies were conducted in mouse and dog with AM2 to provide data on the safety of the higher plasma concentrations of this metabolite seen in human subjects.

Administered at doses limited only by local irritancy, the AM2 elicited neither systemic toxicity nor target organ pathology after subcutaneous (sc) administration. The NOAELs for systemic toxicity were set at the highest doses used in the four-week studies of 300 mg/kg/day for mouse and 30 mg/kg/day for dog.

CDP323 (and by inference AM1), and AM2 were assessed not to have genotoxic potential.

Administration of CDP323 during the period of gestation was associated with infrequent abortion and litter loss in rabbits and, in mouse, with embryofetal toxicity ranging from delayed fetal development through retarded fetal weight to morphological abnormalities that included a low incidence of cleft palate. NOAELs of 150 mg/kg/day for embryofetal toxicity were derived for both species.

During in vitro studies, CDP323 and AM1 were found to possibly have a phototoxic potential. AM2 was devoid of such findings.

In conclusion, the repeat-dose oral toxicology studies with CDP323 of up to 26 weeks duration and subcutaneous studies with the major human metabolite AM2 of up to 13 weeks duration provide toxicological cover to allow clinical dosing of up to 500 mg *bid* CDP323 for six months and up to 1000 mg *bid* for one month.

A complete summary of all toxicological studies performed with CDP323 and detailed safety margin calculations are presented in the Investigator's Brochure [7] .

6.2.4 Safety Pharmacology

Effects of CDP323, AM1 and AM2 on the cardiovascular, respiratory, gastrointestinal, and central nervous systems have been assessed in *in vitro* and *in vivo*.

In vitro cardiac electrophysiology studies have been performed using the hERG channel and the isolated Purkinje fiber model. AM1 at concentrations up to 60 μM, or AM2 at concentrations up to 30 μM, had no effects on either hERG channel or action potential parameters in canine cardiac Purkinje fibers. There was a 10% inhibition of the hERG channel with the pro-drug ester, CDP323 at a concentration of 25 nM but at higher concentrations up to 50 nM, CDP323 had no effects on resting membrane potential, maximum rate of depolarization, upstroke amplitude or action potential duration in canine Purkinje fibers. The effects of oral administration of CDP323 on cardiovascular function have been determined in conscious dogs and within repeat dose toxicity studies up to 26 weeks duration. There were no effects of a single oral dose of CDP323 up to 200 mg/kg on arterial blood pressure or heart rate in the conscious dog and a similar lack of effect of repeat dosing of CDP323 up to 750 mg/kg p.o. No QT effects and no consistent QTc effects were observed in the conscious dog at a plasma concentration of 687 ng/ml. The maximum concentration of CDP323 in the dog telemetry study and in the 26 week toxicology study

equates to approximately 10-fold and 20-fold higher, respectively, than the maximum plasma exposure obtained in man.

CDP323 (200 mg/kg p.o.) and AM2 (30 mg/kg s.c.) had no respiratory effects in conscious dogs. There were no unintended pharmacological effects on behavior and gastro-intestinal transit in mice after single oral doses of CDP323 up to 1500 mg/kg and post single subcutaneous doses of AM2 up to 300 mg/kg.

6.2.5 Pharmacodynamics

Pharmacodynamic evaluation at all doses studied demonstrated that CDP323 caused a decrease in the capacity of leukocytes to bind VCAM-1. Maximum inhibition of VCAM-1 binding was generally seen at either one or four hours after dosing. Degree and duration of VCAM-1 inhibition increased with higher dose, generally in a concentration-dependent manner. Minimum plasma concentrations after a 500 mg *bid* dose regimen are predicted to result in 80% receptor coverage for approximately 84% of human subjects. For comparison, a 1000 mg *bid* dose regimen is predicted to result in trough plasma concentrations achieving 80% receptor coverage for approximately 97% of subjects whereas a 250 mg *bid* dose regimen is predicted to achieve this degree of inhibition in approximately 50% of subjects. There was rapid recovery of VCAM-1 binding when dosing was stopped: mean inhibition was generally <20% at 96 hours after the last dose.

Pharmacodynamic simulations for the predicted inhibition of VCAM-1 binding following once daily dosing have not been performed, but the pharmacodynamic data from a single-dose study showed that the mean inhibition was 70% (range 47% to 88%) 24 hours after a single fasted administration of 500 mg CDP323 and 72% (range 56% to 84%) 24 hours after a single administration of 500 mg CDP323 with food, i.e. a 500 mg once daily dose regimen is likely to allow some recovery of α4/VCAM-1 binding capability during the dose interval for the majority of subjects.

Leukocyte numbers investigated during a multiple-dose study increased considerably after initiation of dosing and dropped rapidly after termination of dosing. This finding, together with rapid recovery of the α4/VCAM-1 binding capability, may indicate that a termination of treatment with CDP323 may lead to a quick recovery of a patient's immune function.

6.2.6 Clinical Profile

The clinical studies conducted so far included 75 healthy subjects, among which, 60 were dosed with CDP323. Safety was monitored with the following assessments: adverse events (AEs), blood pressure, heart rate, ECG, and laboratory safety testing. None of the safety assessments showed any changes that were considered to be clinically significant. The majority of AEs were mild in severity, none was significant or serious, and no subject was withdrawn because of an AE. The most common AEs during the studies were headache,

abdominal pain and nausea. Therefore, in the studies completed to date, CDP323 was considered to be safe and well tolerated at doses up to 1000 mg bid for 6 consecutive days.

The mean maximum plasma concentrations were reached between 1 and 4 h for CDP323, AM1, and AM2, with steady state levels of AM1/AM2 being reached on the second day of morning dosing. Administration of CDP323 after a high fat meal led to greater exposure to CDP323 and its metabolites than administration in the fasted state. Therefore, CDP323 will be administered with food. No significant gender effect was observed in any of the pharmacokinetic parameters; however there was considerable inter- and intra-subject variability in the PK parameters in all studies performed to date.

A Phase II study (C32322 [(8)]) in subjects with RMS is currently ongoing. It employs serial MRI measurements over a treatment period of 24 weeks. The doses investigated are 500 mg once daily and 500 mg *bid*. As of 31-Jan-2008, 37 subjects have been randomized of whom 3 have completed the treatment and are continuing in the drug-free follow-up period. The Independent Data Monitoring Committee (IDMC) for study C32322 has convened five times since the first site activation in April 2007. During the last meeting held on 31 January 2008, the IDMC concluded that the study can continue as designed. No safety concerns have been identified so far in patients participating in the C32322 study.

6.2.7 Known and Potential Risks and Benefits to Human Subjects

In the studies done to date, CDP323 has been found well tolerated in healthy male subjects at all doses studied as well as a single oral dose of 500 mg CDP323 tested in females. In-between, a number of subjects with RMS, including females, have been exposed to study drug in a Phase II study over periods up to 24 weeks.

During the Phase I studies, none of the safety assessments showed changes that were clinically significant, or could reasonably be attributed to the study drug. All AEs were mild in severity, none were significant or serious, and no subject was withdrawn because of an AE. The most common AEs during the studies, reported after placebo and active treatment, were headache, abdominal pain, and nausea. There was no relationship between the number of AEs and the dose of CDP323. The blinded safety-related information from the ongoing Phase II study has been described in Section 6.2.5; no new risk has been identified so far.

CDP323 and its metabolites are of low acute toxicity with no adverse reactions in rat and mouse at limit or maximum practical doses. Therefore, unintentional overdosing of CDP323 in the clinical setting would be unlikely to result in acute toxicity. However, no antidote for overdose would be available; care should be therefore supportive.

Natalizumab, a biological α4 integrin inhibitor sharing the same MoA, has been associated with the occurrence of PML. All three reported cases have been occurring in patients under concomitant treatment with other immunosuppressive or immunomodulatory treatment or for

whom a sufficient wash-out of such treatments may not have been achieved. One case occurred after five consecutive infusions of natalizumab (estimated treatment time 20 weeks), a second after 28 consecutive infusions (112 weeks), and the third after 37 infusions (148 weeks).

Of note should be that the patient with the shortest duration of treatment had received three earlier infusions of natalizumab (treatment-free interval: nine months). On theoretical grounds, the MoA may require additional vigilance to detect signals for a higher incidence of other opportunistic infections or malignancies. However, the evidence from the 3000 patients treated with natalizumab for a mean duration of 18 months and reviewed for a potential occurrence of PML does not provide any such signal. The duration of treatment in the current study is considerably shorter thus making the occurrence of opportunistic infections less likely. The nature of additional vigilance will have to weigh any risk related to such additional vigilance against its potential benefits.

Animal studies showed that CDP323 reaches the skin and the eyes and that the pro-drug and AM1 may have the potential to cause skin and eyes irritation after exposure to sun and ultraviolet light. Therefore the informed consent of this study contains recommendations for protection against light/sun and instructions for observation of potential skin changes. Guidance for the investigator can be found in the IB.

This study will implement the following measures to safeguard the health of the research participants:

- a comprehensive screening procedure to ensure that subjects with potential risk factors will not be randomized for treatment,
- a comprehensive surveillance for signs and symptoms indicative of PML,
- central reading of the post-treatment MRI scan with particular attention on potential signs of PML,
- training of all study personnel to exert the needed vigilance to recognize potential signs and symptoms of PML,
- clinical vigilance with regard to the occurrence of other opportunistic infection and/or malignancies including laboratory assessments such as lymphocyte phenotyping, tuberculosis and toxoplasmosis screen,
- withdrawal of study drug for research participants in need of treatment with immunomodulatory or immunosuppressive drugs,
- a Safety Advisory Board with relevant expertise and access to all safety-relevant data, including the MRI scans,
- information provided to subjects to ensure the necessary awareness to report to the study site any signs or symptoms potentially indicative of PML,
- follow-up contacts 3 and 12 months after completion of treatment,

In conclusion, the measures taken to safeguard the subjects' health should ensure that the risks currently attributable to the proposed administration of CDP323 do not exceed the typical risks for subjects during early phases of clinical drug development.

There is no other benefit than the one of a comprehensive and thorough medical check-up including MRIs though – theoretically – natalizumab's efficacy in reducing the relapse rate and progression to disability could be extrapolated to CDP323 due to the same MoA; however, this can not yet be confirmed at the current stage of development. Though beneficial MRI effects after treatment with natalizumab have been seen already after one month [(9)], it is not likely that a treatment period of four weeks will provide therapeutic benefit for the subjects. Thus – as the study duration will not allow subjects to benefit from treatment with CDP323 – subjects are considered 'symptomatic volunteers' and will be entitled to compensation appropriate for a Phase I study with a similar burden.

6.3 Justification of Study Design, Doses Selected, and Population

6.3.1 Rationale for Study Design

The design of a hybrid Phase I/II study was selected to allow frequent PK/PD measurements whilst taking necessary safety precautions in terms of exposure to a *bid* dose of 1000 mg CDP323 which has thus far only been investigated for a period of six days in healthy subjects. A double-blind, randomized, parallel-group design including the use of a placebo-group should enable a straightforward assessment of the observed effects.

The present study will be conducted in accordance with:

- this protocol;
- the International Conference on Harmonization:
  EU: CPMP/ICH/135/95 - ICH E6 Note for Guidance on Good Clinical Practice;
- the principles that have their origin in the Declaration of Helsinki;
- all applicable laws and regulations.

6.3.2 Rationale for Doses Selected

Study C32325 is designed to provide additional information on the exposure-response relationship for a wide dose range on a number of biomarkers (leukocyte-related parameters, α4 integrin expression on VCAM-1 binding cells in subjects with RMS treated with CDP323 or placebo over a period of four weeks. The CDP323 doses to be investigated comprise:

- 100 mg *bid* given over four weeks;
- 500 mg *bid* given over four weeks;
- 1000 mg once daily given over four weeks;
- 1000 mg *bid* given over four weeks.

Study C32325 will contribute to finding the minimally effective dose of CDP323 in RMS therapy and will also provide further information on the safety, tolerability, and biomarker effects of a dose higher than selected for the ongoing Phase II study, i.e., 1000 mg *bid*. 1000 mg *bid* has been found to be well tolerated in healthy volunteers and appeared to affect peripheral leukocyte levels in healthy subjects to a similar degree as natalizumab did in patients with MS employing the doses of 3 and 6 mg/kg.

In addition, this study intends to investigate 1000 mg given once daily to complement the scope of administration schedules investigated in Phase I and Phase II. The 1000 mg once-daily application was chosen to evaluate how a partial recovery of VCAM-1 binding during the dose interval in the majority of subjects would affect the trafficking of leukocytes. Theoretically, such partial recovery may offer advantages with regard to the safety of the class of α4-integrin inhibitors as the observed occurrence of PML in patients treated with natalizumab may have been related to the continuous prevention of normal lymphocyte trafficking by α4-integrin blockade. Furthermore, a once-daily administration scheme – once established as effective – may lead to a better patient adherence in the long-run. This is of particular importance for indications like MS requiring chronic treatment.

The current study will investigate CDP323's PD at doses greater than that permitted in study C32322 as toxicological cover for human dosing at doses greater than 500 mg *bid* is currently restricted to a duration of no more than four weeks (the duration of dosing in the Phase II study C32322 is 24 weeks). Administration of a higher dose (1000 mg *bid*) in a controlled Phase I setting will also judiciously contribute to the knowledge about safety and tolerability at the upper end of CDP323's dosing range. Non-clinical coverage for treatment of four weeks is available as follows:

- Repeat dose oral toxicology studies in dogs with CDP323 of up to 26 weeks duration and subcutaneous studies with the major human metabolite AM2 of up to 13 weeks duration provide toxicological cover to allow clinical dosing of up to 500 mg CDP323 *bid* for six months and up to 1000 mg *bid* for four weeks.
- Systemic target organ toxicity elicited by CDP323 was restricted to liver changes noted in both mouse and dog after 26 weeks treatment. In the mouse, evidence of mild, reversible hepatotoxicity manifested as microvesicular vacuolation, suggestive of lipid accumulation, was observed. The NOAEL from this study was 150 mg/kg/day.
- In the dog, hepatotoxicity manifested as a combination of bile duct hyperplasia, oval cell hyperplasia and pericholangitis. Although bile duct hyperplasia and pericholangitis can be spontaneous findings in older Beagle dogs, the co-finding of oval cell hyperplasia is not. These findings were largely reversible. The NOAEL from this study was 200 mg/kg/day.
- CDP323 and its metabolites are of low acute toxicity with no adverse reactions in rat and mouse at limit or maximum practical doses and were assessed not to have genotoxic potential.

6.3.3 Rationale for the Population Selected

The population of subjects with RMS – as opposed to healthy volunteers – was selected to evaluate the effects on biomarkers in the target population with an existing inflammatory stimulus and to avoid a repeated exposure of healthy volunteers over 4 weeks with an α4 integrin inhibitor.

In conclusion, study C32325 will allow a better-informed dose selection for the pivotal clinical development program in combination with the read-out of the Phase II study C32322.

7. STUDY OBJECTIVES

7.1 Primary Objective

The primary objective is to compare the effects of different CDP323 doses (100, 500, and 1000 mg *bid* as well as 1000 mg once daily) given over a period of four weeks on biomarkers (leukocyte-related parameters, degree of VCAM-1 inhibition, proportion of α4-receptor expression) in subjects with RMS.

7.2 Secondary Objective

To assess the safety and tolerability of 1000 mg CDP323 in once daily and twice daily administration over the period of four weeks in subjects with RMS.

7.3 Exploratory Objective

To assess the exposure-response relationship between CDP323 (and its metabolites) and changes in biomarkers relative to changes observed with placebo.

8. STUDY DESIGN

8.1 Type/Design

This is a hybrid Phase I/II study and will be conducted as a double-blind, randomized, placebo-controlled, dose-controlled study.

Male and female subjects with RMS between the ages of 18 and 65 years will be recruited to participate in this study. Subjects must have a score of 6.5 or less on the Expanded Disability Status Scale (EDSS) and must be free of other conditions potentially compromising the immune response. Furthermore, subjects must not take any concomitant immunosuppressive or -modulatory medication including registered or putative disease-modifying treatment against MS during exposure to CDP323 and within a thirty-day period after completing study treatment or after withdrawing from study treatment. Females of childbearing potential will

be permitted to participate if they are using appropriate contraception (see inclusion criteria for details).

The subjects will be divided in five groups of 14 subjects each. All subjects will take study medication twice a day for a period of 28 consecutive days (Day 1 till Day 28). The last intake will be the morning dose of Day 28. This may comprise the intake of placebo as assigned.

Table 8:1 Dosing groups

	**Treatment Period : 4 weeks Visits 3 - 6 Days 1 to 28**	
	**morning**	**evening**
**Group 1**	1000 mg	placebo
**Group 2**	100 mg	100 mg
**Group 3**	500 mg	500 mg
**Group 4**	1000 mg	1000 mg
**Group 5**	placebo	placebo

In order to prudently manage any unexpected untoward events, all subjects will be hospitalized for the first five days (5 nights) during the treatment period. Further hospitalization will also occur from day 27 evening until day 29 morning, during the more intensive PK/PD blood samplings and can be prolonged until day 35 if deemed necessary by the investigator.

PK sampling will occur over 0-24 hr after the first morning dose, at pre-dose on day 15 and over 0-168 hr after the last dose. Sampling for PD assessments will occur during most PK sampling timepoints. In order to maintain the blind, PD data (including all leukocyte-related parameters from hematological tests) will not be made available to the study center personnel until after study unblinding, with the exception of leukocyte levels above 15000 x 10^9/l or below 500 x 10^9/l and CD4+ counts below 200 x 10^9/l.

Subjects who drop out prior to randomization will be replaced; however, there will be no replacement of subjects dropping out later, including subjects suffering from a relapse.

Subjects will be asked to report back to the study center for a safety check 3 and 12 months after administration of the last dose. The 3-month follow-up visit may be carried out by telephone while subjects are expected to be on site for the 12-month follow-up visit. Subject in need of DMT for MS should be advised to refrain from such or other treatment affecting the immune system for 30 days after terminating treatment with CDP323 (pulsatile steroid treatment for relapses is exempted from this rule). Investigators will be advised to discuss the possibility of approved MS treatments with respect to the individual situation.

Should the results of the mentioned Phase II study C32322 [8] indicate that CDP323 could be an effective and safe MS treatment and should UCB decide to continue developing it for the treatment of subjects with RMS, all subjects that have completed the full study will be offered to participate in an open long-term safety study once such a study has been approved by the competent review bodies. Completion of the study for the purpose of accessing the open safety study is considered once the subject has completed her/his 3-month follow-up visit. It is currently intended to offer such open-label access to until it will be available through normal NHS prescription or until an eventual stop of drug development.

8.2 General Design Considerations

Due to the occurrence of PML, an opportunistic infection caused by the JC polyoma virus, in three patients who received α4 integrin inhibitor natalizumab, attention will be paid to the detection and monitoring of blood JC viral DNA before and throughout the duration of the study. At the time of study inclusion, subjects should be free of signs of viral activation (i.e., no detectable blood JC viral DNA) measured twice at a one-week interval. Further, the use of MRI will contribute to enhance the safety of the subjects.

In addition to PML-related safety precautions, clinical safety examinations, routine blood chemistry and hematology analyses, as well as immunophenotyping, and urinalyses will be performed at regular intervals during the treatment periods. As mentioned previously, the leukocyte parameters will not be made available to the site personnel during the study in order to preserve the blind. However, this data will be reviewed on an ongoing basis throughout the study by an independent and experienced physician affiliated to the Safety Advisory Board.

Subjects will be fully informed about the risks associated with participation in this study. They will be made aware of early indicators for the potential development of PML and will be asked to report the occurrence of such indicators immediately to the study site. Subjects will further be asked to inform persons close to them (e.g., persons living in same household) about their study participation and the potential implications and to consent to such information being given to their family doctor and/or neurologist. In addition, subjects will be asked to consent to potentially necessary measures to assess a differential diagnosis of PML, including an additional MRI scan and sampling of cerebrospinal fluid.

Though CDP323 has not been implicated with effects on cardiac repolarization, it has not yet been fully characterized with regard to cardiac repolarization. Therefore, treatment with drugs known for their potential to interfere substantially with cardiac repolarization will be excluded until CDP323's effects on cardiac repolarization have been fully characterized.

The study will be followed by a Safety Advisory Board (SAB). However – as the study is rather short – there will be no formal safety interim analyses. Therefore, the board will not

function as a classic Independent Data Monitoring Committee. The SAB will have access to information describing a subject's treatment in case of need and will monitor the safety-related study information on an ongoing basis. The monitoring will encompass all significant on-study events, including SAEs, and potential alerts about:

- clinical signs and symptoms atypical for MS or suggestive of PML,
- any detected JC virus load, or
- MRI findings in support of a diagnosis of PML.

The functioning of the SAB and its monitoring activities will be described in a SAB Charter. The safety-related procedures will be described in an algorithm integrating the safety-relevant areas (clinical investigations, imaging, and laboratory findings).

8.3 Subjects/Sites Numbers

Overall, 70 subjects with RMS will participate in study C32325. Subjects will be randomized to five groups, each comprising 14 subjects. The study will be performed under the supervision of one CRO in 4-5 units having the capability to hospitalize subjects, and being staffed and equipped to handle emergency situations.

8.4 Measures to Minimize/Avoid Bias

8.4.1 Blinding

All UCB, investigational site, and CRO staff involved with the study will be blinded to the treatment code with the following exceptions:

- UCB personnel directly involved in manufacturing/packaging of the study medication (no direct contact to investigational sites),
- UCB pharmacovigilance personnel reporting SAEs to regulatory authorities (unblinding for single cases only),
- SAB members (unblinding for single cases only).

The following UCB staff members or CRO personnel may be unblinded by generating or handling data:

- UCB personnel analyzing blood samples for drug plasma concentrations (no direct contact to investigational sites),
- Laboratory personnel analyzing blood samples for leukocyte parameters (no direct contact to investigational sites).

Under normal circumstances, the blinded treatment must not be revealed. In the case of a medical emergency, UCB or its representatives should be contacted. The blind may be broken if doing so will aid in the decision as to the subject's treatment or clinical intervention. UCB or its representatives must be notified immediately if the blind is broken. Any unblinded subjects will be withdrawn from the study.

Sealed envelopes will be provided to the Investigator. The envelopes must be stored safely by the Investigator or other responsible study site personnel. During the study, the integrity of the sealed envelopes will be examined by the Monitor during the routine monitoring visits. Investigators are advised to contact UCB or its representatives prior to unblinding the treatment of subjects if possible. If unblinding is medically necessary, the investigator or another authorized person may only unblind the treatment code for one individual subject.

One set of unblinding envelopes will be made available to the chairperson of the SAB. The SAB may unblind individual subjects to perform its surveillance tasks. It may only unblind the treatment code for a concerned subject while leaving the blind for the remaining subjects intact.

For the purpose of information about study participation and emergency unblinding, UCB will provide each Investigator with an appropriate quantity of Alert & Subject Cards for subjects. Each subject will be instructed to keep it with him/her at all times. The Investigator will fill in each card with the details of his/her contact information (e.g., Investigator stamp) and subject identifier. The card will be distributed to the subject with the first box of study drug.

8.4.2 Randomization

The randomization list will provide the randomization numbers used in the subject identifier. The randomization list will be produced by UCB Clinical Supply Unit (CSU) using a validated program (using random permuted blocks process) provided by the UCB Biostatistics Department.

This randomization list will be retained by the CSU until the end of the study (i.e. until all data are entered into a database, the pre-analysis review of data took place, the statistical analysis plan is approved and the database has been locked). Seventy subjects will be randomized to 5 different treatment groups.

One copy of the randomization list will be sent before the start of the trial in a sealed envelope directly from CSU to the Laboratory of Drug Metabolism and Pharmacokinetics of UCB, who will be unblinded to the treatment code in order to only perform analysis on biological samples coming from subjects under investigational product.

8.4.3 Subject Identifier

Each subject will be identified by initials and a unique identifier including study number, center number and a sequential number (from 0001 to nnnn).

This sequential number corresponds to the randomization number. Since this randomization number is only allocated at the randomization visit (visit 3, Day 1), the subject's identification prior to this visit is restricted to the subject's initials, center number and CRF number. As soon as the subject has received its own randomization number, the Investigator is requested to complete retrospectively the full subject's identification number in the header of the CRF, starting at the cover page.

For the screen failure or not randomized subjects, the sequential randomization number will be replaced by a sequential number from 9001 to 9nnn and a screen failure booklet will be filled out.

8.5 Study Duration

The experimental portion of the study will last approximately 8 weeks and will include:
- A screening period up to three weeks before the first dose of study medication;
- A treatment period lasting four weeks with PK/PD assessments until one week after last dosing.

Subjects will be asked to report back to the study center for a safety check 3 and 12 months after administration of the last dose.

8.6 End of Study

The end of study is defined as the date of Last Patient Last Visit in the experimental phase (V10) after which the pre-analysis will be done, the database will be locked and the unblinding will be performed.

9. SELECTION AND WITHDRAWAL OF SUBJECTS

Before any study procedures are initiated for any subject in this study, an Independent Ethics Committee (IEC) approved written informed consent form will be properly executed and documented.

9.1 Subject Inclusion Criteria

To be eligible to participate in this study, all of the following criteria must be met:
Written informed consent signed and dated by the subject;
- Female and male subjects aged 18-65 years inclusive at time of informed consent;
- Being of legal capacity and able to understand the nature of the study and its potential risks;
- Diagnosis of MS according to the revised McDonald criteria [1,2] ;
- Relapsing form of MS, i.e., RRMS or SPMS (with superimposed relapses) according to Lublin and Reingold [3] with at least one clinical relapse in the 24 months before screening and documented in the subject's medical records;

- Screening EDSS score of 0-6.5, inclusive;
- Screening MRI must conform with the diagnosis of MS;
- Must be fully immunocompetent with a CD4+ count >500;
- No detectable JC viral DNA in blood (serum and PBMCs) at two pre-scheduled consecutive measurements a week apart from each other during the screening period;
- Willing to comply with the study safety precautions and with the follow-up at 3 and 12 months after the last study drug intake;
- Female subjects of childbearing potential must agree to practice one of the following contraception method:
  - double-barrier contraception (i.e., using a male or female condom or a diaphragm or cervical cap, all in conjunction with spermicide) or
  - any systemic hormonal contraceptives (i.e., 'the pill', all mini pills, implants, patches, depot injections) plus one of the following: diaphragm or cervical cap, male or female condom, all in conjunction with spermicide, or
  - intrauterine devices releasing levonorgestrel (Mirena), or
  - intrauterine devices without levonorgestrel plus one of the following: male or female condom, diaphragm or cervical cap, all in conjunction with spermicide, or
  - monogamous relationship with partner vasectomized since at least 3 months

  Note: Lack of childbearing potential will be considered under these circumstances:
    - post-menopausal for at least two years;
    - hysterectomy or bilateral oophorectomy, ovariectomy, salpingectomy, or tubal ligation;
    - true sexual abstinence;
    - congenital sterility.
- Sexually active males with partners of child bearing potential must use two approved methods of contraception. In addition, sexually active males must use a condom if their female partner is pregnant or breast feeding. Contraception precautions should continue for at least 3 months after the last dose of CPD323.

9.2 Subject Exclusion Criteria

Subjects must be excluded if they meet any of the following criteria:

- Any disease other than MS that could better explain the subject's signs and symptoms;
- Any conditions that could interfere with the contrast-enhanced MRI, including an estimated glomerular filtration rate (eGFR) <40 ml/min, or with any other evaluation in the study;
- Any clinically significant disease state or findings other than MS, in particular neoplastic disease or organ transplantation;
- Any clinically significant deviation from the pre-defined ranges for laboratory tests or;
- Signs of silent infections, including positive tests for HIV1, HIV2, or Hepatitis B or Hepatitis C, toxoplasmosis IgM, or tuberculosis;
- Known hypersensitivity to gadolinium-containing contrast agents,

- History of clinically significant severe AEs to any drug;
- Participation in any clinical drug trial within 30 days prior to screening;
- Pregnancy or lactation;
- History of alcohol or drug abuse within the year before screening;
- Medical, psychiatric or other conditions that compromise the subject's ability to understand the subject information, to give informed consent, to comply with the trial protocol, or to complete the study;
- Any abnormal, clinically significant ECG findings, in particular any marked pre-study prolongation of the QTcF interval >470 ms
- Known hypersensitivity to ingredients of the CDP323 drug formulation
- Any condition possibly interfering with drug absorption;
- Concomitant treatment with:
    - the CYP1A substrates mexiletine, propafenone, theophylline, verapamil or warfarin, or
    - statins (e.g., atorvastatin), protease inhibitors (e.g., ritonavir), systemic triazole antifungals (e.g., ketoconazole) for their interference with transport protein systems or for their strong inhibition of the P450 cytochrome pathway, or
    - drugs known for their potential to substantially interfere with cardiac repolarization: amiodarone, arsenic trioxide, bepredil, budipine, cisapride, chinidine (quinidine), chloroquine, chlorpromazine, clarithromycin, disopyramide, dofetilide, domperidone, droperidol, erythromycin, halofantrine, haloperidol, ibutilide, levomethadyl, mesoridazine, methadone, pentamidine, pimozide, procainamide, sotalol, sparfloxacin, thioridazine;
- Treatment with the following substances prior to screening within the following time frames:
    - at any time: cyclophosphamide; total lymphoid irradiation; anti-lymphocyte monoclonal antibody treatment (e.g., anti-CD4, Campath-1H);
    - 12 months prior to screening: natalizumab, rituximab, or mitoxantrone.
      Note: prior treatment with natalizumab or rituximab must not have been terminated for safety reasons.
    - 6 month prior to screening: azathioprine, cyclosporine A, human antibodies, or other strong immunosuppressive drugs;
    - up to 30 days prior to screening: any interferons, glatiramer acetate, corticosteroids, ACTH, IvIg, any other immunomodulating or immunosuppressive drugs including recombinant cytokines, any other putative or experimental MS treatment; any inoculation with attenuated live vaccines;
- Any other reason why, in the investigator's opinion, the subject should not participate.

If a subject suffers a relapse during the screening phase, s/he may not be randomized on the planned visit (V3, Day 1). S/he may be randomized 30 days ± 3 days after the end of the last relapse and must undergo a new full screening procedure.

9.3 Subject Withdrawal

Subjects are free to withdraw from the study at any time, without prejudice to their continued care. If a subject wishes to withdraw from the study, but has already taken at least one dose of study drug, he/she will be advised to attend an early termination visit as soon as possible and then the two drug free follow up visits at 3 and 12 months after the early termination visit.

Subjects should be withdrawn from treatment if they meet any of the following criteria:

- request of subject to terminate treatment and/or to withdraw from the study;
- at the discretion of the investigator at any time for any reason;
- at the discretion of the Safety Advisory Board at any time for any reason;
- unauthorized use of study drug;
- broken blind;
- Persistence of any severe AE including deviation from reference ranges in laboratory tests (all AEs Grade 3 or 4 according to Common Terminology Criteria for Adverse Events (CTCAE) v3.0 are considered 'severe' all AEs Grade 4 according to the CTCAE are considered 'serious') see section 17.3.
  Note: on occurrence of any leukocyte toxicity or aminotransferase toxicity grade 3 or 4 according to the Common Toxicity Criteria, also without clinical signs or symptoms, study medication should be discontinued until grade 2 toxicity or lower is reached. If grade 3 or 4 leukocyte or aminotransferase toxicity persists longer than 10 days, treatment with study drug should not be resumed.
- a Fridericia-corrected QT interval (QTcF) consistently higher than 500 ms or a QTcF increase consistently beyond 60 ms over the value calculated for QTcF at Baseline (V3, day -1)
- presence of JC viral DNA in blood evidenced by positive testing in serum or PBMCs during two separate measurements;
- presence of JC viral DNA in the cerebrospinal fluid (CSF);
- clinical worsening as evidenced by either neurological worsening atypical for MS or cognitive/behavioral worsening, either lasting longer than 14 days, also in the absence of MRI or laboratory findings of a beginning PML;
- any signs or symptoms of PML either seen during imaging or clinically;
- any signs or symptoms of an infectious disease with a potential for a life-threatening or disabling course or having a clinically significant unusual or prolonged course;
- Positive urine pregnancy test in women of childbearing potential;
  Note: Subjects from whom study drug treatment has been withdrawn due to pregnancy may not undergo MRI scanning while pregnant.
- medical need for treatment and/or concomitant treatment with a licensed or putative immunomodulating or immunosuppressive drug, irrespective of indication for drug treatment including systemic steroid therapy, if not given as a treatment for an acute relapse or medical need for treatment with an excluded concomitant medication;

- participation in any other clinical trial.

Investigators should attempt to obtain information on subjects who withdraw or discontinue. The Investigator should make every effort, and document his/her effort, to complete the visit 6 (end of treatment) and follow-up evaluations (phone calls, fax, letters). All results of these evaluations and observations, together with a narrative description of the reason(s) for removing the subject, must be recorded in the source documents. The Case Report Form (CRF) must document the primary reason for withdrawal or discontinuation.

All subjects having received study drug should be asked to participate in the follow-up after 3 and 12 months.

The study medication can be discontinued without down-titration. Non-randomized subjects can leave the trial immediately. After a decision of a subject to withdraw from the study, the Investigator will provide the subject with information about alternative treatments. Subject in need of DMT for MS should be advised to refrain from such or other treatment affecting the immune system for 30 days after terminating treatment with CDP323 (pulsatile steroid treatment of relapses is exempted from this rule).

Subjects withdrawn after randomization will not be replaced.

10. TREATMENT OF SUBJECTS (INVESTIGATIONAL PRODUCT AND CONCOMITANT MEDICATIONS)

10.1 Study Investigational Products

10.1.1 Description of all Investigational Products

The investigational product (including placebo) will be supplied under the responsibility of the UCB Global Clinical Supply Unit to one central depot. The frequency at which investigational product will be supplied to each individual center will be adapted to the availability of investigational product, recruitment capacity of that center, and to the expiry date of the investigational product and will be managed by Richmond.

CDP323 will be administered orally and be given as hard-gelatin capsules containing 50 and 250 mg CDP323. Two placebos will be provided as matching hard-gelatin capsules for each dosage.

10.1.2 Packaging

The Investigational Medicinal Products are manufactured, packaged, and labeled according to current Good Manufacturing Practices (cGMP), guidelines and applicable national laws and

regulations. They are suitably packaged, in such a way as to protect the product from deterioration during transport and storage.

Trial medication will be provided in blisters, each containing sufficient tablets for 1-week twice daily dosing, with 1 day of reserve medication. The strengths of CDP 323 to be used are 50 or 250 mg. Two placebos will be provided as matching hard-gelatin capsules for each dosage. Table 10:1 displays the dosing schedule.

Table 10:1 Dosing schedule

	**morning**	**evening**
**Group 1**	4 cps 250 mg<br>+<br>2 cps placebo size 3<br>total dose: 1000 mg	4 placebo cps size 0<br>+<br>2 cps placebo size 3<br>total dose: 0 mg
**Group 2**	4 cps placebo size 00<br>+<br>2 cps 50 mg<br>total dose: 100 mg	4 cps placebo size 00<br>+<br>2 cps 50 mg<br>total dose: 100 mg
**Group 3**	2 cps placebo size 00<br>+<br>2 cps placebo size 3<br>+<br>2 cps 250 mg<br>total dose: 500 mg	2 cps placebo size 00<br>+<br>2 cps placebo size 3<br>+<br>2 cps 250 mg<br>total dose: 500 mg
**Group 4**	4 cps 250 mg<br>+<br>2 cps placebo size 3<br>total dose: 1000 mg	4 cps 250 mg<br>+<br>2 cps placebo size 3<br>total dose: 1000 mg
**Group 5**	4 cps. placebo size 00<br>+<br>2 cps placebo size 3<br>total dose: 0 mg	4 cps placebo. size 00<br>+<br>2 cps placebo size 3<br>total dose: 0 mg

10.1.3 Labeling

The Investigational Medicinal Products will be labeled in accordance with the current ICH guidelines on GCP and GMP and will include any locally required statements. The label will be adapted to the size of the investigational product package.
The labels for trial medication will include the following information (not all information will appear on all labels:

- Sponsor name and address

- Name of drug
- Pharmaceutical dosage form
- Strength/Potency of drug
- Route of administration
- Trial number
- Kit number
- Directions for use
- Quantity of dosage units
- Subject number (to be completed upon dispensing)
- Name and phone number of Investigator (to be completed upon dispensing)
- Batch number
- Expiry date
- Statement 'For clinical trial use only';
- Statement 'Keep out of reach of children';
- Statement: 'Return all empty, partially used and unused kits"
- Storage conditions

Flat label with tear-off part will be used for treatment kits in order to put the tear-off part in the CRF.

10.1.4 Storage Requirements

Investigational medicinal product packages must be stored in a secured, limited access area, between 15°C and 25°C. The Investigator (and hospital pharmacist, if applicable) is responsible for the appropriate storage of investigational medicinal product at the site and should convey storage conditions to each study subject.

10.1.5 Monitoring of Subject Compliance

At each visit after drug is dispensed, subjects must return to the site with all the blisters they received to allow for the check of their compliance. Each blister contains the medication for 7 days and one day reserve medication. Drug accountability must be done in the subject's presence in order to obtain explanations regarding discrepancies in compliance with the dosing regimen.

The number of dosage units returned as well as explanations of non-compliance must be recorded on the CRF. Compliance with study medication is defined as investigational product consumption by the subject within 80 - 120% of the prescribed dosage.

10.1.6 Investigational Products Accountability

Each Investigator will receive numbered treatments.

The Sponsor, or its representatives, will supply a Drug Accountability Form, to be kept up-to-date by recording all study drug received during the course of the study and study drug released for subject use. Details of any drug lost (due to breakage or wastage), not used, disposed of at the study site or returned must also be recorded. All supplies and pharmacy documentation must be kept at site and made available throughout the study for the Sponsor or designee to review.

Drug Accountability Forms should include at least:

- Number of capsules dispensed to and returned by each subject, with the visit number and subject's number;
- Treatment kit number and kit week number(s) ;
- Initials of the person who actually dispensed and/or received returned trial medication;
- Dates of the above;
- Explanation of non-compliance.

Periodically and/or after completion of the trial, all used (including empty containers) and unused investigational product containers must be reconciled and returned to the Sponsor or designee, preferably in their original package. Clinical drug supplies intended for the study cannot be used for any other purpose than that described in this protocol.

10.2 Concomitant Treatments and Rescue Medications

Should any treatment other than the investigational product be used, including over-the-counter products, an accurate record must be kept in the clinic chart (source documentation) and the Case Report Form. This record should include the name of the drug, the dose, the date(s) of administration, and the indication for use.

10.2.1 Rescue Medication

Not applicable.

10.2.2 Permitted Concomitant Treatments (Medications and Therapies)

Pre-clinical data showed that AM1 (one of the CDP323 metabolites) slightly induced CYP1A activity. Therefore, care should be exercised when using CDP323 together with CYP1A substrates: subjects should be observed for diminished activity of CYP1A substrates and dosages of the following substrate drugs may have to be adapted according to clinical need:

> amitriptyline, caffeine, clomipramine, clozapine, cyclobenzaprine, estradiol, fluvoxamine, haloperidol, imipramine, naproxen, olanzapine, ondansetron, phenacetin = acetominophen, propranolol, riluzole, ropivacaine, tacrine, tizanidine, zileuton,

zolmitriptan (cave: the use of the CYP1A substrates mexiletine, propafenone, theophylline, verapamil, and warfarin is prohibited, see Section 10.2.3)

As estradiol is also a CYP1A substrate, any contraceptives containing estrogens have to be used in combination with double-barrier contraception while being exposed to CDP323.

In case of a relapse requiring steroid treatment, intravenous methylprednisolone 500 - 1000 mg/d may be given over 3 - 5 days (if indicated, ranitidine 150 mg may additionally be given at night during the treatment cycle). Treatment with study medication has to be interrupted during steroid treatment.

10.2.3 Prohibited Concomitant Treatments (Medications and Therapies)

Use of the CYP1A substrates mexiletine, propafenone, theophylline, verapamil, and warfarin is prohibited.

Though CDP323 has so far not been implicated with effects on cardiac repolarization, the compound has not yet been fully characterized with regard to its potential effects on cardiac repolarization. Until those effects have been fully characterized, treatment with the following drugs known for their potential to interfere with cardiac repolarization will be excluded: amiodarone, arsenic trioxide, bepredil, budipine, cisapride, chinidine (quinidine), chloroquine, chlorpromazine, clarithromycin, disopyramide, dofetilide, domperidone, droperidol, erythromycin, halofantrine, haloperidol, ibutilide, levomethadyl, mesoridazine, methadone, pentamidine, pimozide, procainamide, sotalol, sparfloxacin, thioridazine.

Statins (e.g., atorvastatin), protease inhibitors (e.g., ritonavir), systemic triazole antifungals (e.g., ketoconazole) are prohibited for their interference with transport protein systems or for their strong inhibition of the P450 cytochrome pathway.

Concomitant treatment with any marketed or putative MS drug is prohibited, in particular with natalizumab, mitoxantrone, cyclophosphamide, rituximab, anti-lymphocyte monoclonal antibody treatment (e.g., anti-CD4, Campath-1H), any interferons, glatiramer acetate, azathioprine, IvIg, cyclosporine A, human antibodies, chronic systemic treatment with steroids, any other immunomodulating or immunosuppressive drugs including recombinant cytokines, or any other putative or experimental MS treatment. Subjects in need of treatment with an immunomodulating or immunosuppressive drug, irrespective of indication for drug treatment, have to be withdrawn.

11. STUDY PROCEDURES

11.1.1 Informed Consent

Before any trial-related procedures are performed, an IEC approved informed consent will be properly executed and documented.

11.1.2 Demographic Data

At the first visit (V1), date of birth, race and ethnic group will be recorded.

11.1.3 General Medical and Procedures History

At the first visit (V1), a complete general medical history and a history of medical interventions, will be obtained. This will also include queries regarding the subject's current or past use of tobacco products, consumption of alcohol, and use of illicit drugs.

11.1.4 MS History and Diagnosis

At the first visit (V1), the history of MS will be documented, including:

- Date of onset (onset of first clinical symptom)
- Date of diagnosis
- Total number of relapses
- Number of relapses during the last two years
- Number of relapses during the last year
- Date of onset of last relapse
- Number of sub-clinical exacerbations during the last two years
- Number of sub-clinical exacerbations during the last year
- Number of lesions (suggested by objective clinical evidence)
- Diagnosis of MS according to the revised McDonald criteria [(1,2)] . The MS diagnosis is the responsibility of the investigator. The CRU-MRI will not contribute to establishing the MS diagnosis at screening.

11.1.5 MS Medication History

The DMT history should be captured since onset of MS during the first visit (V1). DMT to be captured include interferons, glatiramer acetate, azathioprine, IvIg, cyclosporine A, human antibodies, natalizumab, mitoxantrone, or cyclophosphamide, total lymphoid irradiation, anti-lymphocyte monoclonal antibody treatment, or any other immunomodulating or immunosuppressive drugs intended to modify the course of the disease including any experimental MS treatment.

11.1.6 Vital Signs

Vital signs are measured during all personal visits to the study site except V2. The measurement comprises temperature (axillary, orally or intrauricularly), respiratory rate, heart rate (in supine position after 5 minutes rest), and systolic and diastolic blood pressure (in supine position after 5 minutes rest). If clinically significant deviations are found, the measurements are to be repeated before documentation. Subjects who are eligible for the study and subsequently randomized will have additional vital signs monitoring at the following time-points: 30 minutes before, 30 minutes and 2.5 hours post initial drug administration.

11.1.7 Physical Examination

During the Screening Period (V1), V3 (day 1 pre-dose), V5, V6 (day 28), V11 and V12, a standard physical examination will be performed which will include investigation of skin, eyes, ear, nose, throat, cardiovascular system, respiratory system, gastro-intestinal system, musculoskeletal system, endocrine, metabolic and nutritional system, blood and blood-forming organs, and immune system and genitourinary system (optional).

Weight will be measured at Screening (V1) and at V6. Height will be measured at Screening (V1).

Particular attention should be paid to any signs of an infection and/or the eventual development of malignancies during all assessments.

11.1.8 Childbearing Potential and Birth Control

From screening (except V2) till the end of treatment, information on childbearing potential of female subjects and contraceptive method used by female subjects will be collected and captured in the source documents. Lack of childbearing potential will be considered under the following circumstances:

- Post-menopausal for at least two years;
- Bilateral oophorectomy, ovariectomy, salpingectomy, or tubal ligation;
- Hysterectomy;
- True sexual abstinence (in this case, the pregnancy tests will still be performed).
- Congenital sterility;

During the course of the trial, the Investigator should make sure that birth control remains in line with the provision of the inclusion criteria (see Section 9.1), also in case of a change of the status of a female subject.

For female subjects of childbearing potential or claiming sexual abstinence, a pregnancy test will be performed at Screening (V1; ßHCG blood test), at V3 (day -1, urine test) and pre-dose

at V6 (may be done on the evening of day 27, urine test). All female subjects of childbearing potential will be informed to immediately discontinue study drug treatment upon pregnancy.

11.1.9 Laboratory

Laboratory assessments will be conducted using standard methods at a Central Laboratory. The Central Laboratory will provide the Investigator with dedicated, standardized sampling equipment (labels, needles, tubes), and a trial-specific laboratory manual, which will explain how to use the equipment and how to ship the samples back to the Central Laboratory. Results for hematology (except leucocytes and differential count after screening), chemistry, urinalysis, coagulation, thyroid function, and pregnancy tests will be provided by fax to the Investigator within 24 hours after sample receipt.

At visits where 12-lead ECGs will be performed, blood sampling should be done after recording the ECG.

The following laboratory assessments will be conducted:

- Blood chemistry (fasting): glucose, urea, creatinine, sodium, potassium, calcium, magnesium, chloride, total serum protein, albumin, bilirubin (total, conjugated and unconjugated), alkaline phosphatase (AP), aspartate aminotransferase (ASAT or SGOT), alanine aminotransferase (ALAT or SGPT), gamma-glutamyltranspeptidase (γGT), uric acid, total cholesterol: V1, V3 (day 1 pre-dose), V4, V6 (day 28).
- The creatinine clearance (Cr Cl) will be calculated by the Cockroft's formula :
    - male : Cr Cl ml/min= [(140-age) x body weight] / [72 x serum creatinine (mg/dl)],
    - female: Cr Cl ml/min=[(140-age) x body weight] / [72 x serum creatinine (mg/dl)] x 0.85.
- The eGFR will be calculated using the ' Modification of Diet in Renal Disease' study (MDRD) formula [(10)].
- Hematology: erythrocytes, thrombocytes, hemoglobin, hematocrit, mean corpuscular volume (MCV), mean corpuscular hemoglobin (MCH), mean corpuscular hemoglobin concentration (MCHC): at V1, V3 (day 1 pre-dose), V4 and V6 (day 28),
- Total and differential white blood cell count: at V1, V3 (day 1 pre-dose), V4 and V6 (day 28);
- Lymphocyte phenotyping: T-cells (CD3, CD4, CD8), B-cells (CD19, CD20, CD27 subsets), monocytes (CD14), hematopoietic progenitor cells (CD34), NK cells (CD56), NKT cells (CD3, CD56) and T_{reg} cells (CD4+CD25+FoxP3+), together with α4 expression for each subset., VCAM-1- inhibition: at V1, V3 (day 1 pre-dose), V5, V6 (day 28), V7, V8, V9, V10;
- Urinalysis: pH, protein, glucose, erythrocytes, leukocytes: V1, V6 (day 28);
- Thyroid function test: thryroid-stimulating hormone (TSH): V1, V6 (day 28);

- Coagulation: partial thromboplastin time and prothrombin time: V1, V3 (day 1 pre-dose), V6 (day 28);
- Pregnancy test: to be performed in women with childbearing potential: ßHCG blood test at V1 and urine tests at V3 (day -1) & V6 (day 27);
- Markers for infectious diseases:
  - HIV1 and HIV2 (HIV1/2 antibodies): V1, V6 (day 28);
  - Hepatitis B and C (HBsAg HBV-DNA, HCV-RNA): V1, V6 (day 28);
  - Tuberculosis (TSPOT-TB assay): V1, V6 (day 28);
  - Toxoplasmosis: V1, V6 (day 28);
- JC virus DNA in serum and/or PBMCs: V1, V2, V6 (day 28).

If a potential AE requires results of specific laboratory parameters faster than those analyzed in the Central Laboratory, some parameters may have to be determined in a local laboratory in advance or in parallel. Those analyses may serve as guidance for the Investigator in determining particular requirements for care for a research participant. They do not constitute study data in the sense of this protocol. The only study data in the sense of this protocol are the centrally obtained laboratory results. However, should a given subject suffer from a serious adverse event (SAE), laboratory results from locally analyzed samples will become part of the relevant safety information during SAE reporting.

Table 11:1 Overview about the Blood Sampling Schedule Including Volumes Per Visit (in mL)

<table>
<tr><th></th><th>V1</th><th>V2</th><th>V3</th><th>V4</th><th>V5</th><th>V6</th><th>V7</th><th>V8</th><th>V9</th><th>V10</th></tr>
<tr><td>Blood Chemistry</td><td rowspan="2">3.5</td><td></td><td>3.5</td><td>3.5</td><td></td><td>3.5</td><td></td><td></td><td></td><td></td></tr>
<tr><td>Pregnancy</td><td></td><td></td><td></td><td></td><td></td><td></td><td></td><td></td><td></td></tr>
<tr><td>Thyroid Function</td><td rowspan="4">17</td><td></td><td></td><td></td><td></td><td rowspan="4">17</td><td></td><td></td><td></td><td></td></tr>
<tr><td>HIV</td><td></td><td></td><td></td><td></td><td></td><td></td><td></td><td></td></tr>
<tr><td>Hepatitis</td><td></td><td></td><td></td><td></td><td></td><td></td><td></td><td></td></tr>
<tr><td>Toxoplasmosis</td><td></td><td></td><td></td><td></td><td></td><td></td><td></td><td></td></tr>
<tr><td>JC Virology</td><td>8.5</td><td>8.5</td><td></td><td></td><td></td><td>8.5</td><td></td><td></td><td></td><td></td></tr>
<tr><td>JC Virology PBMC</td><td>8</td><td>8</td><td></td><td></td><td></td><td>8</td><td></td><td></td><td></td><td></td></tr>
<tr><td>Hematology</td><td rowspan="2">4</td><td></td><td>4</td><td>4</td><td></td><td>4</td><td></td><td></td><td></td><td></td></tr>
<tr><td>Leucocytes and differential count</td><td></td><td>4</td><td>4</td><td></td><td>4</td><td></td><td></td><td></td><td></td></tr>
<tr><td>Tuberculosis</td><td>10</td><td></td><td></td><td></td><td></td><td>10</td><td></td><td></td><td></td><td></td></tr>
<tr><td>Coagulation</td><td>4.5</td><td></td><td>4.5</td><td></td><td></td><td>4.5</td><td></td><td></td><td></td><td></td></tr>
<tr><td>Lymphocyte Phenotyping</td><td>4</td><td></td><td>52</td><td></td><td>4</td><td>28</td><td>4</td><td>4</td><td>4</td><td>4</td></tr>
<tr><td>Pharmacokinetics</td><td></td><td></td><td>14</td><td></td><td>2</td><td>14</td><td>2</td><td>2</td><td>2</td><td>2</td></tr>
<tr><td>Volume per Visit</td><td>59.5</td><td>16.5</td><td>82</td><td>11.5</td><td>6</td><td>101.5</td><td>6</td><td>6</td><td>6</td><td>6</td></tr>
</table>

The overall blood volume taken for subjects completing the regular treatment visits for the study will be 301 mL.
All laboratory analyses will be performed at The Doctors Laboratory Ltd. (London, UK) with the exception of the following analyses:

- T-Spot TB, performed by Oxford Immunotec (Oxford, UK)
- JC virus, the laboratory name and procedures will be described in the laboratory manual
- PD parameters of lymphocyte phenotyping: the laboratory name and procedures will be described in the laboratory manual
- PK parameters performed by UCB Braine.

11.1.10 Electrocardiogram (ECG)

A standard 12-lead ECG (six limb leads: I, II, III, aVR, aVL, aVF, six chest leads: V1-V6) will be obtained at Screening (V1), V3 (day -1, day 1 pre-dose, 30 min and 2.5 h post-dose and day 5), and V6 (day 28). ECGs will be electronically recorded for 1 minute at a speed of 25 mm/s and with a calibration of 1 cm/mV. The Investigator will determine whether the results of the ECG are normal or abnormal. For the purpose of measuring QTcF, the Investigator may rely on the computed output of the ECG machine. In case of doubt, a local manual overread by a cardiologist should be sought. The results of the manual overread will overrule any computed output. In case of a suspected prolongation of QTcF beyond the protocol-specified ranges, triple repeat ECGs will be obtained and read centrally. A decision on continuation of treatment with study drug will be made thereafter.

A sample of all ECG tracing will be printed, signed or initialed and dated by the Investigator and retained as part of the Investigator's CRF copy. Further, all ECGs will be centrally interpreted in the Division of Cardiac and Vascular Sciences, St George's Hospital (London, UK). For this purpose, an electronic copy of the tracing will be made available. An electronic back-up copy will be stored on site. .

The locally measured or calculated ECG variables are used for on-site safety decision-making. They will not be databased, nor analyzed. Analyzable study data in the sense of this protocol are the central ECG readings described above.
However, should a given patient suffer from a SAE, results from locally read ECGs will become part of the relevant safety information during SAE reporting.

At visits where a 12-lead ECG has to be performed, this should be done before any blood sampling.

At V3, a continuous cardiac telemetry with arrhythmia detection will be performed from 12 hours pre-dose to 36 hours post-dose or longer if necessary, as judged by the Sponsor or Investigator.

11.1.11 Magnetic Resonance Imaging (MRI)

All subjects will undergo contrast-enhanced MRI scanning according to a separate MRI manual. A MRI scan will be performed at V1 and V6 after i.v. administration of 0.1 mmol gadolinum (Gd) using one of these two agents: gadoteridol (Prohance®) or gadobutrol (Gadovist®). The contrast agent will be provided under the responsibility of the local radiology facility.

Gadolinium (Gd) is considered a safe contrast agent. It may occasionally cause nausea and vomiting. Very rarely, it may cause warmth and pain at the injection site. Allergic reactions may also occur very rarely and can – in extremely rare instances – be potentially serious (anaphylactic shock in 55 of 20 billion applications). Recently, the occurrence of a new disease entity, the nephrogenic systemic fibrosis (NSF), has been described after the administration of Gd-containing contrast agents. The occurrence of NSF has been observed in patients with severely impaired renal function and in patients having received organ transplants. Current knowledge indicated also that the physico-chemical properties of the contrast agent may play a role in the occurrence of NSF. The agents selected for administration in this study are cyclic Gd chelates. It is believed that they are less likely to release the toxic Gd^{3+} ions. They have so far not been associated with the occurrence of NSF. (11)

All MRI scans will be read by a local reader and by a Central MRI Reading Unit (CRU-MRI). The local reading will encompass:

- for the screening MRI:
  - conformity to the diagnosis of MS and
  - evaluation for non-MS pathology including exclusion of features suspect of PML
- for the safety MRI (V6, day 28)
  - safety evaluation regarding potentially emerging features suspect of PML.
  - evaluation for non-MS pathology

After the local reading, all scans will be sent to the CRU-MRI. During the study, the quality of each scan performed will be assessed at the CRU-MRI. The MRI scans should be sent as electronic data to the CRU-MRI within 24 hours after acquisition. As soon as the scan is received by the CRU-MRI, it will be evaluated for quality and completeness. The central reading will encompass

- for the screening MRI:
  - conformity to the diagnosis of MS,
  - description of MS pathology, and
  - exclusion of features suspect of PML;
- and blinded for the safety MRI (V6, day 28)
  - safety evaluation regarding potentially emerging features suspect of PML and.
  - description of MS pathology.

The study site will be informed about rejection or acceptance of a specific scan at the latest within five days after receipt of scan (in general, it is assumed that most reasons for rejection will be identified within one to three days; however, under rare circumstances, a five-day period may be necessary). If a scan is not accepted by the CRU-MRI, a repeat scan has to be performed as early as possible. The time between scan and re-scan must not exceed 14 days.

To preserve subject confidentiality, the full name of the subject must not appear on the images. The scans should contain the following identifiers: subject number, subject's initials, study site number, date of investigation and visit. A comment line should specify the name and the volume of contrast material delivered. The results of the MRI evaluation will be sent to UCB's Data Management on signed printouts as well as in electronic form. The electronic data obtained during the scanning process will be stored both at the CRU-MRI and at the respective study site.

A radiologist and a technician from each study site will be invited to attend an MRI training session covering issues related to technical implementation and image quality assurance.

11.1.12 Pharmacokinetic and Pharmacodynamic Sampling

PK sampling will occur over 0-24 hr after the first morning dose, pre-dose at day 15 and over 0-168 hr after the last dose. The last dose should always be given as a morning dose. Sampling for PD assessments will occur during most PK sampling timepoints.

In order to maintain the blind, PD data (including all leukocyte-related parameters from hematological tests) will not be made available to the study center personnel until after study unblinding, with the exception of leukocyte levels above 15000 x 10^9/l or below 500 x 10^9/l and CD4+ counts below 200 x 10^9/l.

Date and time of PK and PD sampling will be recorded on the transmittal form as well as on the CRF. Also date and time of last two intakes of study medication and date and time of last meal prior to sampling will be recorded on the CRF.

The clinical center will be provided with a Laboratory Manual which will provide detailed descriptions of collection, preparation, storage and labeling/shipping requirements for all blood samples for PK and PD (biomarkers), including a description of the laboratory kits to be used for each sample.

A detailed PK/PD sampling plan is outlined in Table 11:2.

Table 11:2 PK/PD Sampling Schedule

Sampling Timepoints				PK	VCAM1 Binding	Leukocyte Parameters
Day	Visit	Timepoints	Time			
-21	V1	Baseline			x	x
-1	V3	Baseline	08:00		x	x
			09:00			x
			10:00			x
			12:00			x
			16:00			x
			20:00[(c)]			x
1	V3	Pre-dose	08:00[(b)]	x	x	x
		1h pd[(a)]	09:00	x	x	x
		2h pd[(a)]	10:00	x	x	x
		4h pd[(a)]	12:00	x	x	x
		8h pd[(a)]	16:00	x	x	x
		12h pd[(a)]	20:00[(c)]	x	x	x
2	V3	24h pd[(a)]	08:00[(b)]	x	x	x
15	V5	Pre-dose	08:00[(b)]	x	x	x
28	V6	Pre-dose	08:00[(b)]	x	x	x
		1h pd[(a)]	09:00	x	x	x
		2h pd[(a)]	10:00	x	x	x
		4h pd[(a)]	12:00	x	x	x
		8h pd[(a)]	16:00	x	x	x
		12h pd[(a)]	20:00	x	x	x
29	V6	24h pd[(a)]	08:00	x	x	x
30	V7	48h pd[(a)]	08:00	x	x	x
31	V8	72h pd[(a)]	08:00	x	x	x
32	V9	96h pd[(a)]	08:00	x	x	x
35	V10	168h pd[(a)]	08:00	x	x	x

[(a)] pd = post dose
[(b)] before morning dose
[(c)] before evening dose
[(d)] only the QD regimen samples analyzed

Per sampling time point, 2 mL of blood volume will be drawn for the pharmacokinetic samples (N=19) and 4 mL for PD assessments (N=26).

11.1.13 Neuro Exam and EDSS

The assessment of the Expanded Disability Status Scale (EDSS; Kurtzke 1983) will be performed at Screening (V1), V6 (Day 28), V11 and V12. The EDSS assessment is based on the standardized neurological examination and the Kurtzke Functional Systems. The neurological examination comprises the check of optic, brain stem/cranial nerves, pyramidal,

cerebellar, sensory, vegetative, and cerebral functions, as well as ambulation, from which Functional Scores will be determined. Details are given on the respective CRF and in Appendix 17.1.

11.1.14 Assessment of New Neurological Events

Particular attention has to be paid to the occurrence of new neurological abnormalities ('new neurological event' - NNE) during the trial period from V1 to V12. Subjects will be instructed to contact the study site within 24 hours should any symptoms suggestive of a new neurological event occur. The Investigator should evaluate the subject as early as possible within the subsequent three days by doing a full neurological examination and assessing the EDSS. The assessment of a new neurological event has to be documented. Based on the neurological examination the Investigator will decide whether the new neurological event may constitute a relapse according to the definition below or may justify an evaluation for PML (see Section 11.1.15).

A relapse is the appearance of a new neurological abnormality or the reappearance of a neurological abnormality, separated by at least 30 days from the onset of a preceding neurological event. The abnormality must be present for at least 24 hours and occur in the absence of fever and known infection. Fever is defined as a body temperature measured either axillary, orally, or intrauricularly of > 37.5°C or 99.5°F. Objective neurological impairment must correlate with the subject's reported symptoms and must fulfill any of the below criteria:

- increase in more than one of the functional systems of the EDSS or increase of the total EDSS score
- not restricted to either paresthesia, fatigue, mental or vegetative symptoms without any additional neurological symptom

The evaluation of a new neurological event should not be completed in the presence of fever or infection. The EDSS progression assessment must be repeated after fever or infection have ceased. However, clinical judgment should be exercised whether an assessment for PML may be justified.

Only Investigator-verified relapses will be considered as valid (including those already verified by other physicians, if appropriately documented in the subject's file). Follow-up visits to monitor the course of the relapse will be arranged at the Investigator's discretion.

In case of a relapse requiring steroid treatment, methylprednisolone iv 500 - 1,000 mg/d may be given over three to five days (if indicated, ranitidine 150 mg may additionally be given at night during the treatment cycle). Treatment with study medication has to be interrupted during steroid treatment. Relapses need to be documented on the AE form, steroid treatment on the Concomitant Medication form.

11.1.15 Surveillance for PML and Diagnostic Work-Up

In this study, clinical vigilance and/or the work-up of NNEs will be of eminent importance for the surveillance of PML. The presenting symptoms of PML typically affect cognitive, visual and motor function. In subjects with MS, they could mimic a MS relapse. Therefore, all NNEs involving the above mentioned functions and being reasonably suspicious of PML should undergo evaluation for PML. Table 11:3 gives an overview about differential diagnostic features.

Table 11:3 Differential Diagnosis between MS and PML: Clinical Considerations

	**MS Exacerbation**	**PML**
**Onset**	• acute	• subacute
**Evolution**	• over hours to days	• over weeks
	• normally stabilizes<br>• resolves spontaneously or when treated	• progressive
**Clinical Presentation**	• diplopia<br>• paraesthesia<br>• paraparesis<br>• optic neuritis	• cortical signs and symptoms<br>• behavioral & neuropsychological changes<br>• hemiparesis<br>• retrochiasmal visual deficits
	• myelopathy	

A PML Alert form will be faxed by the Investigator to UCB's Study Manager upon the identification of any subject having NNEs atypical for MS or events suspect of PML. UCB's Study Manager will inform the SAB immediately. In case of symptoms atypical for MS or suspect of PML, all of the following evaluations have to be performed:

- contrast-enhanced MRI with initial local reading for signs of PML followed by central safety reading;
- sampling of CSF and analysis for JCV DNA;
- sampling of blood for analysis of presence of JCV DNA in serum and PBMCs and a repeat sampling after seven days.

Further measures include the awareness of the subject, of persons close to the subject and of health-care professionals caring for the subject. Therefore, the subject will be made aware of typical signs and symptoms that would necessitate a diagnostic work-up for PML. The subject will also be asked to inform persons close to her/him, e.g., persons living in the same household. The family doctor and/or any other health care professional named by the subject will be informed by letter about their trial participation and will be made aware of typical signs and symptoms that would necessitate a diagnostic work-up for PML. The subject will further receive an Alert Card.

A subject will be considered to suffer from PML if the following condition is met:

- progressively worsening clinical symptomatology suggestive of PML with detectable JCV-DNA in the CSF.

An interim diagnosis of "being at high risk for developing PML" will be considered if one of the following conditions is met:

- Progressively worsening clinical symptomatology suggestive of PML with a duration of at least 14 days,
- MRI findings suggestive of PML,
- a positive testing for circulating JCV-DNA in either serum or PBMCs on two consecutive occasions or in CSF.

Dosing will be terminated for all subjects suffering from one of the above conditions, be it a diagnosis of PML or an interim diagnosis of "being at high risk for developing PML". Though this study stipulates the termination of study drug treatment should the above mentioned conditions occur, the interim diagnosis of "being at high risk for developing PML" is not a diagnosis of PML. The investigator is advised to undertake the clinically accepted surveillance to either rule out or establish a diagnosis of PML.

Subjects in need for steroid treatment for an MS exacerbation can receive steroids if the local neuroradiologist reports that the "MRI findings do not support a diagnosis of PML". Steroid treatment should be postponed for subjects for whom "MRI findings supporting a diagnosis of PML" have been reported.

If a subject has been diagnosed with PML or been classified as "being at high risk for developing PML", the withdrawal of the study medication should allow immunoreconstitution. It is recognized that there is currently no causal treatment beyond immunoreconstitution. The investigator is advised to initiate the needed symptomatic treatment and care. If their clinical condition allows so, such subjects will be encouraged to undergo all remaining study visits as planned.

In the case of a confirmed diagnosis of PML in a CDP323-treated subject, dosing of all subjects will be discontinued, but clinical evaluations will be completed.

11.1.16 Concurrent Medical Procedures

From V1 until V10, collection of data on medical procedures (surgery, therapeutic and/or diagnostic, hospitalizations) undertaken during the trial will be obtained. ECGs and or MRI scans specific to this trial will **not** be recorded on the Medical Procedures page of the CRF but in the modules specifically designed for this purpose.

11.1.17 Concomitant Medication

All medication (including symptomatic MS treatment and over-the-counter preparations) taken within the 30 days prior to the first visit and during the course of the trial until V10 must be documented in the CRF (brand name, indication, dosage, and the dates of start and discontinuation). At each visit, a complete listing of all medications currently being taken will be obtained. Any changes, additions, or deletions in the administration of concomitant medication must be recorded on the Concomitant Medications page of the CRF. In case of intake of prohibited concomitant medication (cf. Section 10.2.3) during the trial period, the Investigator will contact the monitor immediately and will withdraw the study medication. For subjects withdrawing from the trial and not willing to complete the drug-free follow-up, the Investigator is allowed to start any MS treatment 30 days after termination of the study medication.

11.1.18 Adverse Events (AEs)

From V1 to V10, AEs will be assessed at each visit and recorded in the source documents and in the CRF. The subject will be given the opportunity to report AEs spontaneously. A general prompt will also be given to detect AEs, e.g., "Did you notice anything unusual about your health (since your last visit)?" Conditions known at trial entry should be recorded in medical history and/or physical examination, if applicable.

During the 12 month follow-up period, only SAEs of the following type will be recorded and reported: PML, serious infections (opportunistic infections, infections requiring parenteral or intra-muscular antibiotherapy and Tuberculosis cases), malignancies.

11.1.19 Drug Administration

Study drug (and/or placebo) are administered for 28 consecutive days. Subjects have to take 6 capsules in the morning and 6 capsules in the evening, ideally 12 hours apart. Should the subject forget intake of any given dose, the dose can be taken within six hours of the originally planned time of intake. If the omission was noted more than six hours after the originally planned dose, this dose should be left out and the next dose should be taken according to schedule.

Capsules should be taken with a meal or within 30 minutes after a meal. During the confinement period, subjects will be instructed to take the study drug 5 min after their meals (which have to be eaten within 30 min). The days when subjects are at the clinical center, the capsules will be taken under the supervision of the Investigator. During all other days, the responsibility lies with the subject.

11.1.20 Confinement

In order to prudently manage any unexpected untoward events, all subjects will be hospitalized for the first five days (Day -1 till Day 5) during the treatment period. The Investigator will give clear instructions to the subject as to when he/she is expected to arrive and the subject can only leave upon approval of the Investigator. Further hospitalization will happen from day 27 evening until day 29 morning to logistically support PK/PD sampling during visit 6 with intense sampling.

11.1.21 End of Treatment Phase and End of Drug-free Follow-up

The end of the subject's participation in the trial will be confirmed on the subject's status evaluation section of the CRF. There will be a subject's status evaluation section at the end of the treatment phase (V6) and one at the end of the drug-free follow-up (V12). All data about the subject's status (completion or reason for early trial termination) will be recorded at both visits. It will be specified:

- Whether the subject completed or discontinued the treatment phase/drug-free follow-up
- Whether the subject is willing to undergo the non-interventional follow-up after 3 and 12 months.
- Whether the subject is willing to enter the planned open long-term safety study once it becomes available.

A subject will be considered lost to follow up after two unsuccessful attempts to contact the subject (e.g. by telephone). These attempts should be documented.

11.2 Description Visit by Visit

11.2.1 Visit 1 (Day -21) Screening

Prior to any study activities, subjects will be asked to read and sign an informed consent form that has been approved by an Independent Ethics Committee (IEC) and which complies with regulatory requirements. Subjects will be given adequate time to consider any information concerning the study, given to them by the Investigator or designee. As part of the informed consent procedure, subjects will be given the opportunity to ask the Investigator any questions regarding potential risks and benefits of participation in the study.

The Investigator will in particular discuss the requirements for the drug-specific wash-out times (see Section 9.2) and the potential risks associated with the participation in this study. The subject will be made aware of typical signs and symptoms that would necessitate a diagnostic work-up for PML. The subject will also be asked to inform persons close to her/him, e.g., persons living in the same household. Subjects not consenting to this will not be admitted to the trial.

Further, the subject will be made aware that the family doctor and/or any other health care professional named by the subject will be informed by letter about their trial participation. Subjects not consenting to this will not be admitted to the trial. In addition, subjects will also be made aware that the required safety surveillance may necessitate a lumbar puncture to sample CSF. Only subjects consenting to the procedure at study start will be admitted to the trial (cave: the initial consent will not prevent the subject from refusing the procedure when actually asked to undergo CSF sampling).

The following assessments and investigations will be conducted during Visit 1:

- Written informed consent
- Demographic data
- Inclusion/exclusion criteria
- Medical and Procedures history
- MS history and diagnosis
- MS medication history
- Vital signs
- Physical examination (including weight and height)
- Childbearing potential & birth control + Pregnancy test (ßHCG blood test for women of childbearing potential)
- Blood chemistry, hematology, lymphocyte phenotyping
- Coagulation, thyroid function
- Urinalysis
- JC virology
- Toxoplasmosis, tuberculosis
- HIV, hepatitis
- 12-lead ECG and calculation of the Screening QTcF
- MRI scan
- Neurological examination including EDSS
- Assessment of New Neurological Events
- Concurrent medical procedures
- Concomitant medications, including the last 30 days before V1
- Recording of AEs after signing the written informed consent
- Appointment for the next visit.

11.2.2 Visit 2 (Day -14) Screening

The following assessments and investigations will be conducted during Visit 2:

- Inclusion/exclusion criteria
- Withdrawal criteria
- JC virology

- Assessment of New Neurological Events
- Concurrent medical procedures
- Concomitant medications
- Recording of AEs
- Appointment for the next visit.

11.2.3 Visit 3

The following assessments and investigations will be conducted during Visit 3:

Day -1
- Inclusion/exclusion criteria
- Withdrawal criteria
- Childbearing potential & birth control/pregnancy test (urine test for women with childbearing potential)
- 12-lead ECG and calculation of the Baseline QTcF
- Start Confinement
- PD blood samplings
- Assessment of New Neurological Events
- Concurrent medical procedures
- Concomitant medications
- Recording of AEs
- Start Telemetry 12h pre-dose

Day 1
- Withdrawal criteria
- Vital signs (30 min pre-dose, 30 min post-dose, 2.5 hr post-dose)
- Physical examination
- Childbearing potential & Birth Control
- Blood chemistry, hematology: pre-dose
- Coagulation
- PK/PD blood sampling (see Table 11:2)
- Dispensation of "Subject Trial Card" documenting the participation in the trial and “Subject Alert Card” containing the information about investigational product.
- Randomization
- Drug administration (from blister of week 1)
- telemetry (12 hr pre-dose till 36 hr post-dose)
- Assessment of New Neurological Events
- Concurrent medical procedures
- Concomitant medications
- Recording of AEs

- Confinement

Day 2

- Withdrawal criteria
- Drug administration
- telemetry (till 36 hr post-dose)
- Assessment of New Neurological Events
- Concurrent medical procedures
- Concomitant medications
- Recording of AEs
- PK/PD blood sampling (see Table 11:2)

Drug administration, assessment of new neurological events, check of withdrawal criteria, concurrent medical procedures, concomitant medications and AEs recording as well as confinement will continue until day 5.

Day 5

- Withdrawal criteria
- Vital signs
- Childbearing potential & Birth Control
- 12-lead ECG
- Drug administration
- Assessment of New Neurological Events
- Concurrent medical procedures
- Concomitant medications
- Recording of AEs
- End of Confinement
- Handing over of medication blister of week 1
- Appointment for the next visit.

11.2.4 Visit 4 (Day 8) Treatment Period

The following assessments and investigations will be conducted during Visit 4:

- Withdrawal criteria
- Vital signs
- Childbearing potential & Birth Control
- Blood chemistry, hematology
- Breakfast and drug administration on site (first dose of blister for week 2)
- Assessment of New Neurological Events
- Concurrent medical procedures
- Concomitant medications
- Recording of AEs

- Check of compliance
- Appointment for the next visit.

11.2.5 Visit 5 (Day 15) Treatment Period

The following assessments and investigations will be conducted during Visit 5:

- Withdrawal criteria
- Vital signs
- Physical Examination
- Childbearing potential & Birth Control
- PK/PD blood sampling
- Breakfast and drug administration on site (first dose of blister for week 3)
- Assessment of New Neurological Events
- Concurrent medical procedures
- Concomitant medications
- Recording of AEs
- Handing over of blisters for weeks 3 and 4
- Appointment for the next visit.

11.2.6 Visit 6

The following assessments and investigations will be conducted during Visit 6:

Day 27 evening

- Withdrawal criteria
- Childbearing potential & Birth Control + pregnancy test (urine test for women with childbearing potential)
- Assessment of New Neurological Events
- Concurrent medical procedures
- Concomitant medications
- Recording of AEs
- Confinement
- Drug administration

Day 28

- Withdrawal criteria
- Vital signs
- Physical examination (including weight)
- Blood chemistry, hematology
- Coagulation, thyroid function
- Urinalysis

- JC Virology
- Tuberculosis, toxoplasmosis
- HIV, hepatitis
- 12-lead ECG
- MRI scan
- PK/PD blood sampling (see Table 11:2)
- Neurological exam & EDSS
- Drug administration : last dose of the study = morning dose of day 7 from blister of week 4
- Assessment of New Neurological Events
- Concurrent medical procedures
- Concomitant medications
- Recording of AEs
- Confinement

Day 29
- PK/PD blood sampling (see Table 11:2)
- Assessment of New Neurological Events
- Concurrent medical procedures
- Concomitant medications
- Recording of AEs
- End of confinement
- Appointment for the next visit

11.2.7 Visit 7 (Day 30)

- Vital signs
- PK/PD blood sampling (see Table 11:2)
- Assessment of New Neurological Events
- Concurrent medical procedures
- Concomitant medications
- Recording of AEs
- Appointment for the next visit.

11.2.8 Visit 8 (Day 31)

- Vital signs
- PK/PD blood sampling (see Table 11:2)
- Assessment of New Neurological Events
- Concurrent medical procedures
- Concomitant medications

- Recording of AEs
- Appointment for the next visit.

11.2.9 Visit 9 (Day 32)

- Vital signs
- PK/PD blood sampling (see Table 11:2)
- Assessment of New Neurological Events
- Concurrent medical procedures
- Concomitant medications
- Recording of AEs
- Appointment for the next visit.

11.2.10 Visit 10 (Day 35)

- Vital signs
- PK/PD blood sampling (see Table 11:2)
- Assessment of New Neurological Events
- Concurrent medical procedures
- Concomitant medications
- Recording of AEs
- Subject's status evaluation
- Appointment for the next visit.

11.2.11 Visit 11 (+3 months) Follow-up

The 3-month follow-up visit may be carried out by telephone or during a visit

- Vital signs
- Physical examination
- EDSS (can be done over the phone)
- Assessment of New Neurological Events
- SAEs of following types: PML, serious infections (opportunistic infections, infections requiring parenteral or intra-muscular antibiotherapy and Tuberculosis cases), malignancies.
- Appointment for the next visit.
- Discussion of the possibility to participate in an open long-term safety study once it will become available.

11.2.12 Visit 12 (+12 months) Follow-up

- Vital signs
- Physical examination (including vital signs and weight)

- Neurological examination and EDSS
- Assessment of New Neurological Events
- SAEs of following types: PML, serious infections (opportunistic infections, infections requiring parenteral or intra-muscular antibiotherapy and Tuberculosis cases), malignancies.
- Discussion of the possibility to participate in an open long-term safety study once it will become available.
- Subject's status evaluation.

11.2.13 Unscheduled Contact with the Study Site

Unscheduled contact may occur for any reason and at any time. Unscheduled contacts may include e.g., visits, phone calls, letters, or e-mails. The date, form of unscheduled contact, and reason will be recorded on the Unscheduled Contact form. If the subject is hospitalized, the SAE form and hospitalization page must be completed. If a subject reports a new neurological event over the phone she/he will be advised to visit the study center within the subsequent three days.

12. ADVERSE EVENTS AND SERIOUS ADVERSE EVENTS

12.1 Adverse Events

12.1.1 Definition of Adverse Event (AE)

An adverse event is any untoward medical occurrence in a patient or clinical investigation subject administered a pharmaceutical product which does not necessarily have a causal relationship with this treatment. An adverse event can therefore be any unfavorable and unintended sign (including an abnormal laboratory finding), symptom, or disease temporally associated with the use of a medicinal (investigational) product, whether or not related to the medicinal (investigational) product.

In order to ensure complete safety data collection, all AEs or undesirable experiences occurring during the study (i.e., after signature of the Informed Consent), including any pre- and post-treatment periods required by the protocol, must be reported in the CRF even if no investigational product was taken but specific study procedures were conducted. These include all AEs not present prior to the initial visit and all AEs which recurred or worsened after the initial visit.

Signs or symptoms of the condition/disease for which the investigational product is being studied should be recorded as AEs only if their nature changes considerably or their frequency or intensity increases in a clinically significant manner as compared to the clinical profile known to the Investigator from the subject's history or the baseline period.

12.1.2 Procedures for Reporting and Recording Adverse Events

The study participant will be given the opportunity to report Adverse Events spontaneously. A general prompt will also be given to detect adverse events, e.g.

Did you notice anything unusual about your health (since your last visit)?

In addition, the Investigator should review any self-assessment procedures (e.g., diary cards) employed in the study.

12.1.3 Description of AEs

The following guidelines and definitions should be used by the Investigator for the description of an AE when reporting information:

Nature of the AE:	Preferably an overall diagnosis or syndrome, rather than individual symptoms or signs. The Investigator must report adverse events using standard medical terminology. The CRF and source documents should be consistent. Any discrepancies between the subject's own words on his/her own records (e.g., diary card) and the corresponding medical terminology should be clarified in the source documentation.
Date of onset (and time):	Date (and time) the AE started.
Pattern:	
Intermittent	The AE recurs with the same intensity at various intervals throughout the entire time period specified. There were intervals within the specified time period when the AE was not present.
Continuous	The AE is present at the same intensity for the entire time period specified. There was no time at which the event abated or was not present during the time period specified.
Intensity:	
Mild	The subject is aware of the sign or symptom, but it does not interfere with his/her usual activities and/or it is of no clinical consequence.
Moderate	The AE interferes with the usual activities of the subject or it is of some clinical consequence.
Severe	The subject is unable to work normally or to carry out his/her usual activities, or the AE is of definite clinical consequence.

	**Note** : All AEs Grade 4 according to Common Terminology Criteria for Adverse Events (CTCAE) v3.0 are considered 'serious', including deviation from reference ranges in laboratory tests.
Actions taken with investigational product:	
Not applicable	For AEs occurring during the investigational product free period (pre and post-treatment periods and for single dose studies).
No change	Investigational product dosing remained the same in spite of the AE being present.
Dose increased	Investigational product dose was increased because of this AE.
Dose decreased	Investigational product dose was decreased because of this AE.
Temporarily discontinued	Investigational product was temporarily discontinued because of this AE, either because the subject chose to discontinue the investigational product or the physician felt it was in the subject's best interest to temporarily discontinue the investigational product.
Permanently discontinued	Investigational product was permanently discontinued because of this AE, either because the subject chose to discontinue the investigational product or the physician felt it was in the subject's best interest to discontinue the investigational product.
Other actions taken	
None	No other action was taken for this AE.
Medication	The subject took a medication (either prescription or non-prescription) specifically for this AE or existing medication dosage was modified.
Hospitalization or prolongation of hospitalization	Subject was hospitalized for this AE or subject's stay in hospital was prolonged because of this AE.
Therapeutic or diagnostic procedure	Subject used other therapeutic measures (e.g. ice, heating pad, brace, cast, etc.) or subject underwent a diagnostic procedure (e.g., additional lab test, x-ray, etc.) for this AE.
Date of outcome (and time):	Date (and time) the AE abated. If the AE consists of several signs and symptoms, the sign or symptom with the longest duration determines the duration of the AE.
Outcome:	
Resolved	The AE is no longer present at any intensity -

	completely abated.
Resolved with sequelae	The AE is resolved but residual effects are still present.
Ongoing	The AE is still present at the last contact with the subject.
Worsened	The AE is still present but at a heightened intensity. The rule of repetition of AE reporting should be applied.
Fatal	This AE caused or directly contributed to the subject's death.
Relationship to investigational product:	
None	Only applicable when no investigational product was taken or when the subject is taking single-blind placebo, or when the AE can be ascribed with reasonable certainty to another cause.
Unlikely	There are good reasons to think that there is no relationship e.g., the AE is a known adverse drug reaction of a concomitant medication, *or the same AE does not reappear after re-administration of the investigational product.*
Possible	Equally valid arguments can be considered for or against an implication of the investigational product, For example, the AE:<br>• follows a reasonable temporal sequence from the administration of the investigational product;<br>• follows a known or expected response pattern to the investigational product;<br>• but could readily have been produced by a number of other factors.
Probable	The relationship is likely. For example, the AE:<br>• follows a reasonable temporal sequence from administration of the investigational product;<br>• follows a known or expected response pattern to the investigational product;<br>• is confirmed by improvement on stopping or reducing the dosage of the investigational product;<br>• could not be reasonably explained by the known characteristics of the subject's clinical state.
Highly probable	There is a strong relationship. For example, the AE:<br>• follows a reasonable temporal sequence from administration of the investigational product or

	in which the investigational product level has been established in body fluids or tissues;<br>• follows a known or expected response pattern to the investigational product;<br>• is confirmed by improvement on stopping or reducing the dosage of the investigational product, and reappearance on repeated exposure (rechallenge).

12.1.4 Follow-up on Adverse Events

If an AE is still ongoing at the end of the study for a subject, a follow-up report should be provided until resolution/stable level of sequelae or the Investigator no longer feels it is clinically significant. If no follow-up report is provided, the Investigator must provide a justification. The follow-up will be usually continued for 30 days after the subject has completed the study or until the pre-analysis meeting, whatever is shorter.

UCB may request that the Investigator perform or arrange for the conduct of supplementary measurements and/or evaluations.

12.1.5 Rule for Repetition of an AE

An increase in the intensity of an AE should lead to the repetition of the AE reporting with:

- the outcome date of the first AE that is not related to the natural course of the disease being the same as the start date of the repeated AE,
- the AE verbatim being the same for the first and repeated AE, so that the repeated AE can be easily identified as the worsening of the first one.

12.1.6 Pregnancy

Should a subject become pregnant after the first intake of any investigational product, UCB should be informed immediately. The subject should be excluded from the study as soon as pregnancy is known (e.g. immediate stop of investigational product intake, immediate start of down titration, …). The investigator must inform the subject about the potential risk of malformations and about the available alternatives, e.g. voluntary termination with medical indication (to be proposed on a study-specific basis).

The pregnancy will be documented in the AE section of the CRF. The progression of the pregnancy and the eventual birth (if applicable) must be followed up using UCB Investigator Pregnancy Report form in which the Investigator has to report on the health of the mother and of the offspring. The health of the child must be followed for 12 months after birth for any significant medical issues.

A pregnancy becomes an SAE in the following circumstances: miscarriage, abortion or anomaly/birth defect of the child. Those serious events must be additionally reported using the Investigator SAE report form.

It is potentially a SAE if it is the consequence of a birth control method failure.

12.1.7 Overdose of Investigational Product

CDP323 and its metabolites are of low acute toxicity with no adverse reactions in rat and mouse at limit or maximum practical doses. Therefore, overdosing of CDP323 in the clinical setting would be unlikely to result in acute toxicity. Doses up to 1000 mg twice daily (i.e., 2000 mg/d) have been investigated in human volunteers without significant AEs.

Two out of the six volunteers exposed to 1000 mg twice-daily over at least seven days experienced four AEs in total: headache, dizziness, fatigue, and hordeolum (each event occurred once).

Any dose beyond 2000 mg/d is considered overdose. Only symptomatic overdoses need to be recorded as AEs. These events may be symptomatic, in that, the excessive dosing results in clinical signs and symptoms or the excessive intake may itself be a symptom. Excessive dosing (beyond that prescribed and including overdose) should be recorded in the study medication module. In case of symptomatic overdose, the study medication should be withdrawn, the subject should be hospitalized, and symptomatic care should be given as needed. In case of a lacking symptomatology, dosing should proceed as originally planned.

12.2 Serious Adverse Events

12.2.1 Definition of Serious Adverse Event (SAE)

A Serious Adverse Event is any untoward medical occurrence that at any dose

- results in death,
- is life threatening,
- requires in-patient hospitalization or prolongation of existing hospitalization,
- results in persistent or significant disability/incapacity
- is a congenital anomaly/birth defect

In this context, the term life threatening refers to an event in which the subject was at immediate risk of death at the time of the event; it does NOT refer to an event which might have caused death if it would have been more severe.

Any important medical event that may not be immediately life threatening or result in death or hospitalization but, based upon appropriate medical judgment, may jeopardize the subject or may require medical or surgical intervention to prevent one of the other outcomes listed in

the definition above should also be reported as a SAE. Examples of such events are intensive treatment in an emergency room or at home for allergic bronchospasm, blood dyscrasias or convulsions that do not result in hospitalization; or development of drug dependency or drug abuse.

Cases of cancer should be reported as SAE following the criteria “Important medical event”.

Hospitalization criteria are met anytime a subject is formally admitted to hospital, regardless of duration, or if a subject is kept in hospital overnight and it is not clear if a formal admission took place. If a subject is taken to a hospital and neither of the above apply (e.g. an emergency room visit), an event may still be considered serious, meeting the criteria of medically important.

Hospitalization for diagnostic or therapeutic procedures in the absence of any associated Adverse Event will not be considered as a SAE, except when otherwise required by Regulatory Authorities. If a hospitalization is planned prior to the subjects receiving the first dose of study drug (at week 0), it will not be classified as either an AE or SAE. This also applies to situation of scheduled elective surgery where no AE is present or overnight hospitalizations occurring exclusively for logistic reasons (e.g., screening investigations which are impossible to complete within one day; no transportation available for the patient to return home on the same day). Non-complicated, preplanned elective surgery will not be considered an AE or SAE even if it involves hospitalization. However, if a hospitalization is unplanned or is a result of an adverse event, this will be considered an SAE, with the exception of hospitalization for steroid relapse treatment of a relapse.

All AEs Grade 4 according to Common Terminology Criteria for Adverse Events (CTCAE) v3.0 are considered 'serious', including deviation from reference ranges in laboratory tests.

Any event reported by the Investigator to the local authorities will follow the same reporting procedures as a Serious Adverse Event.

12.2.2 Procedures for Reporting Serious Adverse Events (SAEs)

If a SAE is reported, UCB or its representative must be informed within 24 hours of receipt of this information by the site (see contact numbers for SAEs reporting listed in section 0). The Investigator must forward to UCB (or its representatives) a duly completed “Investigator SAE report form” provided by UCB, even if the data are incomplete or if it is obvious that more data will be needed in order to draw any conclusions.

A copy of the Investigator SAE report form and the completion guide will be provided to the Investigator. The Investigator SAE report form has to be completed in English.

Additional information (e.g. autopsy or lab reports…) received by the investigator must be provided within 24 hours. All documents in local language must be accompanied by a translation in English, or the relevant information included in the same document must be summarized in the Investigator SAE report form.

UCB (or its representatives) will communicate safety information (Suspected Unexpected Serious Adverse Reactions – SUSAR) to the appropriate Regulatory Authorities and all active Investigators, in accordance with applicable regulatory requirements. The appropriate IEC will also be informed by the Investigator or the sponsor, as specified by the applicable regulatory requirements in each concerned country. Where applicable, investigators are to provide the Sponsor or its representatives with evidence of such IEC notification.

The Investigator is specifically requested to collect and report to UCB or its representative any Serious Adverse Events (even if the Investigator is certain that they are in no way associated with the investigational product), up to 30 days from the end of the study for each subject and to inform the participating subjects of the need to inform the investigator of any SAE within this period. Adverse Events that the Investigator thinks may be associated with the investigational product must be reported to UCB regardless of the time between the event and the end of the study.

The reference document for the assessment of the expectedness of SAEs for CDP323 is the Investigator's Brochure (7) . According to the European clinical trial directive, the expectedness of SAEs after application of gadolinium shall be determined by its Summary of Product Characteristics (SPC). AE which are typically caused by the underlying disease (MS), e.g., relapses, neurological deterioration, paresthesia, or asthenia, are considered 'expected' AEs.

12.2.3 Follow-up of Serious Adverse Events

A SAE should be followed-up until it has resolved/has a stable of sequelae or the Investigator no longer feels it is clinically significant.

Information on SAEs obtained after clinical database lock will be captured through the Global Drug Safety database without limitation of time.

13. STATISTICS

13.1 Statistical and Analytical Plans

13.1.1 Study Population(s)

The intention-to-treat population (ITT) will consist of all randomized subjects who took at least one dose of study medication. The per-protocol population (PP) is a subset of the ITT population, consisting of those subjects who had no major protocol deviations affecting the

pharmacodynamic or pharmacokinetic variables, as confirmed during a pre-analysis meeting prior to database lock. The PP population with partial or total exclusion of subjects may be different for PK and PD assessments depending on the type and timing of the deviations.

All PK and PD analyses will be performed on the PP population.

The safety population will consist of all subjects who have received one dose of study medication. All safety analyses will be performed on the safety population according to actually received treatment.

13.1.2 Safety and Other Variables

13.1.2.1 Pharmacokinetic Variables

Standard non-compartmental methods to determine t_{max}, C_{12h}, C_{max}, $t_{½}$, AUC(0–t), AUC, AUC(0-12), AUC_{SS}, C_{maxSS}, C_{12hSS}, V_z/F, λ_z, CL/F, and accumulation indices and accumulation indices for C_{12h}=R_{C12h}, C_{max}=R_{cmax} and AUC(0-12)= R_{AUC} for CT7758, CT533652-00, and CDP323 (if applicable).

The parameters will be calculated from the individual plasma drug concentration versus time profiles:

C_{max} , C_{maxSS}, and t_{max}	Value and time of the maximum plasma concentration will be directly obtained from the observed concentration vs. time curves.
C_{12h}, C_{12hSS}	Measured concentration at the end of a dosing interval (12h) at day 1 and at steady state (taken directly before next administration), will be directly obtained from the observed concentration vs. time curves.
AUC (0-x)/AUC(0-12)	Area under the plasma concentration vs. time curve observed from time 0 h up to the X (12) h data point, will be computed using the linear trapezoidal rule[)]. $AUC_{(t_i - t_{i+1})} = \frac{1}{2}\cdot(C_i + C_{i+1})\cdot(t_{i+1} - t_i)$
AUCτ,ss	Area under the concentration-time curve during a dosing interval at steady state
AUC(0-t)	Area under the plasma concentration vs. time curve observed from time 0 h up to the last measurable data point t, will be computed using the linear trapezoidal rule.

AUC:	Area under the curve extrapolated to infinity, calculated as: $\text{AUC}=AUC_{(0\text{ - t}_{Z})}+\dfrac{C_{z}}{\lambda_{z}}$ where λ_Z is the apparent terminal elimination rate constant and is the estimated slope of the linear regression of ln concentration vs. time, and C_Z is the last observed quantifiable concentration. To calculate AUC of the first dose, λ_Z of the last dose (steady state) will be used.
λ_z	Apparent terminal elimination rate constant.
$t_{½}$	Apparent terminal elimination half-life calculated as ln $2/\lambda_z$.
CL/F	Apparent total body clearance : CL/F = Dose/AUC and CL/F=Dose/AUC(0-τ) at steady-state
V_z/F	Apparent volume of distribution : $V_z/F = CL/F/\lambda_z$
R_{cmax}	The accumulation index for C_{max} will be calculated according to the following equation: $R_{C\max}=\dfrac{C_{\max,ss}}{C_{\max,dose1}}$
R_{C12h}	The accumulation index for C_{12h} will be calculated according to the following equation: $R_{C12h}=\dfrac{C_{12h,ss}}{C_{12h,dose1}}$
R_{AUC}	The accumulation index for AUC(0-12) will be calculated according to the following equation: $R_{AUC}=\dfrac{AUC\tau_{ss}}{AUC\tau_{dose1}}$

13.1.2.2 Pharmacodynamic (Biomarkers) Variables

Leukocytes and leukocyte subsets:

- Total and differential white blood cell count
- T-cells (CD3, CD4, CD8), B-cells (CD19, CD20, CD27 subsets), monocytes (CD14), hematopoietic progenitor cells (CD34), NK cells (CD56), NKT cells(CD3, CD56) and T_{reg} cells (CD4+CD25+FoxP3+), together with α4 expression for each subset.
- VCAM-1 inhibition

13.1.2.3 Safety Variables

Standard and disease-related safety variables:

- Adverse events (AEs) including MS relapses
- Vital sign parameters (heart rate, blood pressure, respiratory rate, body temperature, body weight)
- Blood chemistry: sodium, potassium, chloride, calcium, magnesium, urea, creatinine, SGOT, SGPT, GGT, AP, uric acid, bilirubin (total, conjugated and unconjugated), total serum protein, albumin, total cholesterol and fasting glucose
- hematology: erythrocytes, leukocytes, differential leukocyte count, thrombocytes, hemoglobin, hematocrit, mean corpuscular volume (MCV), mean corpuscular hemoglobin (MCH), mean corpuscular hemoglobin concentration (MCHC)
- Thyroid function tests: TSH
- Clotting tests: partial thromboplastin time and prothrombin time
- Urinalysis: pH, protein, glucose, erythrocytes, leukocytes
- 12-lead ECG: centrally measured or calculated variables: heart rate, PQ time, QRS time, and QT time in lead II; QTcF (corrected QT time calculated according to Fridericia's formula); central overall assessment of normal/abnormal findings with/without clinical relevance.
- cardiac telemetry for the 36 hours following the first intake of study drug.

Class-related

- Pre-post status for markers for infectious diseases:
    - Tuberculosis
    - Toxoplasmosis (IgM and IgG antibody to Toxoplasma gondii)
- Presence of JC virus DNA in serum and/or PBMCs
- Presence of potential signs of PML in MRI
- Presence of potential clinical signs and/or symptoms of PML

13.1.3 Statistical Evaluation

Statistical evaluation will be performed or supervised by the Global Exploratory Statistics department of UCB SA. All computations will be performed using SAS® version 9.1 or later (SAS Institute, NC, USA), PROC StatXact version 5 or later (Cytel Software, Cambridge, USA).

Summary statistics will consist of frequency tables (number of subjects and percent of subjects with that category) for categorical variables. For continuous variables, descriptive statistics (number of available observations, mean, median, standard deviation, minimum and maximum) will be tabulated. For PK and PD variables, geometric mean and coefficient of

variation will be also provided. All statistical tests will be carried out two-tailed at the 5% level of significance unless otherwise stated.

Baseline characteristics (gender, age, race, weight, BMI, BSA, creatinine clearance) for the ITT population will be summarized descriptively. Disease characteristics (EDSS, MS history and diagnosis, MS medication history) will be also summarized.

13.1.4 Pharmacokinetic and Pharmacodynamic (Biomarkers) Variable Analysis

Listings and summaries will be given for CDP323 and its 2 metabolites (CT7758 and CT533652-00). All PK statistical analyses using standard non-compartmental methods will be performed on the 3 analytes, unless otherwise specified. The PK analysis will be performed or supervised by UCB using a validated software (SAS® or WinNonlin version 5.0.1 or later (Pharsight Corporation, USA)).

Individual plasma concentrations, PK parameters and actual sampling times for PK and PD will be listed. Descriptive statistics will be calculated for plasma concentrations and derived PK and PD parameters. Concentrations will be summarized by treatment (or dose), day, and scheduled sampling time. PK and PD parameters will be presented by treatment and day (visit). Individual and mean plasma concentration-time profiles will be presented.

Exploratory analysis (mixed effects models using contrasts to compare groups) of the PD variables (leukocytes, α4 expression, VCAM-1 binding) will be performed to compare the effects of the different treatments (using derived variables like change from pre-dose, maximum value or change, AUC under time-effect profiles). Analysis of time-profiles of each PD variable and dose-response relationships will be evaluated. Based on data results, placebo-time effect if any, and dose effect will be modeled using different types of models (linear,asymptotic, Emax,..) to define maximal effect and dose (ED50) needed to reach 50% of the maximal effect. .

The following leukocytes and leukocyte subsets will be explored:

- Total and differential white blood cell count;
- T-cells (CD3, CD4, CD8), B-cells (CD19, CD20, CD27 subsets), monocytes (CD14), hematopoietic progenitor cells (CD34), NK cells (CD56), NKT cells (CD3, CD56) and T_{reg} cells (CD4+CD25+FoxP3+), together with α4 expression for each subset.
- VCAM- inhibition

Exploratory PK/PD modeling will also be performed using non linear mixed effects model (NONMEM software).

13.1.5 Safety Variable Analyses

Descriptive statistics will be presented for all safety variables by treatment and visit/period where appropriate. No statistical comparison between treatments will be performed, however.
Tolerability will be assessed by the incidence of AEs. Treatment-emergent AEs will be summarized descriptively by system organ class and preferred term. Additional tables will summarize AEs by severity and relationship to study drug as well as separate tables for AEs leading to withdrawal from the study and serious AEs.

Vital signs, ECG (12-lead and telemetry), laboratory test values, coagulation and thyroid function and physical examination findings, as well as change from baseline values, will be presented descriptively by treatment. Additionally, a categorical analysis will also be performed. The JVC viral status, tuberculosis and toxoplasmosis status, presence of potential clinical signs of PML will be tabulated. Neurological examinations and EDSS scores (by visit and change from screening) will be also provided in tables.

13.2 Determination of the Sample Size

No formal statistical testing will be performed and hence no formal sample size calculation was carried out for this exploratory study.

13.3 Changes in the Conduct of the Study or the Planned Analyses

A Statistical Analysis Plan (SAP) will be developed and approved prior to the study being unblinded. The full details of the statistical analyses will be provided in the SAP. Any deviations from the final SAP as well as changes from the protocol will be detailed in the SAP and discussed in the Study Report.

13.4 Statistical and Analytical Issues

13.4.1 Adjustments for Covariates and Interactions

Demographic and disease covariates if relevant will be used in the exploratory analysis.

13.4.2 Handling of Dropouts or Missing Data

There will be no special procedures for handling withdrawals and missing data.

13.4.3 Interim Analysis

No formal interim analysis is planned for this study.

13.4.4 Examination of Subgroups

Not applicable.

13.4.5 Use of Rescue Medication

Not applicable.

13.5 Pre-Analysis Review of Data

Before requesting the randomization code of the administered treatments and after all the data have been verified/coded/entered into a database, a blind review will be performed after last subject last visit. The purpose of this blind review will be to define the per protocol population, check the quality of the data, identify outliers, and to verify that the assumptions in the primary analyses are not violated. The review will also help decide how to deal with problems in the subject's data (for example: missing values, withdrawals, drop outs, protocol deviations). The pre-analysis reviewers should ensure that the results of this review are communicated to the study team before unblinding and that the actions taken introduce no bias in the treatment comparison.

After the pre-analysis review, resolution of all issues and documentation of all decisions, the database will be locked. Only after database lock the results of PK/PD will be incorporated in the database.

Safety data (SAEs: PML, serious infections (opportunistic infections, infections requiring parenteral or intra-muscular antibiotherapy and Tuberculosis cases), malignancies) that will be collected during the 12 month follow-up period, after database lock, will be used to amend the safety section of the clinical study report.

13.6 Criteria for Unblinding the Results

The randomization codes will be requested, released to data management and incorporated into the database after final locking of the database and approval of the final SAP.

13.7 Dictionaries

AEs, medical history, and medical procedures will be coded according to the latest available version of the MedDRA dictionary. Medications will be coded according to the last available version of WHO Drug dictionary.

14. ETHICS AND REGULATORY REQUIREMENTS

14.1 Approval

The final protocol and any amendments must be signed by the Principal Investigator of the center and by appropriate representatives of UCB.

The final protocol must be submitted to and approved by:

- a duly constituted Independent Ethics Committee (IEC)
- the relevant Regulatory Authorities, according to local regulations.

If any alterations to the protocol are required by these bodies, they can be implemented only with the written agreement of the Investigator and appropriate representative of UCB before further submission to the requesting body.

A copy of the IEC's written approval with clear identification of the submitted document(s) should be forwarded to the CPM.

A list of members attending the meeting (listed by function and affiliation) should also be forwarded to the CPM.

Before submission to an IEC, the consent form and any other written information to be provided to subjects (e.g. advertisement) must be submitted for internal UCB approval. The study is not allowed to start until the protocol and related documents (informed consent, advertisement, etc.) have received written approval from the IEC and Regulatory Authorities, if applicable, as well as until other GCP prerequisites are fulfilled.

If new information becomes available, it should be communicated without delay to the subject, the Investigator, the IEC, and regulatory authorities, wherever required.

The IEC must be notified promptly by the Investigator (or Sponsor, if applicable) in writing of the following:

- Deviations from, or changes to the protocol to eliminate immediate hazards to all the study subjects.
- Significant changes to the conduct of the study. Significant changes to the protocol can only be effected by a formal protocol amendment that must be submitted to the IEC and approved prior to implementation at the site.
- New information that may adversely affect subject safety or the conduct of the study.

The Investigator (or Sponsor, if applicable) should comply with the applicable regulatory requirements related to the reporting of safety information to the IEC and Regulatory Authorities.

14.2 Subject Information and Consent

Adequate information will be provided to the subject in both oral and written form and consent will be obtained in writing prior to performance of any study specific procedure. The content and process of obtaining informed consent will be in accordance with all applicable regulatory and IEC requirements.

UCB may provide a sample informed consent form and a subject information sheet. The final consent form must be approved by the IEC/IRB and should contain the applicable ICH-GCP elements in a language readily understood by the subject (i.e., lay terminology).

If the informed consent form is amended during the study, the Investigator (or the Sponsor, if applicable) must follow all applicable regulatory requirements pertaining to the approval of the amended informed consent form by the IEC and use of the amended form.

The Investigator, or a person designated by the Investigator, should fully inform the subject about all pertinent aspects of the study including the fact that the protocol has been granted the approval of the IEC and local regulatory authorities if required.

Subjects will be informed of the purpose of the study in unambiguous language they easily understand. Their participation is voluntary and they can at any time decide to stop their participation without any influence on their future care or treatment. The subjects must be informed about the main procedures used to guarantee their anonymity, especially during the analysis of their personal data. Subjects should be able to ask any questions about the study and to receive relevant answers.
They will receive complete written information in the Subject Information Sheet

After having received extensive information about the purpose and risks of the study and having had enough time to consider participation in the study, the subject must give their written consent by signing and dating the Informed Consent Form. This form will also be signed and dated by the person who obtained the informed consent and then retained by the Investigator. Obtaining of consent will be confirmed in the subject's medical records. The subject will receive a copy of the signed and dated consent form and the original will be filed in the Investigator's Study File.

The subject may withdraw their consent to participate in the study at any time. A subject is considered as enrolled in the study when he/she has signed the informed consent form. A Case Report Form must not be started, nor may any study specific procedure be performed for a given subject, without having obtained his/her written consent to participate in the study.

If any new information that could influence the subject's decision to stay in the study becomes available, this information will be transmitted to the subject without delay. In addition, the informed consent form must be amended accordingly or a separate consent form be created and submitted to the IEC/IRB for approval prior to being implemented for re-consent of all ongoing subjects in the study and for use in consenting all subjects entering the study from that point forward.

14.3 Subject Confidentiality

Subject confidentiality will be maintained at all times. Personnel from UCB (or its representative), from Regulatory Authorities and members of the IEC may inspect medical records and Case Report Forms for verification of the accuracy of data. These groups are obliged to respect medical secrecy and to refrain from divulging the subject's identity or any other personal information. Sites will be required to obliterate any possibly identifying information (e.g., name, social security number, address, etc.) on any materials, forms, or report prior to sending them to the sponsor or its designee.

Medical records will be handled by professional standards and existing local laws.

14.4 Informing the General Practitioner

If the subject agrees, the Investigator may inform the subject's regular physician of his/her participation in the study, by sending him/her the letter to the GP prepared by UCB and the Investigator.

15. STUDY MANAGEMENT AND ADMINISTRATION

15.1 Monitoring

Monitoring of the study is the responsibility of UCB and may be delegated to a CRO or a contract monitor. The Monitor (the individual responsible for monitoring) will advise the Investigator regarding the practical conduct of the study and maintaining compliance with the protocol, GCP, and all applicable regulatory requirements.

The Investigator will allow UCB or its representatives to review periodically, at mutually convenient times during the study and after the study has been completed, all CRFs and corresponding source documents (e.g., portions of office, hospital and laboratory records for each study participant). Therefore, the monitor will have direct access to these records. The monitoring visits provide UCB or its representatives with the opportunity to evaluate the progress of the study, to verify the accuracy and completeness of CRFs, ensure that all protocol requirements, applicable authorities regulations and Investigator's obligations are being fulfilled, and resolve any inconsistencies in the study records.

15.2 Direct Access to Source Data/Documents

The Investigator(s)/Institution(s) will permit study-related monitoring, audits by or on behalf of UCB, IEC review, and regulatory inspection(s), providing direct access to source data/documents.

Source documents (SD) are original records in which raw data are first recorded. These may be: hospital/clinic/GP records, charts, diaries, x-rays, laboratory results, printouts, pharmacy records, care records, ECG or other printouts, completed scales, Quality of Life Questionnaires, etc. Source documents should be kept in a secure, limited access area.
If they are not included in the clinical dossier/hospital file of the subjects, some data may be written directly in the CRF and will therefore be considered as source data. The list of those data will be reported in the CRF completion guide

All source documents must be, accurate, clear, unambiguous, permanent and capable of being audited. They should be made using some permanent form of recording (ink, typing, printing, optical disc). They should not be obscured by correcting fluid or have temporary attachments (such as removable self-stick notes). Photocopies of case report forms are not considered acceptable source documents. Photocopies will only be considered as acceptable source documents when used to establish permanent documentation of data captured on non-permanent media.

Source documents that are computer generated and stored electronically must be printed for review by the monitor (e.g. ECG reports). Once printed, these copies should be signed and dated by the Investigator and become a permanent part of the subject's source documents. The Investigator will authorize the monitor to compare the content of the printout and the data stored in the computer to ensure all data are consistent.

For subjects randomized to investigational product(s), the minimum requirements for source documents used in clinical studies are that they should contain the identity of the subject and study related identifiers (such as randomization/treatment number, CRF number, or similar), they should mention the subject's participation in the study and identification of that study (study title or number), they should record the obtaining of consent (date of consent), the exposure to investigational product, the subject's medical history, the concomitant medication treatments and dates (including contraceptive treatment), AEs and SAEs and the dates of the visits. The source documents should provide evidence that inclusion/exclusion criteria have been met.

Information recorded in the CRF must be consistent with entries in the source documents. The monitor will perform source documents verification (SDV) according to the SDV plan prepared for the study.

15.3 Audit and Inspection

The Investigator will permit study-related audits by auditors mandated by UCB and inspections by domestic or foreign regulatory authorities, after reasonable notice. The main purposes of an audit or inspection are to confirm that the rights and well-being of the subjects enrolled have been protected, that enrolled subjects (i.e. signing consent and undergoing study procedures) are appropriate for the study, and that all data relevant for the evaluation of the investigational product have been processed and reported in compliance with the planned arrangements, the protocol, facility and IEC SOPS, ICH/GCP and applicable regulatory requirements. The Investigator will provide direct access to all study documents, source records and source data. If an inspection by a regulatory authority is announced, the Investigator will immediately inform UCB.

15.4 Case Report Forms (CRF) and Screen Failures

Data reflecting participant experience with the drug(s) under investigation will be reported to UCB. These data will be recorded on the Case Report Forms (CRF). The CRF is essentially a data entry form and may not routinely constitute the original (or source) medical record. CRF should be kept in a secure, limited access area.

CRF will be signed and dated by an Investigator or the Principal Investigator as indicated. The Investigator's or Principal Investigator's signature on the CRF attests to its accuracy and completeness. Paper CRF will be completed in dark ballpoint pen, and must be legible. If an entry in a CRF needs to be changed, the correction will be made as follows:

- Cross-out the initial entry (must still be legible).
- Write the correct entry next to it together with your initials, the date and justification if necessary. Correcting fluid, erasure or any form of obliteration of data in CRFs is not permitted except to obliterate information that could specifically identify a subject (i.e., a name written on a subject diary).

UCB cannot interpret a blank answer as NONE or NA (not applicable); therefore, all fields must be completed. Please mark data which could not be recorded as follow: ND for "Not Done", NA for "Not Applicable", UN for "Unknown".

Data reported in the CRF, which are derived from source documents, should be consistent with the source documents or the discrepancies should be explained in those source documents.

The Investigator will keep a subject screening log to document identification of subjects who entered pre-study screening.

All supportive documentation submitted to UCB in addition to the Case Report Form, such as laboratory results or hospitalization records, must be clearly identified with the study number,

study participant number, and study participant initials; any personal information, including the study participant's name, must be removed or rendered illegible to preserve individual confidentiality. All original lab reports will remain at the study site. Copies of report will be placed in the CRF binder (where directed) and forwarded to UCB. This sentence may be expanded based on study specific parameters regarding the retrieval of other original source documents including that which also may function as case report form.

In case of screen failure, the following data must be collected in the CRF/screen failure booklet, entered in the clinical database and cleaned: visit date, demography, adverse events (if any exist) and reason for screen failure (i.e. randomization/ treatment allocation status).

These data should therefore be monitored (source data verified) and retrieved from the site. The other recorded data (if any) should be retrieved but will not be databased and/or cleaned.

15.5 Adherence to Protocol

The Investigator/institution should conduct the study in compliance with the protocol agreed to by the sponsor and, if applicable, by the appropriate regulatory authority(ies) and which has been approved by the IEC. The Investigator/institution and the sponsor should sign the protocol to confirm agreement.

The Investigator should not deviate from the protocol. In medical emergencies, the Investigator will use his/her medical judgment and will remove the study participant from immediate hazard before notifying UCB or its representatives and the IEC/IRB in writing regarding the type of emergency and the course of action taken. Significant changes to the protocol will ONLY be made as an amendment to the protocol and must be approved by UCB, the IEC and the appropriate regulatory authorities, if applicable prior to being implemented.
In exceptional circumstances, subject-specific deviations from the protocol may occur. All deviations should be recorded on an ongoing basis to allow regular assessment for the need of an amendment. Protocol Deviations could invalidate the insurance coverage.

Any protocol deviation will be documented and explained by the Investigator or the person designated by the Investigator and will be included in the final Clinical Study Report.

15.6 Investigator Site File

All documents required for the conduct of the study as specified in the ICH GCP guidelines will be maintained by the Investigator in an orderly manner and made available for monitoring, auditing by UCB or its designee and/or inspection.

15.7 Data Handling

CRF data will be entered in a validated electronic database using a clinical data management system (CDMS). Computerized data cleaning checks will be used in addition to manual review to check for discrepancies and to ensure consistency of the data. An electronic audit trail system will be used to track all data changes in the database subsequent to the reconciliation of the double-entered data. The SAS system will be used for the statistical analysis of the data. Regular back-ups of the electronic data will be performed.

15.8 Termination of Study

Upon completion of the study, the monitor will conduct the following activities in conjunction with the Investigator, as appropriate:

- return of all study data to UCB or its representatives while maintaining original source documents
- data clarification and/or resolution
- accountability, reconciliation and arrangements for used and unused investigational products
- review of site study records for completeness
- return of treatment codes to UCB or its representatives
- discussion/reminder on archiving responsibilities
- discussion of IEC requirements for study termination
- arrangements for unused CRFs, lab supplies and any other study related supplies.

In addition, UCB reserves the right to temporarily suspend or prematurely discontinue this study either at a single site, multiple sites, or at all sites at any time for reasons including, but not limited to, safety or ethical issues, inaccurate or incomplete data recording, non-compliance, or unsatisfactory enrollment with respect to quality or quantity.

If the study is prematurely terminated or suspended, UCB or its representatives will inform the Investigators/institutions, and the regulatory authority(ies) of the termination or suspension and the reason(s) for the termination or suspension, in accordance with applicable regulatory requirement(s). The IEC should also be informed and provided with reason(s) for the termination or suspension by the Sponsor or by the Investigator/institution, as specified by the applicable regulatory requirement(s). In addition, arrangements will be made for the return of all unused investigational products and other material in accordance with UCB procedures for the study.

15.9 Clinical Study Report

UCB will prepare a clinical study report, in accordance with the relevant ICH guidelines. The report will include a thorough description of the clinical and laboratory methods, a

discussion of the results and a list of all measurements as specified in the Statistical Analysis Plan. This report may be included in submissions to government drug regulatory authorities worldwide, or used for whatever reason considered appropriate by UCB. No information contained in the report may be used without written approval of UCB.

The Investigator will have an opportunity to comment on the draft version. He/she must give his/her comments within 7 days of receiving the report. In addition, he/she will sign the report for approval within 7 days of receipt of the revised version or a satisfactory reply to these comments.

15.10 Insurance and Liability

UCB has taken out an insurance policy, for the total duration of the study, covering the subjects,, in respect of the risks involved in this study according to this protocol. In the case of injury or disability deriving from participation in the study, the subject is requested to inform the treating physician responsible for the study without delay.

15.11 Archiving and Data Retention

The Investigator will maintain adequate records for the study including CRFs, medical records, laboratory results, informed consent documents, drug dispensing and disposition records, safety reports, information regarding participants who discontinued, and other pertinent data.

All essential documents are to be retained by the Investigator until at least two years after the last approval of a marketing application in an ICH region and until there are no pending or contemplated marketing applications in an ICH region or at least two years have elapsed since the formal discontinuation of clinical development of the investigational product. These documents should be retained for a longer period however if required by the applicable regulatory requirement(s) or by an agreement with UCB (ICH-GCP Guideline-section 4.9.5). The Investigator will contact UCB for authorization prior to the destruction of any study records or in the event of accidental loss or destruction of any study records. The Investigator will also notify UCB should he/she relocate or move the study related files to a location other than that specified in the Sponsor's study master file.

15.12 Allocation of Responsibilities

The Investigator is responsible for the implementation of the protocol but can delegate tasks to the research team. The Investigator remains responsible for coordinating and informing his/her staff about the protocol and any possible changes made to it. The Investigator should maintain a site signature and delegation of responsibility log. The list should be kept up to date.

15.13 Curriculum Vitae (CV)

A signed and dated FDA Form 1572 and recent (updated every 2 years) CV (in English) are required from each Investigator showing a current affiliation with the research center. All sub-Investigators listed on the FDA Form 1572 should also date and sign a recent English version of their CVs. Any changes to the site personnel should be updated on a new FDA Form 1572.

15.14 Financial Disclosure

A Financial Disclosure Statement must be obtained for everyone listed on the FDA form 1572. This must be collected before subject enrollment, at the site close-out visit and one year after the study is completed (i.e. one year after Last Patient Last Visit 10 for the study).

15.15 Good Clinical Practice

Non-compliance with the protocol, ICH/GCP or local regulatory requirements by the Investigator, institution, institution staff or designees of the Sponsor will lead to prompt action by UCB to secure compliance. Continued non-compliance may result in the termination of the participants' involvement in the study.

15.16 Disclosure of Clinical Trial Information

UCB alone shall be responsible for the registration of clinical trials in a public trials registry and for the disclosure of trials results on a publicly accessible website [(12)] .

15.17 Publication

Authorship of planned manuscripts for submission to medical journals shall be determined in accordance with the International Committee of Medical Journal Editors (ICMJE) Uniform Requirements for Manuscripts Submitted to Biomedical Journals.

All sites and their investigators agree that if a site is part of a multi-center study, site and investigator(s) shall coordinate in advance any intended disclosure of the results of the trial with UCB to ensure that the results of individual sites are not published or presented before those of the multi-centre study, unless otherwise agreed by in writing by UCB.

Subject to the following paragraph, the authors have the final responsibility for the content of their own publication(s) and the decision to submit it/them for publication.

Any planned manuscript, presentation, abstract or other intended disclosure of the results of the trial or otherwise originating from the study shall be made available for review to UCB at

least thirty (30) days before submission for publication or any other means of disclosure in order to allow UCB to protect its intellectual property.

In the rare event that such disclosure would affect the patentability of any invention to which UCB has rights, UCB shall have the right to request an additional delay to the proposed disclosure of no more than ninety (90) days so as to allow UCB to preserve its intellectual property.

16. REFERENCES

1. McDonald WI, Compston A, Edan G et al. Recommended diagnostic criteria for multiple sclerosis: guidelines from the International Panel on the diagnosis of multiple sclerosis. Ann Neurol 2001; 50:121-27.

2. Polman CH, Reingold SC, Edan G et al. Diagnostic Criteria for Multiple Sclerosis: 2005 Revisions to the "McDonald Criteria". Ann Neurol 2005; 58:840-6.

3. Lublin FD, Reingold SC. Defining the clinical course of multiple sclerosis: results of an international survey. Neurology 1996; 46:907-11.

4. EMEA/CHMP/EWP/561/98 revision 1. Guideline on clinical investigation of medicinal products for the treatment of multiple sclerosis. (16 November 2006).

5. Yousry TA Major EO, Ryschkewitsch C et al. Evaluation of patients treated with natalizumab for progressive multifocal leukoencephalopathy. N Engl J Med 2006; 354:924-33.

6. Stüve O, Marra CM, Jerome KR et al. Immune surveillance in multiple sclerosis patients treated with natalizumab. Ann. Neurol 2006; 59:743-7.

7. Investigators' Brochure CDP323 -Version 7 – UCB Reference Code RXCE07L1603

8. Wolf C. Double-blind, placebo-controlled, randomized, parallel-group Phase II study in subjects with relapsing forms of multiple sclerosis (MS) to evaluate the safety, tolerability, and effects of two doses of CDP323 over 24 weeks with a rater-blind MRI follow-up over 12 weeks; 2007. UCB Study Number C32322 (8) . UCB Protocol Reference Code RPCE06D2833.

9. Miller DH, Khan OA, Sheremata WA et al. A Controlled Trial of Natalizumab for Relapsing Multiple Sclerosis. N Engl J Med 2003; 348:15-23.

10. Levey AS, Bosch JP, Lewis JB et al. A more accurate method to estimate glomerular filtration rate from serum creatinine: A new prediction equation. Ann Intern Med 1999.130.461-70.

11. Public Assessment Report (26 June 2007) of The European Pharmacovigilance Working Party: "Increased risk of nephrogenic fibrosing dermopathy/nephrogenic systemic fibrosis and gadolinium-containing MRI contrast agents"

12. Joint Position on the Disclosure of Clinical Trial Information via Clinical Trial Registries and Databases, January 2005.

13. Fridericia LS. Die Systolendauer im Elektrokardiogramm bei normalen Menschen und bei Herzkranken. Acta Med Scand 1920; 53:469-86.

17. APPENDICES

17.1 Expanded Disability Status Scale (EDSS)

NEUROLOGICAL EXAMINATION (EDSS)

CRF number: |_|_|_|_|_|_|_|

Subject's initials: |_|_|_|_|

Subject's number: |_|_|_|_| / |_|_|_|_|_|

Site N° / Identification N°

GENERAL GUIDELINES

Functional Systems (FS)

"Signs only" is noted when the examination reveals signs of which the patient is unaware.
A score of 1 in the Functional Systems implies that the patient is not aware of the deficit and that the deficit or sign does not interfere with normal daily activities (with the exceptions of optic, vegetative and cerebral functions).
Symptoms which are not MS-related will not be taken into consideration for assessments, but should be noted.

Visual (optic) functions

Visual acuity

The visual acuity score is based upon the line on the Snellen chart at 20 feet (6.1 m) or 5 m for which the patient makes no more than one error (use best available correction).

Visual fields

signs only	deficits present only on formal testing
moderate	patient aware of deficit, but incomplete hemianopsia on examination
marked	complete homonymous hemianopsia or equivalent

Scotoma

small	detectable only on formal (confrontational) testing
large	spontaneously reported by patient

NEUROLOGICAL EXAMINATION (EDSS)

**Visit:** *(Tick one box)*	**Subject's number**	**Subject's initials**
☐ Visit 1 ☐ Visit 8 ☐ Visit 10<br>☐ Visit 11 ☐ UNS	⌊_⌊_⌊_⌋ / ⌊_⌊_⌊_⌊_⌋	⌊_⌊_⌊_⌋

**Neurological examination**		
Date of examination:	⌊_⌊_⌋ ⌊_⌊_⌊_⌋ ⌊_⌊_⌊_⌊_⌋<br>DD MON YYYY	
**Visual (optic) functions –** neurological examination		
	Right	Left
Visual acuity (corrected)	⌊____⌋%	⌊____⌋%
Visual fields	0☐ normal<br>1☐ signs only<br>2☐ moderate<br>3☐ marked	0☐ normal<br>1☐ signs only<br>2☐ moderate<br>3☐ marked
Scotoma	0☐ none<br>1☐ small<br>2☐ large	0☐ none<br>1☐ small<br>2☐ large
Disc pallor	0☐ not present<br>1☐ present	0☐ not present<br>1☐ present
**Kurtzke Functional System Score**		

Optic functions	0☐ normal<br>1☐ disc pallor and/or mild scotoma and/or visual acuity of worse eye (corrected) less than 30/30 (1.0) but better than 20/30 (0.67)<br>2☐ worse eye with large scotoma and/or maximal visual acuity (corrected) of 20/30 to 20/59 (0.67-0.34)<br>3☐ worse eye with large scotoma or moderate decrease in fields and/or maximal visual acuity (corrected) of 20/60 to 20/99 (0.33-0.2)<br>4☐ worse eye with marked decrease of fields and/or maximal visual acuity (corrected) of 20/100 to 20/200 (0.1-0.2); grade 3 plus maximal acuity of better eye of 20/60 (0.3) or less<br>5☐ worse eye with maximal visual acuity (corrected) less than 20/200 (0.1); grade 4 plus maximal acuity of better eye of 20/60 (0.3) or less<br>6☐ grade 5 plus maximal visual acuity of better eye of 20/60 (0.3) or less

Brainstem function / cranial examination

Assessment of impairment/disability

signs only	clinically detectable numbness, facial weakness, or cranial nerve deficits of which patient is not aware
mild	clinically detectable numbness, facial weakness, dysarthria or cranial nerve deficits of which patient is aware
moderate	diplopia with incomplete paralysis of any eye movement, impaired discrimination of sharp/dull in 1 or 2 trigeminal branches, trigeminal neuralgia (at least one attack in last 24 hours), weakness of eye closure, cannot hear finger rub and/or misses several whispered numbers, obvious dysarthria during ordinary conversation impairing comprehensibility
severe	complete loss of movement of either eye in one or more directions, impaired discrimination of sharp/dull or complete loss of sensation in the entire distribution of one or both trigeminal nerves, unilateral or bilateral facial palsy with lagophthalmus or difficulty with liquids, sustained difficulty with swallowing, incomprehensible voice

Nystagmus

mild	gaze evoked nystagmus below limits of ”moderate” (usual equivalent is grade 1 in FS)
moderate	sustained nystagmus on 30°_horizontal or vertical gaze, but not in primary position, patient may or may not realize disturbance (usual equivalent is grade 2 in FS)
severe	sustained nystagmus in primary position or coarse persistent nystagmus in any direction interfering with visual acuity, complete internuclear ophthalmoplegia with sustained nystagmus of abducting eye, oscillopsia

NEUROLOGICAL EXAMINATION (EDSS) *(Continued)*	
**Brainstem functions / cranial examination –** neurological examination	
EOM (extra ocular movements) impaired	0☐ no<br>1☐ signs only<br>2☐ mild<br>3☐ moderate<br>4☐ severe
Nystagmus	0☐ normal<br>1☐ mild<br>2☐ moderate<br>3☐ severe
Trigeminal damage	0☐ no<br>1☐ signs only<br>2☐ mild<br>3☐ moderate<br>4☐ severe
Facial weakness	0☐ no<br>1☐ signs only<br>2☐ mild<br>3☐ moderate<br>4☐ severe
Hearing loss	0☐ no<br>1☐ signs only<br>2☐ mild<br>3☐ moderate<br>4☐ severe
Dysarthria	0☐ no<br>1☐ signs only<br>2☐ mild<br>3☐ moderate<br>4☐ severe
Dysphagia	0☐ no<br>2☐ mild<br>3☐ moderate<br>4☐ severe
Other bulbar signs	0☐ no<br>2☐ mild<br>3☐ moderate<br>4☐ severe
If other, specify:	

**NEUROLOGICAL EXAMINATION (EDSS)** *(Continued)*	
**Kurtzke Functional System Score**	
Brainstem functions	0☐ no<br>1☐ signs only<br>2☐ moderate nystagmus and/or other mild disability<br>3☐ severe nystagmus and/or marked extraocular weakness and/or moderate disability of other cranial nerves<br>4☐ marked dysarthria and/or other marked disability<br>5☐ inability to swallow and/or speak

NEUROLOGICAL EXAMINATION (EDSS) *(Continued)*

Pyramidal functions (page 1 of 3) – neurological examination

Reflexes	Right	Left	Accentuation
Biceps	0☐ absent<br>1☐ weak<br>2☐ normal<br>3☐ exaggerated<br>4☐ cloniform<br>5☐ inexhaustible	0☐ absent<br>1☐ weak<br>2☐ normal<br>3☐ exaggerated<br>4☐ cloniform<br>5☐ inexhaustible	1☐ right<br>2☐ left
Triceps	0☐ absent<br>1☐ weak<br>2☐ normal<br>3☐ exaggerated<br>4☐ cloniform<br>5☐ inexhaustible	0☐ absent<br>1☐ weak<br>2☐ normal<br>3☐ exaggerated<br>4☐ cloniform<br>5☐ inexhaustible	1☐ right<br>2☐ left
Radial	0☐ absent<br>1☐ weak<br>2☐ normal<br>3☐ exaggerated<br>4☐ cloniform<br>5☐ inexhaustible	0☐ absent<br>1☐ weak<br>2☐ normal<br>3☐ exaggerated<br>4☐ cloniform<br>5☐ inexhaustible	1☐ right<br>2☐ left
Knee (patellar)	0☐ absent<br>1☐ weak<br>2☐ normal<br>3☐ exaggerated<br>4☐ cloniform<br>5☐ inexhaustible	0☐ absent<br>1☐ weak<br>2☐ normal<br>3☐ exaggerated<br>4☐ cloniform<br>5☐ inexhaustible	1☐ right<br>2☐ left
Ankle	0☐ absent<br>1☐ weak<br>2☐ normal<br>3☐ exaggerated<br>4☐ cloniform<br>5☐ inexhaustible	0☐ absent<br>1☐ weak<br>2☐ normal<br>3☐ exaggerated<br>4☐ cloniform<br>5☐ inexhaustible	1☐ right<br>2☐ left
Plantar response	0☐ flexor<br>1☐ neutral<br>2☐ extensor	0☐ flexor<br>1☐ neutral<br>2☐ extensor	1☐ right<br>2☐ left
Abdominal cutaneous reflexes	0☐ normal<br>1☐ weak<br>2☐ absent	0☐ normal<br>1☐ weak<br>2☐ absent	1☐ right<br>2☐ left
Palmomental reflex (optional)	0☐ absent<br>1☐ present	0☐ absent<br>1☐ present	1☐ right<br>2☐ left

Pyramidal functions - page 2

Limb strength

The weakest muscle in each group defines the score for that group.
Use of functional tests like jumping with one foot, walking on toes or heels are recommended in order to assess grades 3-5 at the BMRC scale.

NEUROLOGICAL EXAMINATION (EDSS) *(Continued)*

Pyramidal functions (page 2 of 3) – neurological examination

Limb strength	Right	Left	BMRC Rating Scale
Shoulder	|____|	|____|	0☐ no activity<br>1☐ visible contraction without visible joint movement<br>2☐ visible movements with elimination of gravity<br>3☐ movements against gravity possible but impaired<br>4☐ movements against resistance possible but impaired<br>5☐ normal strength
Elbow flexors	|____|	|____|	
Elbow extensors	|____|	|____|	
Hand/finger flexors	|____|	|____|	
Hand/finger extensors	|____|	|____|	
Hip flexion	|____|	|____|	
Knee flexors	|____|	|____|	
Knee extensors	|____|	|____|	
Foot/toe flexors	|____|	|____|	
Foot/toe extensors	|____|	|____|	

**Functional tests** *(optional)*	Right	Left
Position test upper extremities, pronation	0☐ none<br>1☐ mild<br>2☐ evident	0☐ none<br>1☐ mild<br>2☐ evident
Position test upper extremities, sinking	0☐ none<br>1☐ mild<br>2☐ evident	0☐ none<br>1☐ mild<br>2☐ evident
Position test lower extremities, sinking (45°, stretched knees)	0☐ none<br>1☐ mild<br>2☐ evident<br>3☐ lifts leg only separately<br>|____| ° reached<br>4☐ even separate lifting not possible	0☐ none<br>1☐ mild<br>2☐ evident<br>3☐ lifts leg only separately<br>|____| ° reached<br>4☐ even separate lifting not possible
Walking on heels	0☐ normal<br>1☐ impaired<br>2☐ not possible	0☐ normal<br>1☐ impaired<br>2☐ not possible
Walking on tiptoes	0☐ normal<br>1☐ impaired<br>2☐ not possible	0☐ normal<br>1☐ impaired<br>2☐ not possible
Hopping on one foot	0☐ normal<br>1☐ 6-10 times<br>2☐ 1-5 times<br>3☐ not possible	0☐ normal<br>1☐ 6-10 times<br>2☐ 1-5 times<br>3☐ not possible

Pyramidal functions - page 3

Limb spasticity

mild	barely increased muscular tone after rapid flexion of an extremity
severe	barely surmountable increased spastic tonus after rapid flexion of an extremity

**NEUROLOGICAL EXAMINATION (EDSS)** *(Continued)*		
**Pyramidal functions (page 3 of 3) –** neurological examination		
Spasticity	Right	Left
Arm	0☐ none<br>1☐ mild<br>2☐ moderate<br>3☐ severe<br>4☐ contracted	0☐ none<br>1☐ mild<br>2☐ moderate<br>3☐ severe<br>4☐ contracted
Leg	0☐ none<br>1☐ mild<br>2☐ moderate<br>3☐ severe<br>4☐ contracted	0☐ none<br>1☐ mild<br>2☐ moderate<br>3☐ severe<br>4☐ contracted
Gait	0☐ none<br>1☐ barely perceptible<br>2☐ evident, minor interference with function<br>3☐ permanent shuffling, major interference with function	0☐ none<br>1☐ barely perceptible<br>2☐ evident, minor interference with function<br>3☐ permanent shuffling, major interference with function

**Kurtzke Functional System Score**	
Pyramidal functions	0☐ normal<br>1☐ abnormal signs without disability<br>2☐ minimal disability, patient complains about fatiguability in motor tasks and/or BMRC grade 4 in one or two muscle groups<br>3☐ -mild to moderate paraparesis or hemiparesis (usually BMRC grade 4 in more than two muscle groups or BMRC grade 3 in one or two), full range of movement against gravity<br>-severe monoparesis, refers to BMRC grade 2 or less in one muscle group<br>4☐ -marked paraparesis or hemiparesis (usually BMRC grade 2 in 2 limbs)<br>-moderate tetraparesis (refers to BMRC grade 3 in 3 or more limbs)<br>-monoplegia (BMRC grade 0 or 1 in one limb)<br>5☐ -paraplegia, BMRC grade 0 or 1 in all muscle groups of the lower limbs<br>-hemiplegia<br>-marked tetraparesis (BMRC grade 2 or less in 3 or more limbs)<br>6☐ -tetraplegia (BMRC grade 0 or 1 in all muscle groups of upper and lower limbs)

Cerebellar functions - page 1

Truncal ataxia

mild	swaying with eyes closed
moderate	swaying with eyes opened
severe	unable to sit without assistance
Limb ataxia	
mild	tremor or clumsy movements seen easily, minor interference with function
moderate	tremor or clumsy movements interfere with function in all spheres
severe	most functions are very difficult

UE	upper extremities
LE	lower extremities

NEUROLOGICAL EXAMINATION (EDSS) *(Continued)*		
**Cerebellar functions (page 1 of 2) –** neurological examination		
Head tremor	0☐ none<br>1☐ mild<br>2☐ moderate<br>3☐ severe	
Truncal ataxia	0☐ none<br>1☐ signs only<br>2☐ mild<br>3☐ moderate<br>4☐ severe	
	Right	Left
Tremor/dysmetria (UE)	0☐ none<br>1☐ signs only<br>2☐ mild<br>3☐ moderate<br>4☐ severe	0☐ none<br>1☐ signs only<br>2☐ mild<br>3☐ moderate<br>4☐ severe
Tremor/dysmetria (LE)	0☐ none<br>1☐ signs only<br>2☐ mild<br>3☐ moderate<br>4☐ severe	0☐ none<br>1☐ signs only<br>2☐ mild<br>3☐ moderate<br>4☐ severe
Rapid alternate movements impaired UE	0☐ none<br>1☐ signs only<br>2☐ mild<br>3☐ moderate<br>4☐ severe	0☐ none<br>1☐ signs only<br>2☐ mild<br>3☐ moderate<br>4☐ severe
Rapid alternate movements impaired LE	0☐ none<br>1☐ signs only<br>2☐ mild<br>3☐ moderate<br>4☐ severe	0☐ none<br>1☐ signs only<br>2☐ mild<br>3☐ moderate<br>4☐ severe

Other, e.g. rebound<br><br><br><br><br>*specify:*	0☐ none<br>2☐ mild<br>3☐ moderate<br>4☐ severe	0☐ none<br>2☐ mild<br>3☐ moderate<br>4☐ severe

Cerebellar functions - page 2

Gait ataxia

mild	abnormal balance only on heel or toe walking, or walking along a line
moderate	abnormal balance on ordinary walking or while seated
severe	unable to walk more than a few steps or requires support by another person or walking aid because of ataxia

Romberg test

mild	mild insecurity with eyes closed
moderate	not stable with eyes closed
severe	not stable with eyes opened

Note

The presence of severe gait ataxia alone results in a grade of 3 in the cerebellar FS. If weakness interferes with the testing of ataxia, score the patient's actual performance, but also indicate the possible role of weakness by marking the respective box

**NEUROLOGICAL EXAMINATION (EDSS)** *(Continued)*	
**Cerebellar functions (page 2 of 2) –** neurological examination	
Gait ataxia (eyes open)	0☐ normal<br>1☐ signs only<br>2☐ mild<br>3☐ moderate<br>4☐ severe
Straight line walking (eyes open)	0☐ without problems<br>1☐ impaired<br>2☐ not possible
Romberg test	0☐ none<br>1☐ mild<br>2☐ moderate<br>3☐ severe
Weakness (grade 3 or more on pyramidal) interferes with testing	1☐ no<br>2☐ yes
**Kurtzke Functional System Score**	
Cerebellar functions	0☐ normal<br>1☐ abnormal signs without disability<br>2☐ mild ataxia<br>3☐ moderate truncal and/or moderate limb ataxia<br>4☐ severe ataxia in all limbs and/or trunk<br>5☐ unable to perform coordinated movements due to ataxia

Sensory functions - page 1

Superficial sensation - Touch/pain

signs only	patient is not aware of deficit but slightly reduced sensation of feeling (e.g. temperature, figure writing)
mild	patient is aware of impaired light touch or pain, but able to discriminate sharp/dull
moderate	impaired discrimination of sharp/dull
severe	no discrimination of sharp/dull and/or unable to feel light touch

Vibration sense

mild graded tuning fork 5-7 of 8

alternatively: feels vibration more than 10 sec but less than examiner

moderate graded tuning fork 1-4 of 8

alternatively: feels vibration more than 2 sec but less than 11 sec

marked complete loss of vibration sense

Position sense

mild	1-2 incorrect responses on testing, only distal joints affected
moderate	misses many movements of fingers or toes, proximal joints affected
marked	no perception of movement

UE	upper extremities
LE	lower extremities

NEUROLOGICAL EXAMINATION (EDSS) *(Continued)*		
**Sensory functions (page 1 of 2) –** neurological examination		
	Right	Left
Superficial sensation (touch/pain) UE	0☐ none<br>1☐ signs only<br>2☐ mild<br>3☐ moderate<br>4☐ severe<br>5☐ complete loss, anaesthesia	0☐ none<br>1☐ signs only<br>2☐ mild<br>3☐ moderate<br>4☐ severe<br>5☐ complete loss, anaesthesia
Superficial sensation trunk	0☐ none<br>1☐ signs only<br>2☐ mild<br>3☐ moderate<br>4☐ severe<br>5☐ complete loss, anaesthesia	0☐ none<br>1☐ signs only<br>2☐ mild<br>3☐ moderate<br>4☐ severe<br>5☐ complete loss, anaesthesia
Superficial sensation LE	0☐ none<br>1☐ signs only<br>2☐ mild<br>3☐ moderate<br>4☐ severe<br>5☐ complete loss, anaesthesia	0☐ none<br>1☐ signs only<br>2☐ mild<br>3☐ moderate<br>4☐ severe<br>5☐ complete loss, anaesthesia
Vibration sense UE	0☐ normal<br>1☐ mild<br>2☐ moderate<br>3☐ marked	0☐ normal<br>1☐ mild<br>2☐ moderate<br>3☐ marked
Vibration sense LE	0☐ normal<br>1☐ mild<br>2☐ moderate<br>3☐ marked	0☐ normal<br>1☐ mild<br>2☐ moderate<br>3☐ marked
Position sense UE	0☐ normal<br>1☐ mild<br>2☐ moderate<br>3☐ marked	0☐ normal<br>1☐ mild<br>2☐ moderate<br>3☐ marked
Position sense LE	0☐ normal<br>1☐ mild<br>2☐ moderate<br>3☐ marked	0☐ normal<br>1☐ mild<br>2☐ moderate<br>3☐ marked

**NEUROLOGICAL EXAMINATION (EDSS)** *(Continued)*		
**Sensory functions (page 2 of 2) –** neurological examination		
Lhermitte	0☐ negative<br>1☐ positive	
	Right	Left
Paraesthesiae UE (optional)	0☐ none<br>1☐ present	0☐ none<br>1☐ present
Paraesthesiae trunk (optional)	0☐ none<br>1☐ present	0☐ none<br>1☐ present
Paraesthesiae LE (optional)	0☐ none<br>1☐ present	0☐ none<br>1☐ present
**Kurtzke Functional System Score**		
Sensory functions (paraesthesia or Lhermitte-sign alone do not affect FS score)	0☐ normal<br>1☐ mild vibration or figure–writing decrease only, in 1 or 2 limbs<br>2☐ -mild decrease in touch or pain or position sense, and/or moderate decrease in vibration in 1 or 2 limbs<br>-mild vibration or figure–writing decrease alone in 3 or 4 limbs<br>3☐ -moderate decrease in touch or pain or position sense, and/or essentially lost vibration in 1 or 2 limbs<br>-mild decrease in touch or pain and/or moderate decrease in all proprioceptive tests in 3 or 4 limbs<br>4☐ -marked decrease in touch or pain or loss of proprioception, alone or combined, in 1 or 2 limbs<br>-moderate decrease in touch or pain and/or severe proprioceptive decrease in more than 2 limbs<br>5☐ -loss (essentially) of sensation in 1 or 2 limbs<br>-moderate decrease in touch or pain and/or loss of proprioception for most of the body below the head<br>6☐ sensation essentially lost below the head	

Bladder and bowel functions

Hesitancy/retention

mild	no major impact on lifestyle
moderate	urine retention, frequent UTI
severe	requires catheterisation to void bladder
loss of function	overflow incontinence
Urgency/incontinence	
mild	no major impact on lifestyle
moderate	rare incontinence, no more than once a week, must wear pads
severe	frequent incontinence, several times a week up to once daily, must wear urinal
loss of function	loss of bladder control
Bowel	
mild	no incontinence, no major impact on lifestyle, constipation
moderate	must wear pads or alter lifestyle to be near lavatory
severe	in need of intermittent enemata

NEUROLOGICAL EXAMINATION (EDSS) *(Continued)*	
**Bladder and bowel functions –** neurological examination	
Hesitancy/retention	0☐ none<br>1☐ mild<br>2☐ moderate<br>3☐ severe<br>4☐ loss of function
Urgency/incontinence	0☐ none<br>1☐ mild<br>2☐ moderate<br>3☐ severe<br>4☐ loss of function
Catheterisation	0☐ none<br>1☐ intermittent self catheterisation<br>2☐ constant
Bowel dysfunction	0☐ none<br>1☐ mild<br>2☐ moderate<br>3☐ severe<br>4☐ complete loss of function
Sexual dysfunction (optional)	0☐ none<br>1☐ mild<br>2☐ moderate<br>3☐ severe<br>4☐ loss of function
**Kurtzke Functional System Score**	
Vegetative functions	0☐ normal<br>1☐ mild urinary hesitancy, urgency and/or constipation<br>2☐ moderate urinary hesitancy and/or urgency and/or rare incontinence and/or severe constipation<br>3☐ frequent urinary incontinence or intermittent self catheterisation, needs constantly enemata or manual measures to evacuate bowel<br>4☐ in need of almost constant catheterisation<br>5☐ loss of bladder function, external or indwelling catheter<br>6☐ loss of bowel and bladder function

Cerebral functions

The presence of depression and/or euphoria alone results in a score of 1 on the cerebral FS, but does not affect the EDSS score

Depression/euphoria

Patient complains of depression or is considered depressed or euphoric by the investigator or «significant other»

Decrease in mentation

mild	difficulties apparent to patient and «significant other» such as impaired ability to follow a rapid course of association and of surveying complex matters, impaired judgement in certain demanding situations, able to handle the daily routine, but no tolerance for additional stressors, intermittently symptomatic to even normal levels of stress, reduced performance, tendency toward negligence due to obliviousness or fatigue.<br>However, not apparent while taking the history or performing the routine neurological examination.
moderate	definite abnormalities on formal mental status testing, but still oriented to time, place and person
marked	not oriented in 1 or 2 spheres of time, place or person, marked effect on lifestyle
dementia	confusion and/or complete disorientation

Fatigue

mild	not interfering with daily activities
moderate	interfering but not limiting daily activities for more than 50%
severe	significantly limiting daily activities (> 50% reduction)

NEUROLOGICAL EXAMINATION (EDSS) *(Continued)*	
**Cerebral functions / mental status examination –** neurological examination	
Depression	0☐ none<br>1☐ present
Euphoria	0☐ none<br>1☐ present
Decrease in mentation	0☐ none<br>1☐ mild<br>2☐ moderate<br>3☐ marked<br>4☐ dementia
Fatigue	0☐ none<br>1☐ mild<br>2☐ moderate<br>3☐ severe
**Kurtzke Functional System Score**	
Cerebral functions	0☐ normal<br>1☐ mood alteration only / mild fatigue (does not affect EDSS score)<br>2☐ mild decrease in mentation / moderate or severe fatigue<br>3☐ moderate decrease in mentation<br>4☐ marked decrease in mentation<br>5☐ dementia

Ambulation

Actual walking distance without assistance must be observed up to 500 m. Actual walking distance with assistance must be observed up to 150 m (if possible).
In the definitions of EDSS grades 6.0 and 6.5 both a description of assistance required and of the walking range are included. In general, the distinction of bilateral versus unilateral assistance required to walk overrules the walking range. However, the following exceptions apply: If a patient is able to walk considerably longer than 100 m with two sticks, crutches or braces he is in grade 6.0. If a patient is able to walk more than 10 m and up to 100 m with two sticks, crutches or braces only he is in grade 6.5. If a patient needs assistance by another person (as opposed to one stick, crutch or brace) this is regarded as equivalent to bilateral assistance.

NEUROLOGICAL EXAMINATION (EDSS) *(Continued)*

Ambulation – neurological examination

Walking distance as reported (without help or sticks)	└───┘ meter / in └───┘ minutes
Actual distance without rest or assistance	1☐ <100 m<br>2☐ ≥ 100 m - ≤ 200 m<br>3☐ > 200 m - ≤ 300 m<br>4☐ > 300 m - ≤ 500 m<br>5☐ > 500 m but restricted as compared to average normal performance<br>6☐ unrestricted

if unable to walk 100 m without constant assistance

distance with unilateral assistance	└───┘ meter
distance with bilateral assistance	└───┘ meter

Kurtzke expanded disability scale (EDSS)

- EDSS steps below 4 refer to patients who are able to walk >500 m without assistance and the precise step is defined by the functional systems (FS) score(s).
- EDSS steps between 4.0 and 5.0 are defined by both FS-scores and walking range. In general, the worst of both should determine the score.
- Steps 5.5-8.0 are exclusively defined by ability to ambulate, or use wheelchair, and need of assistance for transfer.
- EDSS (0–4) should not change by 1.0 step unless there is a change in same direction of at least one step in at least one FS.
- EDSS should not be lower than each of FS (excepted visual and bowel /bladder FS).

For calculation of the EDSS, the score of the visual and of the bowel/bladder FS are to be converted as follows:
- Visual FS: 6 = 4 / 5 = 3 / 4 = 3 / 3 = 2 / 2 = 2 / 1=1
- Bowel/bladder FS: 6 = 5 / 5 = 4 / 4 = 3 / 3 = 3 / 2 = 2 / 1=1

NEUROLOGICAL EXAMINATION (EDSS) *(Continued)*
**Kurtzke Expanded Disability Status Scale (EDSS)**
**0.0** = normal neurological exam (all grade 0 in FS*)<br>**1.0** = no disability, minimal signs in one FS* (i.e., grade 1)<br>**1.5** = no disability, minimal signs in more than one FS* (more than one FS grade 1)<br>**2.0** = minimal disability in one FS (one FS grade 2, others 0 or 1)<br>**2.5** = minimal disability in two FS (two FS grade 2, others 0 or 1)<br>**3.0** = moderate disability in one FS (one FS grade 3, others 0 or 1) or mild disability in three or four FS (three or four FS grade 2, others 0 or 1) though fully ambulatory<br>**3.5** = fully ambulatory but with moderate disability in one FS (one grade 3) and one or two FS grade 2; or two FS grade 3; or five FS grade 2 (others 0 or 1)<br>**4.0** = ambulatory without aid or rest for > 500 m; up and about some 12 hours a day despite relatively severe disability consisting of one FS grade 4 (others 0 or 1), or combinations of lesser grades exceeding limits of previous steps<br>**4.5** = ambulatory without aid or rest for > 300 m; up and about much of the day; characterised by relatively severe disability usually consisting of one FS grade 4 or combinations of lesser grades exceeding limits of previous steps<br>**5.0** = ambulatory without aid or rest for > 200 m (usual FS equivalents are one grade 5 alone, others 0 or 1; or combinations of lesser grades usually exceeding specifications for step 4.5)<br>**5.5** = ambulatory without aid or rest > 100 m<br>**6.0** = unilateral assistance (cane or crutch) required to walk at least 100 m with or without resting<br>**6.5** = constant bilateral assistance (canes or crutches) required to walk at least 20 m without resting<br>**7.0** = unable to walk 20 m even with aid, essentially restricted to wheelchair; wheels self and transfers alone; up and about in wheelchair some 12 h a day<br>**7.5** = unable to take more than a few steps; restricted to wheelchair; may need some help in transfer and in wheeling self<br>**8.0** = essentially restricted to bed or chair or perambulated in wheelchair, but out of bed most of day; retains many self-care functions; generally has effective use of arms<br>**8.5** = essentially restricted to bed much of the day; has some effective use of arm(s); retains some selfcare functions<br>**9.0** = helpless bed patient; can communicate and eat<br>**9.5** = totally helpless bed patient; unable to communicate effectively or eat /swallow<br>**10.0** = death due to MS<br>* Mental function’s grade 1 does not contribute to EDSS-step definitions

**EDSS Score**	└─┴─┘.└─┘

Signature of investigator	
Date: └─┴─┘ └─┴─┴─┘ └─┴─┴─┴─┘<br>DD MON YYYY	Investigator's signature:

17.2 Mc Donald Diagnostic Criteria for MS

2005 Revised McDonald Diagnostic Criteria for MS

What Is An Attack?

- Neurological disturbance of kind seen in MS
- Subjective report or objective observation
- 24 hours duration, minimum
- Excludes pseudoattacks, single paroxysmal episodes

Determining Time Between Attacks

- 30 days between onset of event 1 and onset of event 2

What is a Positive MRI?[2,3]

3 out of 4 of the following:

- 1 Gd-enhancing brain or cord lesion ***or*** 9 T2 hyperintense brain and/or cord lesions if there is no Gd-enhancing lesion
- 1 or more brain infratentorial or cord lesion
- 1 or more juxtacortical lesion
- 3 or more periventricular lesions

Note: Individual cord lesions can contribute along with individual brain lesions to reach required number of T2 lesions

What Provides MRI Evidence of Dissemination in Time?

- A Gd-enhancing lesion detected in a scan done at least 3 months after onset of initial clinical event at a site different from the initial event

or

- A new T2 lesion detected in a scan done at any time compared to a reference scan done at least 30 days after initial clinical event

What is Positive CSF?

Oligoclonal IgG bands in CSF (and not serum) ***or*** elevated IgG index

What is Positive VEP?

Delayed but well-preserved wave form

The 2005 Revisions to the McDonald Diagnostic Criteria for MS

CLINICAL PRESENTATION	ADDITIONAL DATA NEEDED FOR MS DIAGNOSIS
2 or more attacks; objective clinical evidence of 2 or more lesions	• None
2 or more attacks; objective clinical evidence of 1 lesion	• Dissemination in space, demonstrated by:<br>➔ MRI<br>OR<br>➔ 2 or more MRI detected lesions consistent with MS plus positive CSF<br>OR<br>➔ Await further clinical attack implicating a different site
1 attack; objective clinical evidence of 2 or more lesions	• Dissemination in time, demonstrated by:<br>➔ MRI<br>OR<br>➔ Second clinical attack
1 attack; objective clinical evidence of 1 lesion<br>(monosymptomatic presentation; clinically isolated syndrome)	• Dissemination in space, demonstrated by:<br>➔ MRI<br>OR<br>➔ 2 or more MRI-detected lesions consistent with MS plus positive CSF<br>AND<br>• Dissemination in time, demonstrated by:<br>➔ MRI<br>OR<br>➔ Second clinical attack
Insidious neurological progression suggestive of MS	• One year of disease progression (retrospectively or prospectively determined)<br>AND<br>• Two out of three of the following:<br>a. Positive brain MRI (9 T2 lesions or 4 or more T2 lesions with positive visual evoked potentials;<br>b. Positive spinal cord MRI (two or more focal T2 lesions);<br>c. Positive CSF

17.3 Common Terminology Criteria for Adverse Events (CTCAE)

CTCAE V3 Publish Date: August 9, 2006 (abbreviated)

Quick Reference

The NCI Common Terminology Criteria for Adverse Events v3.0 is a descriptive terminology which can be utilized for Adverse Event (AE) reporting. A grading (severity) scale is provided for each AE term.

Components and Organization

CATEGORY

A CATEGORY is a broad classification of AEs based on anatomy and/or pathophysiology. Within each CATEGORY, AEs are listed accompanied by their descriptions of severity (Grade).

Each AE term is mapped to a MedDRA term and code. AEs are listed alphabetically within CATEGORIES.

Short AE Name

The 'SHORT NAME' column is new and it is used to simplify documentation of AE names on Case Report Forms.

Supra-ordinate Terms

A supra-ordinate term is located within a CATEGORY and is a grouping term based on disease process, signs, symptoms, or diagnosis. A supra-ordinate term is followed by the word *'Select'* and is accompanied by specific AEs that are all

ALSO CONSIDER

An 'ALSO CONSIDER' indicates additional AEs that are to be graded if they are clinically significant.

NAVIGATION NOTE

A 'NAVIGATION NOTE' indicates the location of an AE term within the CTCAE document. It lists signs/symptoms alphabetically and the CTCAE term will appear in the same CATEGORY unless the 'NAVIGATION NOTE' states differently.

Adverse Event Terms

An AE is any unfavorable and unintended sign (including an abnormal laboratory finding), symptom, or disease temporally associated with the use of a medical treatment or procedure that may or may *not* be considered related to the medical treatment or procedure. An AE is a term that is a unique representation of a specific event used for medical documentation and scientific analyses.

related to the supra-ordinate term. Supra-ordinate terms provide clustering and consistent representation of Grade for related AEs. Supra-ordinate terms are not AEs, are not mapped to a MedDRA term and code, cannot be graded and cannot be used for reporting.

REMARK

A 'REMARK' is a clarification of an AE.

Grades

Grade refers to the severity of the AE. The CTCAE v3.0 displays Grades 1 through 5 with unique clinical de scriptions of severity for each AE based on this general guideline:

A Semi-colon indicates 'or' within the description of the grade.

An 'Em dash' (—) indicates a grade not available.

Not all Grades are appropriate for all AEs. Therefore, some

Grade 5

Grade 5 (Death) is not appropriate for some AEs and therefore is not an option.

The DEATH CATEGORY is new. Only one Supra-

Grade 1 Mild AE<br><br>Grade 2 Moderate AE<br><br>Grade 3 Severe AE<br><br>Grade 4 Life-threatening or disabling AE<br><br>Grade 5 Death related to AE	AEs are listed with fewer than five options for Grade selection.	ordinate term is listed in this CATEGORY: 'Death not associated with CTCAE term – *Select*' with 4 AE options: Death NOS; Disease progression NOS; Multi-organ failure; Sudden death.<br><br>Important:<br><br>• Grade 5 is the only appropriate Grade<br><br>• This AE is to be used in the situation where a death:<br><br>1. cannot be reported using a CTCAE v3.0 term associated with Grade 5, or<br><br>2. cannot be reported within a CTCAE CATEGORY as 'Other (Specify)'

Contents

(1) BLOOD/BONE MARROW
(2) COAGULATION
(3) METABOLIC/LABORATORY
(4) ENDOCRINE

BLOOD/BONE MARROW

Adverse Event	Short Name	Grade				
		1	2	3	4	5
**CD4 count**	CD4 count	<LLN – 500/mm^3<br><LLN – 0.5 x 10^9 /L	<500 - 200/mm^3<br><0.5 – 0.2 x 10^9 /L	<200 - 50/mm^3<br><0.2 – 0.05 x 10^9 /L	<50 /mm^3<br><0.05 x 10^9 /L	
**Hemoglobin**	Hemoglobin	<LLN - 10.0 g/dL<br><LLN - 100 g/L<br><LLN - 6.2 mmol/L	8.0 - <10.0 g/dL<br>80 - <100 g/L<br>4.9 - <6.2 mmol/L	6.5 - <8.0 g/dL<br>65 - <80 g/L<br>4.0 - <4.9 mmol/L	<6.5 g/dL<br><65 g/L<br><4.0 mmol/L	Death
**Leukocytes** (total WBC)	Leukocytes	<LLN - 3.0 x 10^9/L<br><LLN - 3000/mm^3	≥2.0 - <3.0 x 10^9/L<br>≥2000 - <3000/mm^3	≥1.0 - <2.0 x 10^9/L<br>≥1000 - <2000/mm^3	<1.0 x 10^9/L<br><1000/mm^3	Death
**Lymphopenia**	Lymphopenia	<LLN - 1.0 x 10^9/L<br><LLN - 1000/mm^3	≥0.5 - <1.0 x 10^9/L<br>≥500 - <1000/ mm^3	<0.5 x 10^9/L<br><500/mm^3	—	Death
**Neutrophils/ granulocytes** (ANC/AGC)	Neutrophils	≥1.5 - <2.0 x 10^9/L<br>≥1500 - <2000/mm^3	≥1.0 - <1.5 x 10^9/L<br>≥1000 - <1500/mm^3	≥0.5 - <1.0 x 10^9/L<br>≥500 - <1000/mm^3	<0.5 x 10^9/L<br><500/mm^3	Death
**Platelets**	Platelets	<LLN - 75.0 x 10^9/L<br><LLN - 75,000/mm^3	≥50.0 - <75.0 x 10^9/L<br>≥50,000-<75,000/mm^3	≥25.0 - <50.0 x 10^9/L<br>≥25,000-<50,000/mm^3	<25.0 x 10^9/L<br><25,000//mm^3	Death
**Blood - Other** (Specify, _____)	Blood - Other (Specify, _________)	mild	moderate	severe	life-threatening; disabling	Death

METABOLIC/LABORATORY

Adverse Event	Short Name	Grade				
		1	2	3	4	5
**SGPT** (ALT) (serum glutamic pyruvic transaminase)	ALT	>ULN - 2.5 x ULN	>2.5 - 5.0 x ULN	>5.0 - 20.0 x ULN	>20.0 x ULN	—

Adverse Event	Short Name	Grade				
		1	2	3	4	5
**SGOT** (AST) (serum glutamic oxaloacetic transaminase)	AST	>ULN - 2.5 x ULN	>2.5 - 5.0 x ULN	>5.0 - 20.0 x ULN	>20.0 x ULN	—
**Alkaline phosphatase**	AP	>ULN - 2.5 x ULN	>2.5 - 5.0 x ULN	>5.0 - 20.0 x ULN	>20.0 x ULN	—
**GGT** (γ-Glutamyl transpeptidase)	GGT	>ULN - 2.5 x ULN	>2.5 - 5.0 x ULN	>5.0 - 20.0 x ULN	>20.0 x ULN	Death
**Bilirubin (total)** (hyperbilirubinemia)	Bilirubin	>ULN - 1.5 x ULN	>1.5 - 3.0 x ULN	>3.0 - 10.0 x ULN	>10.0 x ULN	—
**Albumin**, serum-low (Hypoalbuminemia)	Hypoalbuminemia	<LLN - 3 g/dL	≥2 - <3 g/dL	<2 g/dL	—	Death
**Sodium**, serum high	Hypernatremia	>ULN - 150 mmol/L	>150 - 155 mmol/L	>155 - 160 mmol/L	>160 mmol/L	Death
**Sodium**, serum low	Hyponatremia	<LLN - 130 mmol/L	—	120 - <130 mmol/L	<120 mmol/L	Death
**Calcium**, serum high	Hypercalcemia	>ULN - 11.5 mg/dL >ULN - 2.9 mmol/L	>11.5 - 12.5 mg/dL >2.9 - 3.1 mmol/L	>12.5 - 13.5 mg/dL >3.1 - 3.4 mmol/L	>13.5 mg/dL >3.4 mmol/L	Death
**Calcium**, serum low	Hypocalcemia	<LLN - 8.0 mg/dL <LLN - 2.0 mmol/L	7.0 - <8.0 mg/dL 1.75 - <2.0 mmol/L	6.0 - <7.0 mg/dL 1.5 - <1.75 mmol/L	<6.0 mg/dL <1.5 mmol/L	Death
**Potassium**, serum high	Hyperkalemia	>ULN - 5.5 mmol/L	>5.5 - 6.0 mmol/L	>6.0 - 7.0 mmol/L	>7.0 mmol/L	Death
**Potassium**, serum low	Hypokalemia	<LLN - 3.0 mmol/L	—	2.5 - <3.0 mmol/L	<2.5 mmol/L	Death

**Adverse Event**	**Short Name**	**Grade**				
		**1**	**2**	**3**	**4**	**5**
**Magnesium**, serum high	Hypermagnesemia	>ULN - 3.0 mg/dL >ULN - 1.23 mmol/L	—	- >3.0 - 8.0 mg/dL >1.23 - 3.30 mmol/L	>8.0 mg/dL >3.30 mmol/L	Death
**Magnesium**, serum low	Hypomagnesemia	<LLN - 1.2 mg/dL <LLN - 0.5 mmol/L	0.9 - <1.2 mg/dL 0.4 - <0.5 mmol/L	0.7 - <0.9 mg/dL 0.3 - <0.4 mmol/L	<0.7 mg/dL <0.3 mmol/L	Death
**Glucose**, serum high	Hyperglycemia	>ULN - 160 mg/dL >ULN - 8.9 mmol/L	>160 - 250 mg/dL >8.9 - 13.9 mmol/L	>250 - 500 mg/dL >13.9 - 27.8 mmol/L	>500 mg/dL >27.8 mmol/L or acidosis	Death
**Glucose**, serum low	Hypoglycemia	<LLN - 55 mg/dL <LLN - 3.0 mmol/L	40 - <55 mg/dL 2.2 - <3.0 mmol/L	30 - <40 mg/dL 1.7 - <2.2 mmol/L	<30 mg/dL <1.7 mmol/L	Death
**Holesterol**, serum high	Hypercholesterol-emia	>ULN - 300 mg/dL >ULN - 7.75 mmol/L	>300 - 400 mg/dL >7.75 - 10.34 mmol/L	>400 - 500 mg/dL >10.34 - 12.92 mmol/L	>500 mg/dL >12.92 mmol/L	Death
**Creatinine**	Creatinine	>ULN – 1.5 x ULN	>1.5 – 3.0 x ULN	>3.0 – 6.0 x ULN	>6.0 x ULN	Death
**Uric acid,** Serum hight	Hiperuricemia	>ULN - 10 mg/dL ≤0.59 mmol/L without physiologic consequences	—	>ULN - 10 mg/dL ≤0.59 mmol/L with physiologic consequences	>10 mg/dL >0.59 mmol/L	Death
**Metabolic/** Laboratory Other (Specify,_____)	Metabolic/ Laboratory Other (Specify,-_________)	mild	moderate	severe	life-threatening; disabling	Death

COAGULATION

Adverse Event	Short Name	Grade				
		1	2	3	4	5
**Partial thrombo-plastin time**	PPT	>ULN - ≥1.5 x ULN	>1.5 - ≥2 x ULN	>2 x ULN	—	Death
**Prothrombin time***	(PT, Quick)	>ULN - ≥1.5 x ULN	>1.5 - ≥2 x ULN	>2 x ULN	—	Death
**Coagulation – Other** (Specify, _____)	Coagulation – Other (Specify,_________)	mild	moderate	severe	life-threatening; disabling	Death

ENDOCRINE

Adverse Event	Short Name	Grade				
		1	2	3	4	5
**Thyroid function**, hight (hyperthyroidism, thyrotoxicosis)	Hyperthyroidism	Asymptomatic, intervention not indicated	Symptomatic; not interfering with ADL; thyroid suppression therapy indicated	Symptoms interfering with ADL; hospitalization indicated	Life threatening consequences (e.g., thyroid storm)	Death
**Thyroid function**, low (hypothyroidism)	Hypothyroidism	Asymptomatic, intervention not indicated	Symptomatic; not interfering with ADL; thyroid replacement therapy indicated	Symptoms interfering with ADL; hospitalization indicated	Life threatening myxedema coma	Death
**Endocrine – Other** (Specify,_____	Endocrine – Other (Specify,________	Mild	Moderate	Severe	Life-threatening; disabling	Death

Adverse Event	Short Name	Grade
_)	_)	

* **Prothrombin time** is taken from CTC V2
